# Potent and biased agonists of class B1 GPCRs from a heterochiral design strategy

**DOI:** 10.1038/s41557-026-02182-x

**Published:** 2026-06-16

**Authors:** Ruslan Gibadullin, Lauren My-Linh Tran, Jiani Niu, Rylie K. Morris, Ariel J. Kuhn, John A. Mannone, Tae Wook Kim, Giuseppe Deganutti, Samuel H. Gellman

**Affiliations:** 1 Department of Chemistry, University of Wisconsin, Madison, WI USA; 2 Centre for Discoveries in Life Sciences, Coventry University, Coventry, UK; 3 Present Address: Department of Chemistry, The Scripps Research Institute, La Jolla, CA USA

**Keywords:** Peptides, Receptor pharmacology, Computational chemistry

## Abstract

Glucagon is used to treat severe hypoglycaemia in patients with diabetes, and PTH(1–34), an N-terminal fragment of parathyroid hormone, is used to treat osteoporosis. Each peptide activates a cognate G-protein-coupled receptor (GPCR). High-resolution structures of peptide–receptor complexes show that glucagon and PTH(1–34) are fully α-helical in the bound state. Here we describe a design strategy that destabilizes the helical conformation of glucagon or PTH(1–34) but nevertheless delivers potent agonists of each receptor. Replacement of multiple l-α-amino-acid residues with d-α-amino-acid residues, at selected positions, generates heterochiral analogues of both glucagon and PTH(1–34) that approach the all-l peptide in receptor activation potency (cAMP production). The heterochiral agonists are biased away from β-arrestin recruitment relative to the all-l peptide. The glucagon analogues are also biased away from receptor internalization relative to glucagon itself. These unanticipated findings for two dissimilar class B1 GPCRs raise the prospect that heterochiral design will be impactful for other members of this receptor family, which includes multiple therapeutic targets.

**Figure:**
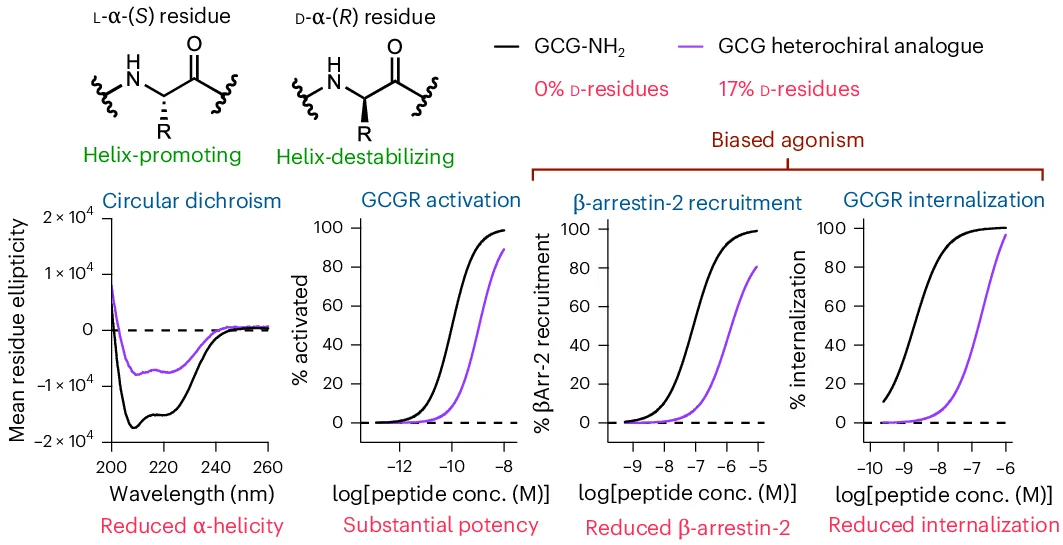

## Abstract (web summary)

Many peptides that activate class B1 GPCRs, such as the glucagon receptor (GCGR) or the parathyroid hormone receptor-1 (PTH1R4), are α-helical in the active receptor-bound form. Now, despite destabilizing the helical conformation of parent glucagon and PTH agonists, the presented design strategy generates potent and biased heterochiral analogues by replacing select l-α-amino-acid residues with d-α-amino acids.

## Main

G-protein-coupled receptors (GPCRs) mediate the flow of information between the environment and the interior of eukaryotic cells. Class B1 GPCRs, which have critical roles in physiology, are activated by polypeptide hormones^1^. These long peptides (≥29 residues) engage a large orthosteric site on the receptor comprising the core of the transmembrane domain (TMD) and a surface on the extracellular domain (ECD)^2^. Clinical efforts directed towards class B1 GPCRs have produced multiple therapeutic agents, including agonists of the glucagon-like peptide-1 (GLP-1) receptor to treat type 2 diabetes and obesity, agonists of the glucagon receptor (GCGR) to treat hypoglycaemia, and agonists of the parathyroid hormone receptor-1 (PTH1R) to treat osteoporosis^1,3^.

The biomedical importance of class B1 GPCRs has motivated extensive exploration of synthetic hormone analogues to achieve goals including altered signalling profiles (‘biased agonism’) and improved pharmacokinetics. Most reported modifications have involved replacing native l-α-amino-acid residues with non-native l-α residues or achiral residues. For example, Ala at position 2 (Ala2) in GLP-2 is replaced with Gly in teduglutide, a GLP-2 analogue used for the treatment of short bowel syndrome (SBS), to minimize cleavage by dipeptidyl peptidase-4 (DPP4)^4,5^. Semaglutide, a GLP-1 analogue approved to treat type 2 diabetes and obesity, contains three replacements^6^: Ala8 is replaced with 2-aminoisobutyric acid (Aib) to suppress DPP4 activity; Lys26 is replaced with an l-Lys derivative bearing a fatty-acid-like appendage to promote albumin binding and thereby hinder renal excretion; and Lys34 is replaced with l-Arg, to enable selective acylation at Lys26.

The intrinsic modularity of peptides enables facile incorporation of subunits beyond those derived from l-α-amino acids, opening a vast realm of hormone analogues to be explored for distinctive properties and activity profiles^7–16^. Such compounds could provide distinct tools for characterizing signal transduction mechanisms and might ultimately lead to improved therapeutic agents. The appeal of non-traditional subunits is tempered, however, by the potential for conformational changes, which could interfere with receptor activation. Many of the endogenous hormones and potent synthetic analogues are largely or entirely α-helical when bound to class B1 GPCRs^17–23^; l-α-amino-acid residue replacements that do not support a right-handed α-helix or similar conformation would therefore seem to be counterproductive.

Replacing l-α-amino-acid residues with d-α-amino-acid residues destabilizes the right-handed α-helix^24–26^. (The ‘d’ in d-α-amino-acid stands for dextrorotatory, which refers to the stereochemistry of the amino acid; d-α-amino-acids are mirror images of naturally occurring l-α-amino-acids (the ‘l’ denotes levorotatory)). Studies of hormone analogues containing a single l-to-d replacement have generally been consistent with the expectation that decreased α-helix propensity should correlate with lower agonist activity^27–29^. d-scan studies based on a potent derivative of GLP-2^27^ or glucagon^28^ revealed that many diastereomers resulting from a single l-to-d replacement displayed diminished potency relative to the homochiral peptide, as judged by stimulation of intracellular 3′,5′-cyclic adenosine monophosphate (cAMP) production. In each case, however, single l-to-d replacement was tolerated at some sites. Replacing the native Gly4 of glucagon with d-Phe enhanced potency for raising plasma glucose levels in rats^30^. Evolution has harnessed l-to-d substitution at a single site in a short neuropeptide to achieve selectivity between two related class A GPCRs^31^.

We describe an unexpected strategy for generating potent analogues of class B1 GPCR peptide agonists; these analogues manifest distinctive activity profiles. Despite the evidence that α-helicity in receptor-bound agonists is important for signal transduction via these receptors^17–23^, we have discovered analogues containing three or more d-α-amino-acid residues distributed across the sequence that retain potent agonist activity for two dissimilar class B1 GPCRs, as measured by stimulation of intracellular cAMP production. In each case, the heterochiral analogues display β-arrestin recruitment profiles distinct from those of the all-l peptide. Collectively, our findings suggest a path to previously unanticipated and potentially valuable analogues of diverse peptide hormones.

## Results and discussion

### Potent heterochiral glucagon analogues

Our interest in implementing multiple l-to-d replacements was motivated by the prospect that heterochiral agonists could enable future efforts to probe the contribution of agonist dynamics in the receptor-bound state to signal transduction^9^. We selected glucagon for exploratory studies because the results of an alanine scan^32^ and a d-residue scan^28^ have been reported. In both cases, the effect of each single-site modification of glucagon was evaluated in terms of potency for GCGR activation, as manifested by intracellular cAMP production. We reasoned that sites tolerant of both alanine substitution and d-residue substitution should be most favourable for our evaluation of multiple l-to-d substitutions. Almost all positions that meet these criteria occur in the C-terminal half of glucagon, residues 16–29.

Gly4 was considered as a site for d substitution near the N terminus. Glycine is conserved as the fourth residue from the N terminus across the related hormones GLP-1, GLP-2 and gastric inhibitory polypeptide (GIP), and glycine is found at this position in the venom-derived GLP-1R agonist exendin-4^33,34^. We have shown that native potency is retained when this Gly is replaced with d-Ala in GLP-1, exendin-4 or GLP-2^9,35^.

**Fig. 1:**
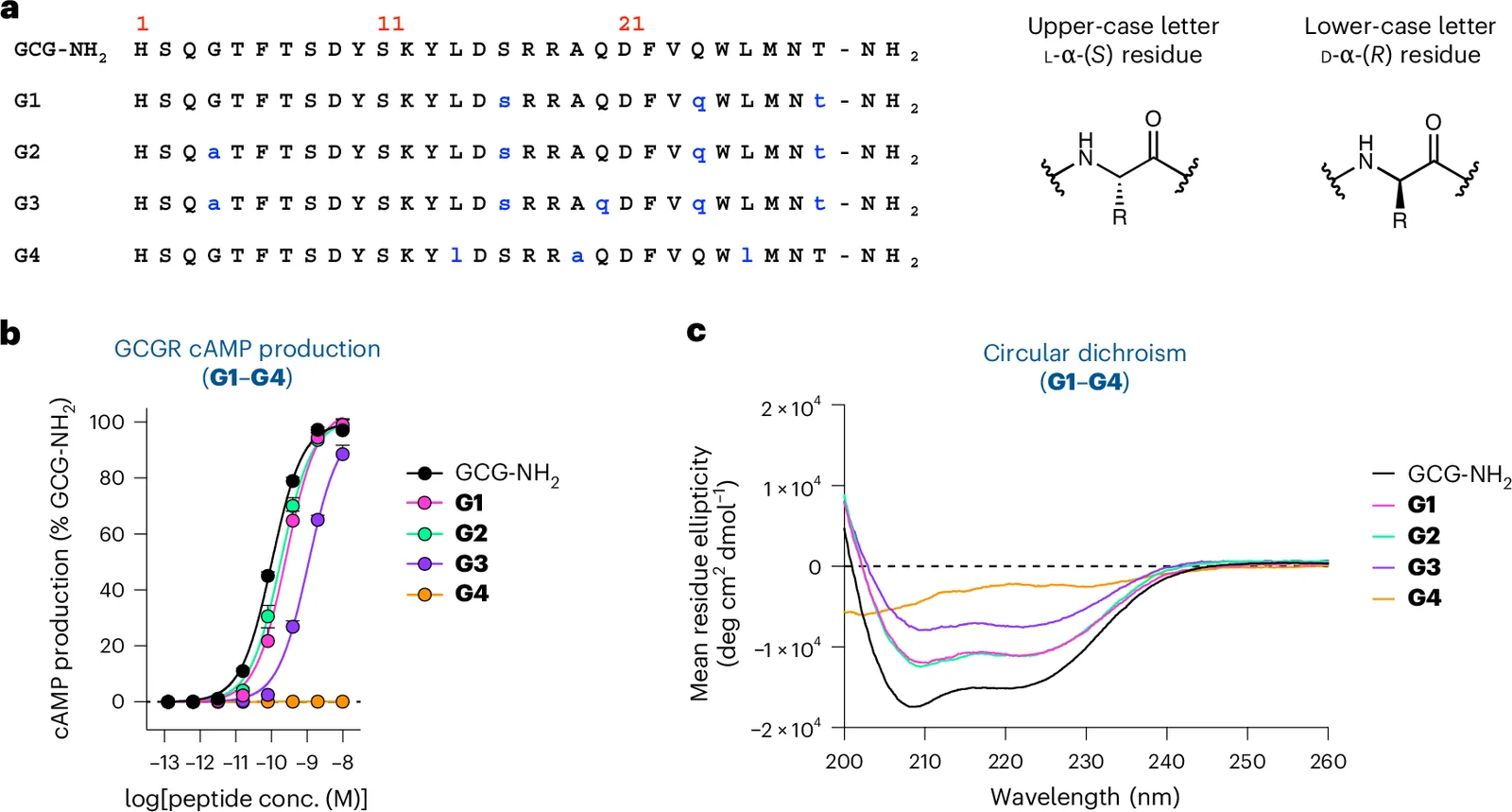
Heterochiral glucagon analogues display native-like potency at the GCGR, despite diminished α-helicity. **a**, Sequences of glucagon (GCG-NH_2_) and heterochiral analogues **G1**–**G4**. Sites of d-residue replacements in peptides **G1**–**G4** are indicated by lower-case letters and highlighted in blue. **b**, Activation of the GCGR by GCG-NH_2_ and analogues. The assay measured levels of cAMP production in HEK293 cells transiently expressing the GCGR and stably expressing the cAMP-sensing protein GloSensor. Data points represent the average of ≥5 independent experiments (*n* = 13 for GCG-NH_2_, *n* = 8 for **G1**, *n* = 8 for **G2**, *n* = 8 for **G3**, *n* = 5 for **G4**). Data for all peptides (**G1**–**G4**) were normalized against GCG-NH_2_. Error bar uncertainties are expressed as s.e.m. **c**, Circular dichroism data for GCG-NH_2_ and heterochiral analogues (**G1**–**G4**) measured in phosphate-buffered saline (PBS), pH 7.4, containing 20 vol% 2,2,2-trifluoroethanol (TFE). Source data

Heterochiral glucagon analogue **G1**, containing l-to-d substitution at Ser16, Gln24 and Thr29, was only approximately threefold less potent than glucagon itself in activating the GCGR, as detected by cAMP production in HEK293 cells that over-express the human GCGR and the cAMP-sensing protein GloSensor^36^ (Fig. 1a,b and Table 1). Augmenting these three modifications with Gly4-to-d-Ala substitution (**G2**) did not diminish the potency (Fig. 1a,b and Table 1). Introducing a fifth d-residue at Gln20 led to a modest potency decline: the half-maximal effective concentration (EC_50_) for **G3** was 1.1 nM, which is ~11-fold higher than that of glucagon (Fig. 1a,b and Table 1). **G1**–**G3** achieved a maximal cAMP level near or comparable to that of glucagon. We note that each analogue was prepared in the C-terminal amide form. Natural glucagon is a C-terminal acid (GCG-OH), but we found that the C-terminal amide derivative of glucagon (GCG-NH_2_) retained full native potency at the GCGR (Supplementary Fig. 1); GCG-NH_2_ was used for our comparisons.

**Table 1:** cAMP production potency and maximal response values for GCG-NH_2_ and analogues

Peptide	GCGR cAMP production			
	pEC_50_	EC_50_ (nM)	EC_50_ relative	% Max
GCG-NH_2_	10.0 ± 0.02	0.10	1	100 ± 1
**G1**	9.6 ± 0.04	0.26	3	104 ± 2
**G2**	9.7 ± 0.04	0.18	2	101 ± 2
**G3**	9.0 ± 0.03	1.10	11	88^a^
**G4**	NR	NR	NR	0

pEC_50_, EC_50_ and % Max values are derived from ≥5 independent experiments (data in Fig. 1b). pEC_50_ indicates the negative logarithm of the EC_50_. EC_50_ relative indicates the cAMP potency normalized to GCG-NH_2_ (GCG-NH_2_ analogue/GCG-NH_2_). pEC_50_ and % Max uncertainties are expressed as s.e.m. NR, no response.

^a^The value shown represents the activity measured at the highest agonist concentration evaluated—the dose–response curve failed to reach a maximum.

Source data

Glucagon is largely disordered in dilute aqueous solution, according to NMR and circular dichroism (CD) analyses^37–40^. Aqueous solutions of glucagon have a well-known propensity towards fibril formation upon standing^41–44^. We used CD in aqueous buffer containing 20 vol% 2,2,2-trifluoroethanol (TFE) to compare the conformational behaviour of GCG-NH_2_ and heterochiral analogues **G1**–**G3**. Inclusion of TFE is known to enhance the intrinsic secondary structure preferences of peptides^45^. GCG-NH_2_ and **G1**–**G3** each displayed minima near 208 and 222 nm, with intensities that indicate a partial population of α-helical conformations (Fig. 1c). The intensity of each minimum for **G1**–**G3** was diminished relative to GCG-NH_2_, which is consistent with the expectation that the incorporation of d-residues should destabilize the right-handed α-helix relative to the native homochiral peptide^24–26^. Replacing l-Ala with d-Ala destabilizes an α-helical conformation by ~1 kcal mol^−1^ (refs. ^46,47^). Given that cryo-electron microscopy (cryo-EM) indicates glucagon to be fully α-helical when bound to the GCGR–G_S_ complex^19^, it seems surprising that heterochiral analogues **G1**–**G3** approach glucagon itself in terms of their ability to activate the GCGR, despite their diminished propensity for α-helical folding.

Retention of agonist activity depended on the positions of l-to-d modification within glucagon. Diastereomer **G4**, with d-residues in place of Leu14, Ala19 and Leu26, did not stimulate cAMP production at 10 nM, a concentration at which **G1**–**G3** reached or approached maximum cAMP production (Fig. 1a,b and Table 1). This result could be explained in part by a further loss of α-helix propensity for **G4** relative to **G1**–**G3**, as determined by CD analysis (Fig. 1c). However, the side chains of Leu14, Ala19 and Leu26 make intimate contacts with the receptor surface^19^, and disruption or distortion of these contacts seems likely to destabilize the complex^19^ (Supplementary Fig. 2a). By contrast, the side chains of Ser16, Gln20, Gln24 and Thr29, sites of substitution in **G1**–**G3**, are less intimately engaged with the receptor according to cryo-EM (Supplementary Fig. 2a), which is consistent with tolerance of l-Ala mutation at each of these sites^32^.

### Glucagon analogues display altered signalling profiles

**Fig. 2:**
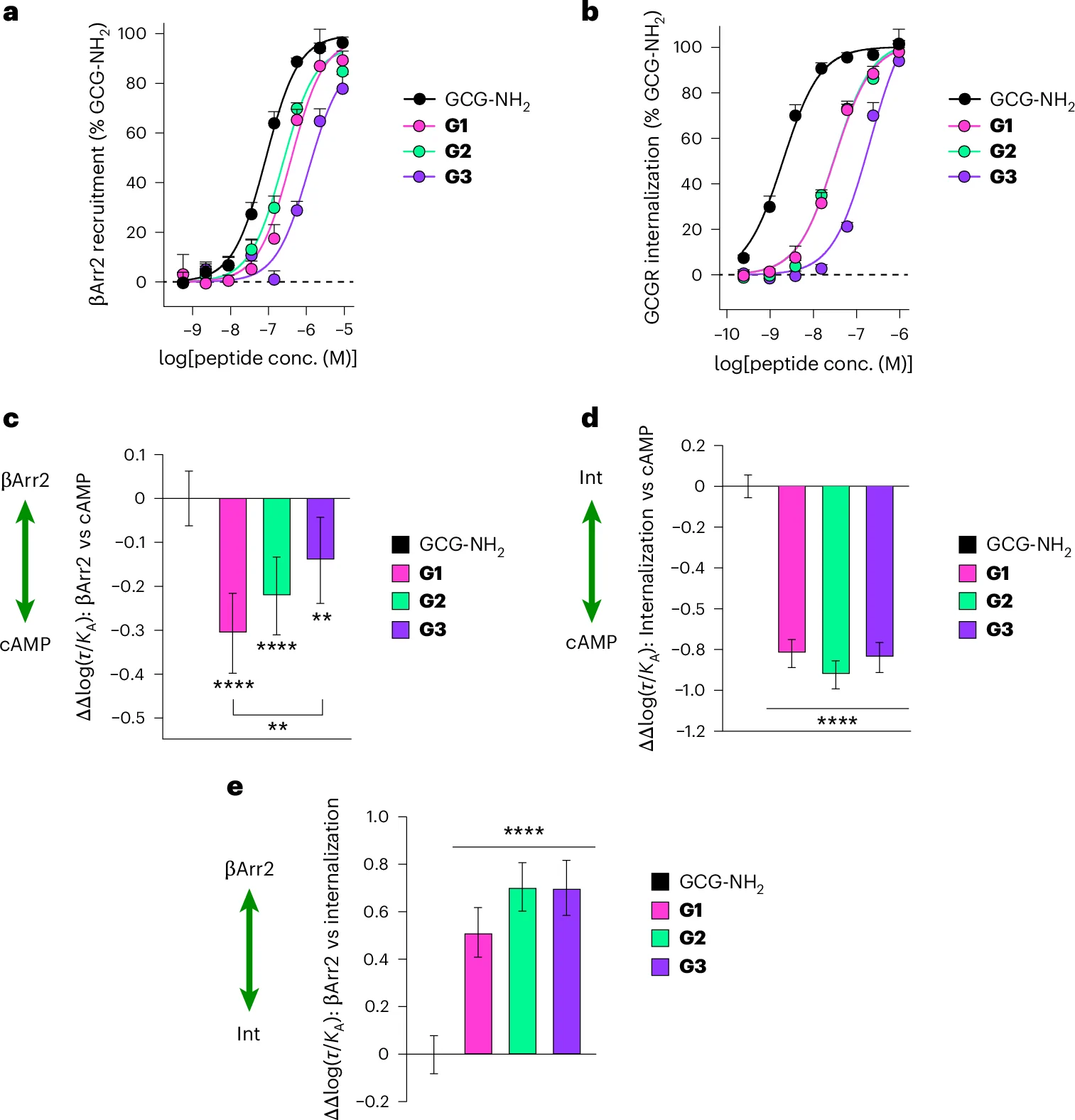
The effect of d-residues on β-arrestin-2 recruitment to the GCGR and on GCGR internalization. **a**, Recruitment of β-arrestin-2 (βArr2) to the GCGR induced by GCG-NH_2_ and heterochiral analogues. BRET assays were performed in HEK293FT cells transiently expressing the RLuc8-tagged GCGR and the GFP^2^-tagged β-arrestin-2 (R393E, R395E). Dose–response curves represent the average of ≥8 independent experiments (*n* = 13 for GCG-NH_2_, *n* = 8 for **G1**, *n* = 8 for **G2**, *n* = 9 for **G3**). **b**, GCGR internalization responses elicited by GCG-NH_2_ and analogues **G1**–**G3**. Assays were performed in HEK293 cells stably co-expressing the GCGR tagged with enzyme acceptor and a ProLink tag localized to endosomes. Data points represent the mean of four independent experiments. Data for all peptides (**a**,**b**) were normalized against GCG-NH_2_. All uncertainties are expressed as s.e.m. **c**, β-arrestin-2 versus cAMP bias factors (ΔΔlog(*τ*/*K*_A_)) calculated as the differences of the differences between transduction coefficients (log(*τ*/*K*_A_)) calculated according to the Black–Leff operational model from the dose–response curves in Fig. 1b (≥5 independent experiments) and panel **a** (≥8 independent experiments) for analogues **G1**–**G3** relative to GCG-NH_2_. A negative value indicates bias towards cAMP production. **d**, Internalization (Int) versus cAMP bias factors calculated from dose–response curves in Fig. 1b (≥5 independent experiments) and panel **b** (4 independent experiments) for analogues **G1**–**G3** relative to GCG-NH_2_. A negative value indicates bias towards cAMP production. **e**, β-arrestin-2 versus internalization bias factors calculated from dose–response curves in panels **a** (≥8 independent experiments) and **b** (4 independent experiments) for analogues **G1**–**G3** relative to GCG-NH_2_. A positive value indicates bias towards β-arrestin-2 recruitment to the GCGR. All bias factor uncertainties in **c**–**e** are expressed as derived mean ± propagated s.d. **P ≤ 0.01; ****P ≤ 0.0001; by one-way analysis of variance (ANOVA) followed by Dunnett test. *P* values in the form of an asterisk are shown for individual peptides in **c**–**e**. Source data

Stimulation of cAMP production via the GCGR reflects activation of the G_S_ heterotrimer, at least in part. To gain further understanding of the impact of l-to-d substitution on signal transduction, we examined recruitment of β-arrestin-2 and, in a separate assay, internalization of the GCGR induced by glucagon analogues **G1**–**G3**. These assays were conducted in HEK293 cells. β-arrestin-2 is reported to be critical for hepatic GCGR internalization^48^. Recruitment of GFP^2^-tagged β-arrestin-2 (R393E, R395E) to the RLuc8-tagged GCGR was monitored via bioluminescence resonance energy transfer (BRET); use of the (R393E, R395E) variant of β-arrestin-2 minimizes signal loss associated with receptor internalization^49^. The β-arrestin-2 recruitment potency of heterochiral agonists **G1**–**G3** was moderately diminished relative to that of glucagon (higher EC_50_ values relative to glucagon; Fig. 2a and Table 2). GCGR internalization was evaluated with cells that stably express the GCGR fused with enzyme acceptor and a ProLink tag localized to endosomes. Each of the heterochiral analogues **G1**–**G3** was less effective than glucagon at promoting receptor internalization (Fig. 2b and Table 2).

**Table 2:** Recruitment of β-arrestin-2 to the GCGR and GCGR internalization potency and maximal response values for GCG-NH_2_ and analogues

	GCGR β-arrestin-2 recruitment				GCGR internalization			
Peptide	pEC_50_	EC_50_ (μM)	EC_50_ relative	% Max	pEC_50_	EC_50_ (nM)	EC_50_ relative	% Max
GCG-NH_2_	7.1 ± 0.05	0.084	1	99 ± 2	8.7 ± 0.04	2.0	1	100 ± 2
**G1**	6.4 ± 0.09	0.39	5	98 ± 5	7.5 ± 0.06	31	16	102 ± 3
**G2**	6.6 ± 0.08	0.24	3	95 ± 4	7.5 ± 0.06	32	16	103 ± 3
**G3**	5.9 ± 0.1	1.2	14	78^a^	6.7 ± 0.08	210	110	94^a^

Left columns: The ability of GCG-NH_2_ and heterochiral analogues to induce β-arrestin-2 recruitment to the GCGR. pEC_50_, EC_50_ and % Max values are derived from ≥8 independent experiments (Fig. 2a). Right columns: GCGR internalization responses to GCG-NH_2_ and heterochiral analogues. pEC_50_, EC_50_ and % Max values are derived from 4 independent experiments (Fig. 2b). pEC_50_ values indicate the negative logarithm of the EC_50_. All EC_50_ relative values for β-arrestin-2 recruitment or GCGR internalization were normalized to the values for GCG-NH_2_ (heterochiral analogue/GCG-NH_2_). pEC_50_ and % Max uncertainties are expressed as s.e.m.

^a^The value shown represents the activity measured at the highest agonist concentration evaluated—the dose–response curve failed to reach a maximum.

Source data

We used the Black–Leff operational model^50,51^ to probe for signalling bias among the heterochiral analogues **G1**–**G3** relative to glucagon. This analysis included each possible pair among the three outcomes of receptor engagement: cAMP production, β-arrestin-2 recruitment and receptor internalization. Each bar in Fig. 2c–e represents a comparison of comparisons: the difference between the two activity measures for the analogue indicated relative to the corresponding difference for glucagon. Thus, glucagon defines an unbiased response for each pairwise consideration.

Heterochiral glucagon analogues **G1**–**G3** displayed a small but significant bias away from β-arrestin-2 recruitment relative to stimulation of cAMP production (presumably mediated via G_S_ activation; Fig. 2c). The bias factor diminished as the number of l-to-d substitutions increased (that is, **G1** manifested the strongest bias and **G3** the weakest, and the bias manifested by **G1** was statistically different from the bias exhibited by **G3**). Our effort to detect recruitment of β-arrestin-1 to the GCGR via the BRET assay format was unsuccessful, consistent with previous observations^52^.

Substantial biases away from GCGR internalization relative to cAMP production were evident for the heterochiral analogues (Fig. 2d). A qualitative parallel between bias away from β-arrestin-2 recruitment and bias away from receptor internalization has been reported for GLP-1 analogues^53,54^ and might be expected, because β-arrestin recruitment can mediate internalization^55^. However, some GPCRs can internalize in a β-arrestin-independent manner^56^, including the GLP-1R and GLP-2R. Heterochiral glucagon analogues **G1**–**G3** were biased away from receptor internalization relative to β-arrestin-2 recruitment (Fig. 2e), which suggests the operation of arrestin-independent internalization pathways for the GCGR.

### Molecular dynamics simulations

We conducted molecular dynamics (MD) simulations of GCGR complexes with glucagon or heterochiral analogue **G3** (Fig. 1a; C-terminal amide forms) to try to understand how a peptide with diminished α-helical propensity could nevertheless function as a potent agonist. Initial studies focused on the isolated peptides in aqueous solution, starting from the fully helical conformation of glucagon bound to the GCGR–G_S_ complex (PDB 6LMK)^19^. These simulations suggested that glucagon and **G3** display substantial helical population over residues 9–26, with lower helicity in the terminal regions (Supplementary Figs. 3a and 4). These conclusions contrast with experimental evidence that glucagon displays low helicity in aqueous solution^37–40^. The computational overestimation of glucagon α-helicity compared to experiments is consistent with the known tendency of molecular mechanics force fields to favour helical conformations^57,58^.

**Fig. 3:**
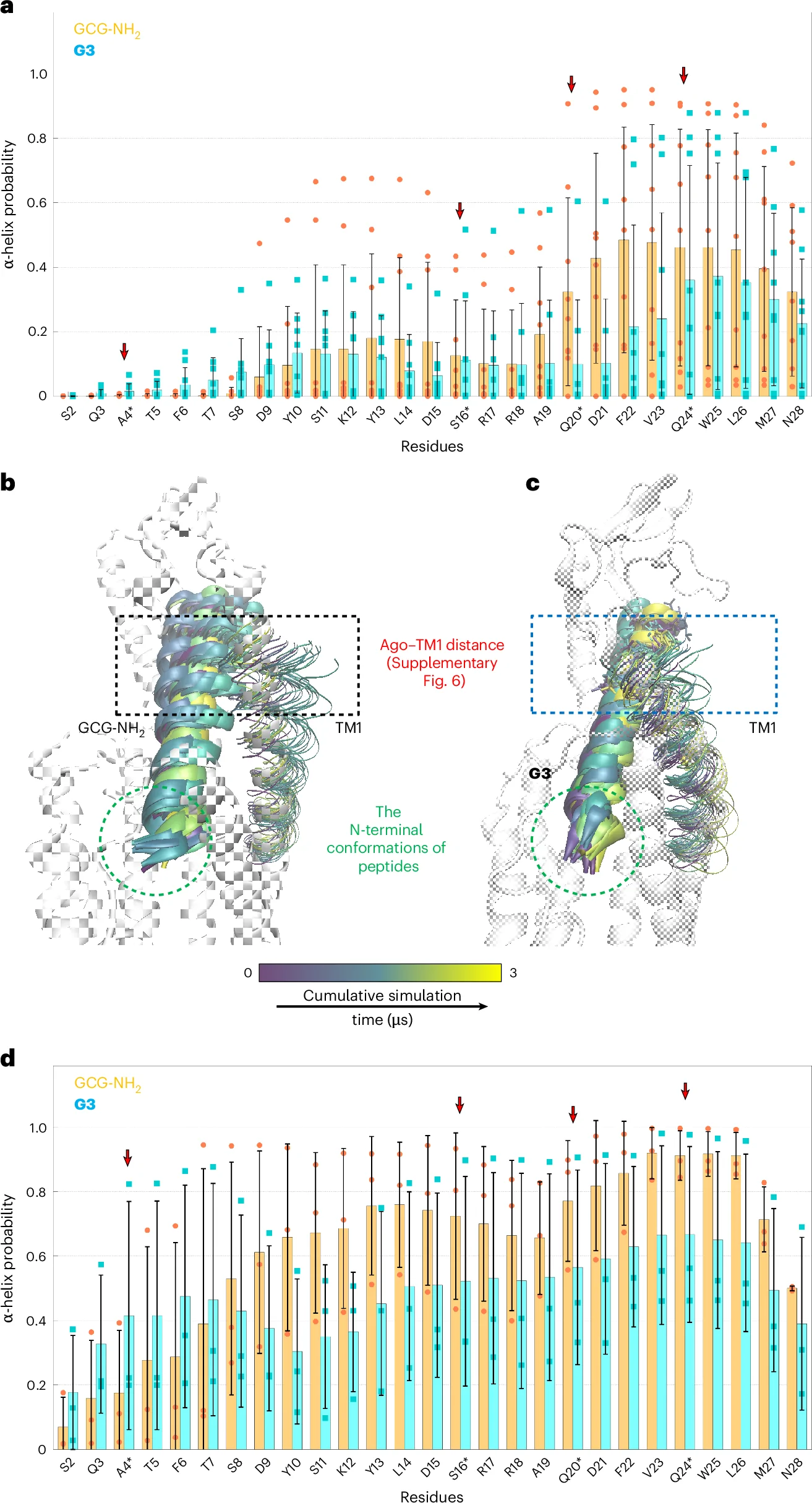
MD simulations of GCG-NH_2_ and G3. **a**, Comparison of the α-helix propensity of GCG-NH_2_ and **G3** during 27 µs of MD simulation (started from unfolded peptide conformations obtained using the MD enhanced sampling protocol; Methods) in simulated water solution; s.d. values are computed on 9 independent replicas of 3 µs each; red arrows and asterisks on residue labels (*x* axis) indicate residues 4, 16, 20 and 24 of the peptide. His1 and Thr29 are not shown because their backbone cannot form a secondary structure according to the database of secondary structure assignments (DSSP) algorithm. **b**,**c**, TM1 and GCG-NH_2_ (**b**) or **G3** (**c**) dynamics during MD simulations of peptide–GCGR complexes; the thin ribbon of TM1 and each peptide (thick ribbons) is coloured according to the cumulative simulation time of three replicas (second half of the three replicas, 3 µs in total), from dark blue (first frame) to yellow (last frame). The divergent conformations of the peptide N termini are highlighted by circular green dashed lines, and the dashed rectangles show the regions near Gln20 (GCG-NH_2_) or d-Gln20 (**G3**) that were considered for measuring the peptide–TM1 distance, as reported in Supplementary Fig. 6. **d**, Helix propensity for GCG-NH_2_ and **G3** bound to GCGR during MD simulations (realized using the MD enhanced sampling protocol; 3 independent replicas; Methods). Red arrows and asterisks on residue labels (*x* axis) indicate residues 4, 16, 20 and 24 of GCG-NH_2_ or **G3**. The α-helix propensities are expressed as mean ± s.d. of 9 (**a**) or 3 (**d**) MD replicas.

To address this methodological bias, we developed an enhanced sampling MD protocol to explore the partially unfolded conformations of glucagon and **G3** (C-terminal amide forms), with the input of simulated energy. This well-tempered metadynamics^59^ approach seeded low-height potential-energy Gaussian curves along a collective variable that defines the helical content of the peptides, favouring exploration of the unfolded states of glucagon and **G3** over time. Classic MD simulations (that is, without any energy input), started from unfolded states of GCG-NH_2_ and **G3** extracted from the enhanced sampling trajectories (three conformations for either GCG-NH_2_ or **G3**; Supplementary Fig. 5), suggested an overall lower α-helical propensity (Fig. 3a) compared to the MD simulations initiated from full-helix conformations (Supplementary Fig. 4). **G3** displayed a lower population of the helical conformation, most pronounced for the segment Ala19–Val23 (the segment in which Gln20 is replaced with d-Gln), relative to glucagon (Fig. 3a). These results are qualitatively in line with CD results indicating that the incorporation of five d-residues in **G3** destabilizes the α-helix relative to the homochiral peptide GCG-NH_2_ (Fig. 1c) and could in part explain the lower potency of **G3** at stimulating cAMP production, β-arrestin-2 recruitment or GCGR internalization relative to GCG-NH_2_ and **G1**–**G2** (Tables 1 and 2) (**G1** and **G2** contain l-Gln in position 20).

Classic MD simulations of the receptor-bound states of glucagon and **G3** (Supplementary Video 1; C-terminal amide forms) diverged most clearly near the N termini, which were embedded in the receptor TMD core. Glucagon was helical along the entire length, consistent with cryo-EM data for the receptor–hormone complex^19^, whereas the N-terminal portion of **G3** diverged from the α-helical conformation displayed by the remainder of the heterochiral peptide (Fig. 3b,c, Supplementary Fig. 3 and Supplementary Table 1). This N-terminal flexibility can be attributed to the replacement of native Gly4 with d-Ala in **G3**. Comparable N-terminal flexibility was detected via cryo-EM for the Gly4→d-Ala variant of exendin-4 bound to the GLP-1R^9^.

Contacts within the bound peptides and between peptide and receptor were altered in the C-terminal portion of **G3** relative to glucagon (Supplementary Tables 2 and 3). In the **G3** complex, the side chains of d-Gln20 and d-Gln24 formed persistent hydrogen bonds with one another (Supplementary Table 2), which seemed to diminish interactions with the side chains of receptor residues Gln122^ECD^ and Gln131^1.29^ (the latter in transmembrane helix 1 (TM1)) relative to the glucagon complex (Supplementary Table 3). Overall, heterochiral peptide **G3** showed relatively more engagement with the receptor core, and glucagon showed relatively more engagement with extracellular loops, especially extracellular loop 3 (ECL3) (Supplementary Fig. 3b). The most notable difference in the GCGR bound to these two peptides involved TM1, which was relatively straight in the complex with glucagon but bent towards the peptide in the complex with **G3** (Fig. 3b,c); this bend resulted in a shorter peptide–TM1 distance by ~1 Å (measured between d-Ser16 of **G3** and Gln131^1.29^ of the receptor relative to the Ser16–Gln131^1.29^ distance for glucagon) (Supplementary Fig. 6).

The computed binding energies suggested that the GCGR–**G3** complex is less stable than the GCGR–GCG–NH_2_ complex (Supplementary Table 4). This conclusion is consistent with the common view, based on cryo-EM data^17–23^, that peptide agonists of the GCGR and of related class B1 GPCRs are fully α-helical in the receptor-bound state, as well as the evidence that l-to-d replacement decreases α-helix stability.

We sought to test this computational prediction about relative stabilities among peptide–GCGR complexes through experiments. Agonist binding to related receptors, GLP-1R and GLP-2R, has been evaluated via BRET measurements involving engineered receptors that have a nanoluciferase (NLuc) module fused to the N terminus^9,35^. Our efforts to develop an analogous BRET-based binding assay for the GCGR were unsuccessful. We therefore used an alternative strategy to test the computational affinity prediction, by comparing the effects of a GCGR antagonist on the abilities of glucagon and **G1**–**G3** to stimulate cAMP production.

Extended Data Table 1 shows EC_50_ values for the stimulation of cAMP production by glucagon or **G1**–**G3** in the presence of a truncated peptide that serves as an antagonist of GCGR activation. The antagonist lacks the first five residues of glucagon and features Glu at position 4, which corresponds to Asp9 in glucagon itself^60^. This truncated peptide occupies the orthosteric site without stimulating cAMP production. As expected, the EC_50_ value was higher for each agonist in the presence of 100 nM antagonist (Extended Data Table 1, Supplementary Fig. 7 and Supplementary Table 6) relative to the absence of the antagonist (Table 1); that is, a higher agonist concentration was required to achieve 50% of maximum cAMP production in the presence of 100 nM antagonist than in the absence of antagonist. Extended Data Table 1 shows ΔEC_50_ (EC_50_(with antagonist)/EC_50_(without antagonist)) for each agonist. For glucagon (GCG-NH_2_), ΔEC_50_ is 10, but for the three heterochiral analogues **G1**–**G3**, ΔEC_50_ ranges between 36 and 42. These data show that the antagonist has a larger effect on heterochiral agonist potency relative to the effect on GCG-NH_2_ potency, which is consistent with the conclusion that heterochiral agonists **G1**–**G3** bind less strongly than glucagon to the GCGR.

Additional simulations of the receptor-bound states of glucagon and **G3** (C-terminal amide forms) were conducted to compensate for the force field bias towards α-helical conformations^57,58^ and to explore less-accessible conformations of glucagon and **G3** bound to the GCGR, using the same enhanced sampling protocol employed for the unbound peptides (Supplementary Fig. 5). Under such simulated conditions, **G3** displayed lower α-helical propensity than GCG-NH_2_ along most of its length (residues 8–28) (Fig. 3d and Supplementary Video 2). The N-terminal segment of **G3** (residues 2–7), however, displayed a higher helical propensity relative to the corresponding segment of GCG-NH_2_, despite the Gly4→d-Ala replacement in **G3**. The generally lower helical propensity of **G3** is consistent with the helix-destabilizing effects of the d-residues distributed along the sequence of this heterochiral peptide. **G3** partially dissociated from the receptor in one replica (final part of Supplementary Video 2), which is qualitatively consistent with the greater sensitivity of EC_50_ to the presence of the GCGR antagonist displayed by heterochiral **G3** compared to homochiral GCG-NH_2_. The increased conformational flexibility of GCG-NH_2_ and **G3** in the enhanced sampling simulations correlated with increased flexibility of the receptor in these simulations compared to the previous MD simulations involving fully helical peptides (Supplementary Fig. 9a–d). The receptor structural elements most influenced by the enhanced flexibility of the bound agonist were the ECD and the subdomain formed by intracellular loop 3 (ICL3), transmembrane helix 6 (TM6) and extracellular loop 3 (ECL3) (Supplementary Fig. 9e).

These computational results suggest that GCG-NH_2_ and **G3** in complex with the GCGR might have a different propensity to populate partially unfolded conformers, with the heterochiral analogue being more prone to adopt non-helical conformations while bound to the receptor. Flexibility displayed by the agonists in partially unfolded states could be transferred to the receptor, altering the conformational landscape and engagement with intracellular effectors such as G_s_ and β-arrestin-2.

### Proteolytic susceptibility of glucagon and G3

l-to-d replacement hinders the enzymatic cleavage of nearby peptide bonds^61,62^. We analysed the proteolytic susceptibility of heterochiral glucagon analogue **G3**, which contains five l-to-d replacements, and glucagon itself. This study involved two forms of each peptide, one with a C-terminal acid (as in native glucagon) and the other with a C-terminal amide. Two conditions were used—human serum and proteinase K (an aggressive fungal protease that is often used to assess proteolytic susceptibility)^63–65^.

Extended Data Table 2 provides half-life data for the four peptides under each condition. In human serum, the difference between C-terminal forms (acid versus amide) did not significantly affect the half-life for either glucagon or heterochiral analogue **G3**. As anticipated, the l-to-d substitutions in **G3** led to slower degradation, although the effect was modest, approximately threefold for each C-terminal form (Extended Data Table 2 and Supplementary Fig. 10). Larger variations were observed in the presence of proteinase K. The C-terminal amide form of glucagon displayed a moderately longer half-life relative to the C-terminal acid form, which raises the possibility that the proteinase K was contaminated with a C-terminal exopeptidase. Heterochiral analogue **G3**–**OH** was substantially protected from proteinase K action relative to glucagon: the half-life was extended 28-fold. The C-terminal amide **G3** displayed an approximately threefold longer half-life relative to the C-terminal amide form of glucagon (Extended Data Table 2 and Supplementary Fig. 11).

### Potent heterochiral PTH(1–34) analogues

We turned to the PTH1R to ask whether multiple l-to-d substitutions would be tolerated in a polypeptide agonist unrelated to glucagon. Human parathyroid hormone (PTH) comprises 84 residues, but the fragment corresponding to the first 34 residues displays potent activity at the PTH1R. PTH(1–34) is used as teriparatide to treat osteoporosis^66^. PTH(1–34) is fully α-helical when bound to the PTH1R^23,67–69^. Based on our findings with heterochiral analogues of glucagon, we focused on positions in PTH(1–34) that are solvent-facing in the cryo-EM structures; these positions occur in the C-terminal segment of the all-l peptide (Met18, Glu22, Lys26, Asp30; Phe34 of PTH(1–34) could not be resolved in the cryo-EM structure, PDB 8FLQ; Supplementary Fig. 2b).

**Fig. 4:**
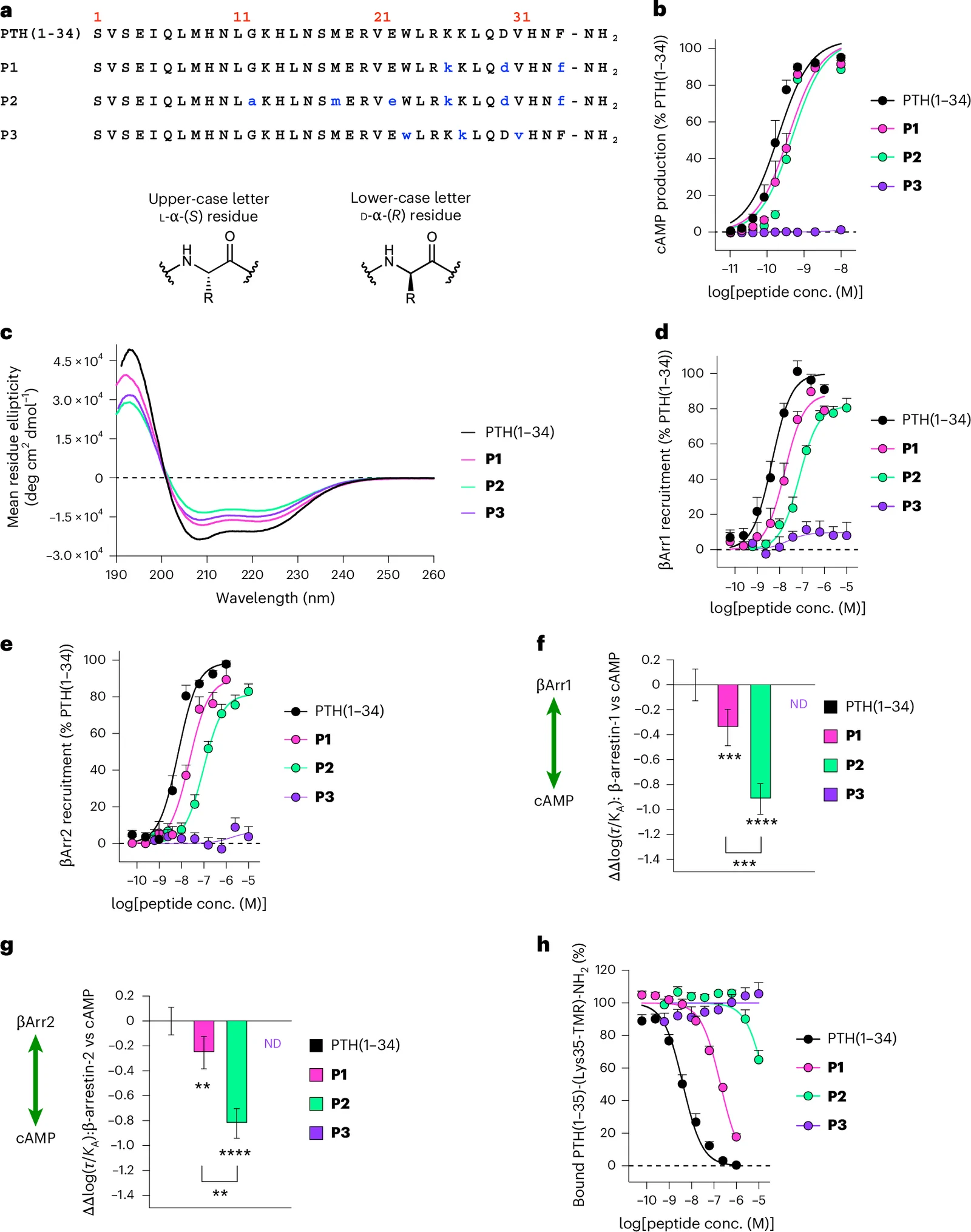
Heterochiral PTH(1–34) analogues display native-like cAMP potency and signal bias at the PTH1R. **a**, Sequences of PTH(1–34) and heterochiral analogues **P1**–**P3**. Sites of d-residue replacements are indicated by lower-case letters and highlighted in blue. **b**, Activation of the PTH1R by PTH(1–34) and its heterochiral analogues. The assay monitored levels of cAMP production in HEK293 cells stably expressing the PTH1R and the cAMP-sensing protein GloSensor. Data points represent the average of six independent experiments. **c**, Circular dichroism data obtained in PBS, pH 7.4, containing 20 vol% TFE. **d**,**e**, Recruitment of β-arrestin-1 (**d**) or -2 (**e**) to the PTH1R. Assays were performed in CHO-FlpIn cells stably transfected with PTH1R-RLuc8 and β-arrestin-1-Venus or β-arrestin-2-Venus; these assays measured the ability of β-arrestin-1 or -2 to be recruited to the PTH1R via BRET. Data represent the mean of ≥5 (β-arrestin-1) (*n* = 7 for PTH(1–34), *n* = 5 for **P1**, *n* = 6 for **P2**, *n* = 6 for **P3**) or ≥6 (β-arrestin-2) (*n* = 10 for PTH(1–34), *n* = 6 for **P1**, *n* = 10 for **P2**, *n* = 10 for **P3**) independent experiments. **f**,**g**, β-arrestin-1 (**f**) or -2 (**g**) versus cAMP bias factors (ΔΔlog(*τ*/*K*_A_)) calculated as the differences of the differences between transduction coefficients (log(τ/*K*_A_)) calculated according to the Black–Leff operational model from the dose–response curves in **b** (six independent experiments) and **d** (≥5 independent experiments) (or **b** (six independent experiments) and **e** (≥6 independent experiments)) for analogues **P1**–**P3** relative to PTH(1–34). A negative value indicates bias towards cAMP production. **h**, Competition binding measurements by BRET in HEK293 cells expressing the NLuc-PTH1R. The peptide tracer was a tetramethylrhodamine (TMR)-labelled derivative of PTH(1–34), PTH(1–35)-(Lys35-TMR)-NH_2_. Data (as IC_50_ values) represent the average of ≥3 independent experiments (*n* = 8 for PTH(1–34), *n* = 3 for **P1**, *n* = 4 for **P2**, *n* = 4 for **P3**). Bias factor uncertainties in **f**,**g** are expressed as derived mean ± propagated s.d. **P ≤ 0.01; ***P ≤ 0.001; ****P ≤ 0.0001; one-way ANOVA followed by Dunnett test. ND, not determined. *P* values in the form of an asterisk are shown for individual peptides in **f**,**g**. All cAMP production, β-arrestin-1 or β-arrestin-2 recruitment, and NLuc-PTH1R affinity data (**b**,**d**,**e**,**h**) were normalized against PTH(1–34), and all uncertainties are expressed as s.e.m. Source data

Our design strategy provided heterochiral analogues of PTH(1–34) that are potent agonists of the PTH1R, as judged by cAMP production in cells that over-express the receptor and GloSensor (Fig. 4a). Analogue **P1**, containing l-to-d substitution at Lys26, Asp30 and Phe34, was only approximately twofold less potent relative to the all-l diastereomer (Fig. 4b and Extended Data Table 3). Analogue **P2**, which has the three substitutions in **P1** along with l-to-d substitution at Met18 and Glu22 and replacement of Gly12 with d-Ala, was indistinguishable from **P1** in terms of EC_50_ (Fig. 4b and Extended Data Table 3). The Gly12-to-d-Ala modification, alone, in PTH(1–34) does not diminish potency^70^. Heterochiral PTH(1–34) analogues **P1** and **P2** matched the maximum level of cAMP production induced by PTH(1–34). Preliminary efforts to introduce an additional site of l-to-d substitution nearer the N terminus and retain receptor potency were unsuccessful. The lack of substitution in the N-terminal portion of **P2** presumably explains the lack of protection from degradation by proteinase K relative to PTH(1–34) (Supplementary Fig. 13 and Supplementary Table 8). Nevertheless, it is noteworthy that heterochiral analogue **P2**, containing six well-distributed d-residues, is a potent agonist of the PTH1R. CD analysis in aqueous buffer containing 20 vol% TFE was consistent with the prediction that the d-residues in heterochiral peptides **P1** and **P2** should diminish α-helical propensity relative to homochiral PTH(1–34) (Fig. 4c).

l-to-d replacement at sites in PTH(1–34) that make direct contact with the receptor was deleterious to agonist activity, which is consistent with our observations in the glucagon system. Thus, analogue **P3**, with d-residues at Trp23, Lys27 and Val31, did not induce cAMP production at 10 nM, a concentration at which PTH(1–34) and analogues **P1** and **P2** reached maximum response (Fig. 4b and Extended Data Table 3). PTH(1–34) and heterochiral analogues were prepared as C-terminal amides, consistent with past studies^8,11^.

The heterochiral analogues were compared with PTH(1–34) in BRET-based assays that detect recruitment of either β-arrestin-1 or β-arrestin-2 to the PTH1R, analogous to the described assay employed for the GCGR. **P1** was modestly less effective than PTH(1–34) at recruiting each β-arrestin (higher EC_50_) (Fig. 4d,e and Extended Data Table 4). More heavily substituted analogue **P2** manifested a larger defect in β-arrestin recruitment relative to PTH(1–34), in terms of both potency (EC_50_) and efficacy (maximum response). The operational model indicated that **P1** is moderately biased away from recruitment of either β-arrestin relative to cAMP production (Fig. 4f,g) relative to PTH(1–34); heterochiral analogue **P2** displayed a more substantial bias away from β-arrestin recruitment relative to PTH(1–34). The difference in bias between **P1** and **P2** was statistically significant.

Binding of **P1**–**P3** to the PTH1R was evaluated with a BRET-based assay involving a receptor construct bearing NLuc at the N terminus^71^. This competition assay monitored the ability of each analogue to displace a fluorescently labelled derivative of PTH(1–34) (Fig. 4h). PTH(1–34) itself potently displaced the tracer, with a half-maximal inhibitory concentration (IC_50_) value of 4 nM (Extended Data Table 4). Heterochiral analogue **P1** was less effective at tracer displacement, with an ~50-fold larger IC_50_ relative to PTH(1–34). **P2**, with six l-to-d displacements, was even poorer at tracer displacement, with an ~5,000-fold larger IC_50_ relative to PTH(1–34). No tracer displacement by heterochiral analogue **P3** could be detected, which is consistent with the inability of this peptide to activate the receptor.

To gain further insight into the affinity of **P2**, we prepared a derivative containing a C-terminal Lys residue with a tetramethylrhodamine (TMR) unit attached to the side chain. The analogous derivative of PTH(1–34) was used as the tracer in the competition binding assays described above; previous direct-binding studies with PTH(1–34)-K35^TMR^ indicated that the dissociation constant (*K*_d_) of 12 nM for binding to the PTH1R^71^. PTH(1–34)-K35^TMR^ was comparable to PTH(1–34) itself as an agonist of the PTH1R^71^, and we found that **P2**-K35^TMR^ was comparable to **P2** in stimulating cAMP production (Supplementary Fig. 14 and Supplementary Table 9). Direct-binding studies with **P2**-K35^TMR^ provided *K*_d_ of 492 nM for binding to the PTH1R (Supplementary Fig. 15). Thus, this comparison indicates ~40-fold weaker binding of **P2** relative to PTH(1–34).

The decreases in PTH1R affinity of **P1** and **P2** relative to PTH(1–34) are consistent with the expectation that multiple l-to-d substitutions should diminish the stability of the α-helical conformation that has been observed in multiple peptide + PTH1R structures^23,67–69^. The expected conformational stability decline is qualitatively consistent with CD data for **P1** and **P2**. Nevertheless, both heterochiral peptides are potent stimulators of intracellular cAMP production mediated by PTH1R activation. The finding that these heterochiral PTH(1–34) analogues are potent agonists would not have been predicted based on any precedent of which we are aware.

## Conclusions

The peptides that activate the GCGR, the PTH1R or others among the class B1 GPCRs have long been thought to prefer fully α-helical conformations in the receptor-bound state, a conclusion supported by a large body of recent cryo-EM data^17–23^. This structural understanding has motivated extensive efforts to develop peptide agonists by replacing native residues with other l-α-amino-acid residues to preserve a right-handed α-helix conformation^7–16^. Some attempts to optimize agonist function have focused on enhancing α-helicity, for example, by installing side-chain crosslinks that stabilize the helical conformation^72–77^. Thus, the structural data and previous optimization efforts would seem to discourage the incorporation of subunits known to destabilize a right-handed α-helix, such as d-α-amino-acid residues.

We have shown that glucagon and PTH(1–34) analogues containing three or more l-to-d substitutions can serve as potent agonists. This unanticipated discovery points toward a large and previously unrecognized region of molecular space that is likely to contain many class B1 GPCR agonists with potentially useful activity profiles. Our finding that heterochiral agonists can display biased signalling profiles suggests that they may be useful as tools for isolating cellular outcomes among the multiple signalling pathways initiated at a given GPCR^78,79^. In addition, biased agonists have aroused considerable translational interest, because signal bias might circumvent deleterious on-target side effects from pathways that do not contribute to therapeutic efficacy^80,81^. The reduced affinity of heterochiral agonists for the PTH1R or GCGR, relative to the homochiral agonists, suggests that this class of analogues could be useful for evaluating the hypothesis that the physiological impact of GPCR activation can be modulated by tuning receptor residence time^82,83^.

The findings disclosed here challenge the assumption that maintaining or enhancing α-helical stability is a useful guideline for the design of class B1 GPCR agonists. Indeed, our results support an emerging view that emphasizes the role of agonist dynamics in signal transduction via GPCRs^9,84^. The static helical conformations observed almost universally among native agonist peptides bound to class B1 receptors may not fully elucidate the physical mechanisms necessary for transmission of information through the receptor to the cell interior. Heterochiral hormone analogues of the type introduced here should be powerful tools for addressing this hypothesis.

## Methods

Lists of reagents and resources used in this study are provided in Supplementary Table 10.

### Peptide synthesis, purification and characterization

The peptides were synthesized on a CEM Liberty Blue synthesizer using low-loading ProTide Rink amide resin or pre-loaded Wang resin at 50-μmol scale. Activated amino-acid solution was prepared by combining 1.25 ml of 0.2 M Fmoc-protected amino acid with 0.5 ml of 0.5 M *N*,*N*′-diisopropylcarbodiimide (DIC) and 0.5 ml of 1 M Oxyma, in biotech-grade dimethylformamide (DMF). Couplings were performed at 90 °C for 2 min, except for couplings with Fmoc-His(Trt)-OH, which were performed at 50 °C for 10 min. Each residue was double-coupled. Deprotections were performed with 20% (vol/vol) piperidine and 0.1 M Oxyma in ACS-grade DMF at 90 °C for 2 min. Details for the synthesis of **P2** labelled with TMR (**P2**-(Lys35-TMR)) are provided in the Supplementary Methods.

Resin cleavage was achieved with 8 ml of 92.5% trifluoroacetic acid (TFA; vol/vol), 5% thioanisole (vol/vol) and 2.5% 1,2-ethanedithiol (vol/vol) for glucagon analogues or 5 ml of 90% TFA (vol/vol), 5% thioanisole (vol/vol), 3% 1,2-ethanedithiol (vol/vol) and 2% anisole (vol/vol) for PTH(1–34) analogues at room temperature for 4 h. Crude peptide was precipitated with 35 ml of cold diethyl ether, then pelleted by centrifugation at 1,500*g* for 5 min. The supernatant was discarded, and the pellet was washed with 35 ml of cold diethyl ether and pelleted again. The crude peptide pellet was dried under a N_2_ stream. Preparative reverse-phase high-performance liquid chromatography (HPLC) was performed on a Waters modular HPLC system or an Agilent 1260 Infinity II HPLC system fitted with a Waters or Supelco C18 column and eluted with gradients between 0.1% TFA (vol/vol) in Nanopure water and 0.1% TFA (vol/vol) in HPLC-grade acetonitrile. The purity of peptides was assessed using a Waters Acquity H-Class UPLC instrument fitted with an Acquity UPLC Peptide BEH C18 column (1.7 μm, 2.1 × 100 mm or 2.1 × 150 mm) for glucagon analogues or an Acquity UPLC Peptide CSH C18 column (1.7 μm, 2.1 × 100 mm) for PTH(1–34) analogues. For peptide characterization, matrix-assisted laser desorption/ionization time-of-flight mass spectrometry (MALDI-TOF-MS) data were acquired on a Bruker microflex MALDI-TOF mass spectrometer or a Bruker ultraflex MALDI-TOF mass spectrometer with α-cyano-4-hydroxycinnamic acid (CHCA) as the matrix. Additionally, electrospray ionization–mass spectrometry (ESI-MS) data were collected on a Bruker Impact II (QTOF) mass spectrometer with an UltiMate 3000 HPLC fitted with an Agilent PLRP-S (3 µM, 2.1 × 50 mm, 300 Å) column. LCMS (ESI-MS) samples were eluted with 45–95% B (where A = 0.1% (vol/vol) formic acid/water; B = 0.1% (vol/vol) formic acid/acetonitrile (MeCN)) at 0.3 ml min^−1^.

### Cell culture

HEK293 cells (ATCC cat. no. CRL-1573) stably expressing the GloSensor cAMP reporter (plasmid p22f, Promega; HEK293GS22) were cultured in tissue-treated T75 culture flasks in Dulbecco’s modified Eagle medium (DMEM) supplemented with 10% (vol/vol) fetal bovine serum (FBS) and 1× penicillin/streptomycin at 37 °C in 5% CO_2_. HEK293FT cells were cultured under the same conditions, but the medium was supplemented with 2 mM l-glutamine, 1 mM sodium pyruvate and 0.1 mM MEM non-essential amino acids. Cells were split 1:20 or 1:30 every 5–7 days when they reached full confluence using 0.05% trypsin–ethylenediaminetetraacetic acid (EDTA) in Dulbecco’s phosphate-buffered saline (DPBS).

HEK293 cells stably expressing the WT-hPTH1R and the GloSensor construct (GP2.3) were cultured in T25 flasks in DMEM (with d-glucose but lacking l-glutamine and sodium pyruvate) supplemented with 10% (vol/vol) FBS and incubated at 37 °C with 5% CO_2_. CHO-FlpIn cells stably transfected with PTH1R-RLuc8 and β-arrestin-1-Venus or β-arrestin-2-Venus were cultured under the same conditions. 0.05% trypsin–EDTA in DPBS was used to detach cells for passaging every 5–7 days once the cells reached confluence.

### cAMP production assays

For evaluation of cAMP production at the GCGR, the protocol was adapted from that described previously^10^ with slight modifications. HEK293 cells stably expressing the GloSensor construct were grown in a 10-cm tissue culture-treated dish until they reached 80% confluency. A transfection mixture of 1 ml of Opti-MEM with 10 μg of GCGR-Flag plasmid and 30 μl of Fugene HD transfection reagent was incubated for 20 min then added to the dish. The next day, the cells were collected with 0.05% EDTA/trypsin and resuspended in DMEM + 10% FBS medium for plating in a white, flat-bottom, tissue culture-treated 96-well plate (~50,000 cells well^−1^). The next day, the medium was gently flicked from the plate and immediately replaced with 90 μl of DPBS containing 0.5 mM d-luciferin substrate. The cells were incubated for 30 min at room temperature, after which 10 μl of various peptide doses (10× final concentration) were added for a final volume of 100 μl. The plate was read on a BioTek Synergy Neo2 plate reader using a luminescence cube. The luminescence values observed between 15 and 20 min were used to generate concentration dose–response curves.

For cAMP production at the PTH1R, GP2.3 cells were seeded onto a white 96-well plate at ~50,000 cells well^−1^. After incubating at 37 °C for 24 h, the medium was aspirated, and 90 μl of Gibco CO_2_ independent medium supplemented with 0.5 mM d-luciferin was added to each well. The plate was incubated at room temperature for 20 min. To the cells were added 10 μl of different concentrations of peptide dilutions in CO_2_ independent medium, and the luminescence was recorded for 30 min using a BioTek Synergy Neo2 plate reader. The luminescence readings from 14 to 16 min were used to generate concentration–response curves.

EC_50_ and maximal response (% Max) values were determined by normalizing, averaging and then fitting data to three-parameter sigmoidal curves in GraphPad Prism 10. The bottom of the curves was constrained to 0%. Normalization was performed for individual experiments, with 100% representing the top of the curve for GCG-NH_2_ or PTH(1–34), and 0% representing the luminescence value in the absence of peptide.

### GCGR cAMP production assays with the GCGR antagonist

For GCGR cAMP production assays, the protocol was adapted from that described previously^60^. HEK293 cells stably expressing the GloSensor construct were transfected with GCGR-Flag plasmid and seeded in white tissue culture-treated 96-well plates as already described for the cAMP assays. On the day of the assay, the medium was aspirated and replaced with 90 μl of Gibco CO_2_ independent medium supplemented with 500 μM d-luciferin and 100 nM GCGR antagonist, peptide 11, as previously reported^60^. The plate was incubated at room temperature for 20 min, then read on a BioTek Synergy Neo2 plate reader for 10 min to record the baseline luminescence. We added 10 μl of various peptide doses (10× final concentration), and the luminescence was recorded for 30 min. Luminescence values between 15 and 20 min were used to generate concentration dose–response curves. Reported EC_50_ and % Max values were a result of normalizing, averaging and then fitting data to three-parameter sigmoidal curves in GraphPad Prism 10. The bottom of the curves was constrained to 0%. Normalization was performed, with 100% representing the top of the curve for GCG-NH_2_ (no GCGR antagonist) for individual experiments, and 0% representing the baseline luminescence value after incubation with ‘peptide 11 GCGR antagonist’.

### β-arrestin recruitment assays

For evaluating β-arrestin-2 recruitment to the GCGR, we adapted a previous protocol^10^, with slight modifications. HEK293FT cells were grown on a 10-cm tissue culture-treated dish until reaching 80% confluency. The culture medium was aspirated and replaced with 10 ml of fresh DMEM + 10% FBS. A transfection mixture consisting of 1 ml of Opti-MEM with 30 μl of Transporter 5 (PEI MAX) transfection reagent, 14 μg of GFP^2^-β-arrestin-2 (R393E, R395E) plasmid and 130 ng of GCGR-RLuc8 plasmid was incubated for 20 min at room temperature before being pipetted into the cell dish. The next day, the cells were collected from the dish with 0.05% EDTA/trypsin and resuspended in DMEM + 10% FBS medium for plating in a white, flat-bottom, tissue culture-treated 96-well plate (~100,000 cells per well). The next day, the medium was gently pipetted out, after which the cells were washed once with 90 μl of DPBS + glucose before being incubated in 90 μl of fresh DPBS + glucose. The cells were incubated at 37 °C with 5% CO_2_ for 1 h. After this, DeepBlueC substrate was added to a final concentration of 5 μM. After 5 min, 10 μl of peptide dilutions were added to the wells, and the plate was read on a BioTek SynegyNeo2 plate reader using the 400/510 cube. Dose–response curves were generated with Lum510/Lum400 values taken between 5 and 15 min after the peptides were added.

For β-arrestin-1 and β-arrestin-2 recruitment at the PTH1R, a previous protoco^85^ was used, with slight modifications. CHO-FlpIn cells stably expressing PTH1R-RLuc8 and β-arrestin-1-Venus or β-arrestin-2-Venus were seeded onto a white-bottomed 96-well plate at 100,000 cells well^−1^. After incubating at 37 °C for 24 h, the medium was aspirated, and each well was rinsed with 50 μl of DPBS. A 90-μl volume of DPBS supplemented with 5 μM coelenterazine-H was added, and the plate was incubated at 37 °C for 10 min. To the cells was added 10 μl of peptide solution in DPBS at various concentrations, and the luminescence from 480 nm and 530 nm was measured for 30 min using a BioTek Synergy Neo2 plate reader. The ratio of luminescence from the 530-nm channel over that from the 480-nm channel with a 1,000× multiplier was calculated for vehicle wells and subtracted from the ratio from agonist-treated wells to obtain BRET values. BRET values from 12 to 14 min were used to generate concentration–response curves. EC_50_ and % Max values were analysed by normalizing, averaging and then fitting data to three-parameter sigmoidal curves in GraphPad Prism 10. The bottom of the curves was constrained to 0%. Normalization was performed for individual experiments, with 100% representing the top of the curve for PTH(1–34), and 0% representing the BRET value in the absence of peptide.

### Total GCGR internalization assays

To measure total GCGR internalization in HEK293 cells, we used the PathHunter eXpress assay by Eurofins Discovery (DiscoverX Products Company; cat. no. 98-0820E1CP0M). The manufacturer’s protocol was followed, with one exception: 5,000 cells well^−1^ were used instead of the recommended 10,000 cells well^−1^.

### Signalling bias analysis

The protocol for calculating bias factors is provided in the Supplementary Methods.

### Proteolysis with proteinase K

Protocols for proteolysis with proteinase K and in human serum are described in the Supplementary Methods.

### NLuc-PTH1R competition binding

For NLuc-PTH1R competition binding, the procedure described previously^71^ was used, with slight modifications. HEK293 cells stably expressing NLuc-PTH1R were seeded on a white-bottomed 96-well plate at 10,000 cells well^−1^. After incubation at 37 °C for 24 h, the medium was aspirated, and each well was rinsed with 50 μl of DPBS. An 80-μl volume of DPBS supplemented with 1 mM CaCl_2_, 0.5 mM MgCl_2_, 0.1% bovine serum albumin (BSA) and 0.02% NaN_3_ (to halt cellular metabolism) was added, then the plate was incubated at room temperature for 30–60 min. To the cells was added 10 μl of peptide solution in DPBS at various concentrations, along with 10 μl of 300 nM PTH(1–35) (Lys35-tetramethylrhodamine)-NH_2_ (final concentration of 30 nM) as a tracer. The plate was incubated at room temperature for 1 h. After equilibration, 10 μl of 50 μM coelenterazine-H substrate was added, and the luminescence at 450 nm and 610 nm was measured using a BioTek Synergy Neo2 plate reader. The mBRET values were calculated as the ratio of the luminescence from the 610-nm channel over the 450-nm channel with a 1,000× multiplier, with the mBRET value from vehicle wells subtracted. Reported IC_50_ values were a result of normalizing, averaging and fitting data to three-parameter sigmoidal curves in GraphPad Prism 10. The top and bottom of the curves were constrained to 100% and 0%, respectively. Normalization for individual experiments was performed, with 100% and 0% representing the top and bottom of the curve, respectively, for PTH(1–34).

### NLuc-PTH1R kinetic direct binding for P2

The protocol is provided in the Supplementary Methods.

### GCG-NH_2_–GCGR and G3–GCGR equilibrium simulations

GCG-NH_2_ in complex with its receptor GCGR and G_s_ (PDB 6LMK) was prepared by removing G_s_. **G3** in complex with GCGR was prepared by superimposition onto GCG-NH_2_. The resulting GCG-NH_2_–GCGR and **G3**–GCGR systems were prepared for MD simulations with the CHARMM36 force field^86^, employing in-house Python htmd^87^ and TCL (tool command language) scripts. Hydrogen atoms were added at a simulated pH of 7.0 by means of the pdb2pqr^88^ and propka^89^ software, and the protonation of titratable side chains was checked by visual inspection. Each system was superimposed on the GCGR coordinates retrieved from the OPM database^90^ to correctly orient the receptor before inserting^91^ in a rectangular 90 Å × 90 Å 1-palmitoyl-2-oleyl-*sn*-glycerol-3-phosphocholine (POPC) bilayer (previously built using the VMD Membrane Builder Plugin 1.1; http://www.ks.uiuc.edu/Research/vmd/plugins/membrane/), removing the lipid molecules overlapping the receptor TMs bundle. TIP3P water molecules^92^ were added to the simulation box (90 Å × 90 Å × 140 Å) using the VMD Solvate Plugin 1.5 (http://www.ks.uiuc.edu/Research/vmd/plugins/solvate/). Overall charge neutrality was reached by adding Na^+^/Cl^−^ counter ions (final ionic concentration of 0.150 M) using the VMD Autoionize Plugin 1.3 (http://www.ks.uiuc.edu/Research/vmd/plugins/autoionize/).

Equilibration and MD productive simulations were computed using ACEMD^93^. Isothermal–isobaric conditions (Monte Carlo barostat^94^ with a target pressure of 1 atm; Langevin thermostat^95^ with a target temperature 300 K and damping of 1 ps^−1^) were used to equilibrate the systems through a multi-stage procedure (integration time step of 2 fs). Clashes between lipid atoms were first reduced via 1,500 conjugate-gradient minimization steps, then a positional constraint of 1 kcal mol^−1^ Å^−2^ lipid phosphorus atoms, protein Cα carbon and all the other protein heavy atoms were applied and gradually released over 4, 120 and 100 ns, respectively, before a further 30 ns without any restraints for a total of 150 ns.

Productive trajectories (three 2-μs-long replicas for each complex, total of 6 μs) were computed with an integration time step of 4 fs in the canonical ensemble (NVT) at 300 K, using a thermostat damping of 0.1 ps^−1^ and the M-SHAKE algorithm^96^ to constrain the bond lengths involving hydrogen atoms. The cutoff distance for electrostatic interactions was set at 9 Å, with a switching function applied beyond 7.5 Å. Long-range Coulomb interactions were handled using the particle mesh Ewald summation method (PME)^97^ by setting the mesh spacing to 1.0 Å. For the analysis, the first half of each replica was discarded as part of the equilibration.

### Enhanced sampling simulation protocol for GCG-NH_2_ and G3 in complex with GCGR

The GCG-NH_2_–GCGR and **G3**–GCGR systems, prepared as reported above (see ‘GCG-NH_2_–GCGR and G3–GCGR equilibrium simulations’ section), were subjected to three independent replicas of 1.5 μs (4.5 μs in total) of well-tempered Metadynamics^59^. The collective variable (CV) ALPHARMSD^98^ implemented in Plumed 2.7^99^ was used to sample the helicity of peptide residues 6–29 (residues 1–5 were not considered because they are inherently more flexible than the rest of the agonist during equilibrium MD simulations). This CV quantifies the number of segments formed by six consecutive residues within the peptide. Each of these segments is compared to a reference α-helix by calculating the root-mean-square deviation (RMSD) between the observed configuration and the reference helical structure. This approach provides a quantitative measure of how closely each segment resembles an ideal α-helix, enabling structural analysis of the helical content across the peptides. The reference for the ALPHARMSD computation was the initial full-helix conformations of GCG-NH_2_ and **G3**, with the OPTIMAL alignment method set. Gaussian potential-energy functions were added to the CV every 2 ps, with functions shape-defined by SIGMA = 0.05 and initial HEIGHT = 0.418 kJ mol^−1^; the BIASFACTOR was 17.89, with a temperature of 310 K.

### GCG-NH_2_ and G3 equilibrium simulations in water

The C-terminal amidated form of GCG (GCG-NH_2_) and **G3** were inserted in two separate simulation boxes with a padding of at least 15 Å using the VMD Solvate Plugin 1.5 (http://www.ks.uiuc.edu/Research/vmd/plugins/solvate/). Overall charge neutrality was reached by adding Na^+^/Cl^−^ counter ions (final ionic concentration of 0.150 M), using the VMD Autoionize Plugin 1.3 (http://www.ks.uiuc.edu/Research/vmd/plugins/autoionize/). The resulting systems were minimized for 500 steps of conjugate gradient and equilibrated in NPT conditions with the CHARMM36 force field^86^ (Monte Carlo barostat^94^ with a target pressure 1 atm; Langevin thermostat^95^ with a target temperature of 300 K and damping of 1 ps^−1^) for 3 ns using ACEMD^93^. A positional constraint of 1 kcal mol^−1^ Å^−2^ on protein Cα carbon and all the other protein heavy atoms was applied and gradually released over 3 and 1 ns, respectively. All the other MD settings were the same as reported above (see ‘GCG-NH_2_–GCGR and G3–GCGR equilibrium simulations’ section). After equilibration, three replicas of 10 μs each, for both GCG-NH_2_ and **G3**, were collected, for a total of 30 μs for each peptide in water.

### Enhanced sampling simulation protocol for GCG-NH_2_ and G3 in water

GCG-NH_2_ and **G3**, prepared in water as reported above (see ‘GCG-NH_2_ and G3 equilibrium simulation in water’ section) were subjected to three independent replicas of 3 μs (9 μs in total) of well-tempered Metadynamics. The CV ALPHARMSD implemented in Plumed 2.7 was used to sample the helicity of the peptides (residues 1–29) using the initial full-helix conformations of GCG-NH_2_ and **G3** as reference. For each of the three enhanced sampling replicas, the frame with the lowest ALPHARMSD values (that is, unfolded states, Supplementary Fig. 5) was extracted and used to seed three classic (equilibrium, not enhanced) MD simulations of 3 μs from each, for a total of 27 μs for both GCG-NH_2_ and **G3** (3 μs × 3 replicas seeded from three initial enhanced sampling replicas).

### MD analysis

Hydrogen bonds were quantified using the GetContacts analysis tool (https://getcontacts.github.io/) and quantified as the percentage of frames (over all the frames obtained by merging the different replicas) in which they were present. The distance between the peptide and GCGR TM1 was computed at the Cα level of Ser16 (GCG-NH_2_) or (d-)Ser16 (**G3**) and Q131^1.29^ using VMD^100^.

The DSSP (Dictionary of Secondary Structure of Proteins) analysis^101^ was performed using the AmberTools20 suite (The Amber Molecular Dynamics Package; http://ambermd.org/). The MMPBSA.py^102^ script from AmberTools20 was used to compute molecular mechanics energies combined with the generalized Born and surface area continuum solvation (MM/GBSA) method or the molecular mechanics Poisson–Boltzmann surface area (MM/PBSA) approach, after transforming the CHARMM psf topology files to an Amber prmtop format using ParmEd (documentation at http://parmed.github.io/ParmEd/html/index.html). VMD 1.9.2^100^ was used for root mean square fluctuation (RMSF) and Rainbow analyses^103^, the latter performed by superimposing on Cα atoms of GCGR every frame and computing the RMSD of each GCGR residue’s Cα. Supplementary Videos were produced with VMD and FFmpeg (https://ffmpeg.org/). Molecular graphics images were produced using UCSF Chimera^104^ (v1.14).

### Numbering system

Throughout this Article, the Wootten residues numbering system for class B1 GPCRs^105^ has been adopted.

### Reporting summary

Further information on research design is available in the Nature Portfolio Reporting Summary linked to this Article.

## Online content

Any methods, additional references, Nature Portfolio reporting summaries, source data, extended data, supplementary information, acknowledgements, peer review information; details of author contributions and competing interests; and statements of data and code availability are available at https://doi.org/10.1038/s41557-026-02182-x.

## Supplementary information


Supplementary InformationSupplementary Figs. 1–15, Supplementary Tables 1–10, Supplementary Note, Supplementary References.
Reporting Summary
Supplementary Video 1Comparison between GCG-NH_2_ and **G3** (dark grey ribbon) in complex with GCGR (white ribbon) during classic MD simulations. Data from three MD replicas; **G3** d-amino acids are in bold, green stick representation. GCGR residues within 3.5 Å are shown in thin, stick representation.
Supplementary Video 2Comparison between GCG-NH_2_ and **G3** (dark grey ribbon) in complex with GCGR (white ribbon) during enhanced MD simulations. Data from three MD replicas; **G3** d-amino acids are in bold, green stick representation. GCGR residues within 3.5 Å are shown in thin, stick representation.
Supplementary Video 3Principal component analysis of GCG-NH_2_ and **G3** in complex with GCGR during classic (top row) or enhanced (bottom row) MD simulations. The structural transitions associated to the first component, computed on the Cα atoms, are shown for each system.
Source Data for Supplementary Figures and TablesSource Data for Supplementary Figures and Tables.


## Source data


Source Data Fig. 1. Source Data Table 1Statistical source data
Source Data Fig. 2. Source Data Table 2Statistical source data
Source Data Fig. 4. Source Data Extended Data Table 3. Source Data Extended Data Table 4Statistical source data
Source Data Extended Data Fig./Table 1Statistical source data
Source Data Extended Data Fig./Table 2Statistical source data


## Figures and Tables

**Extended Data Table 1:**
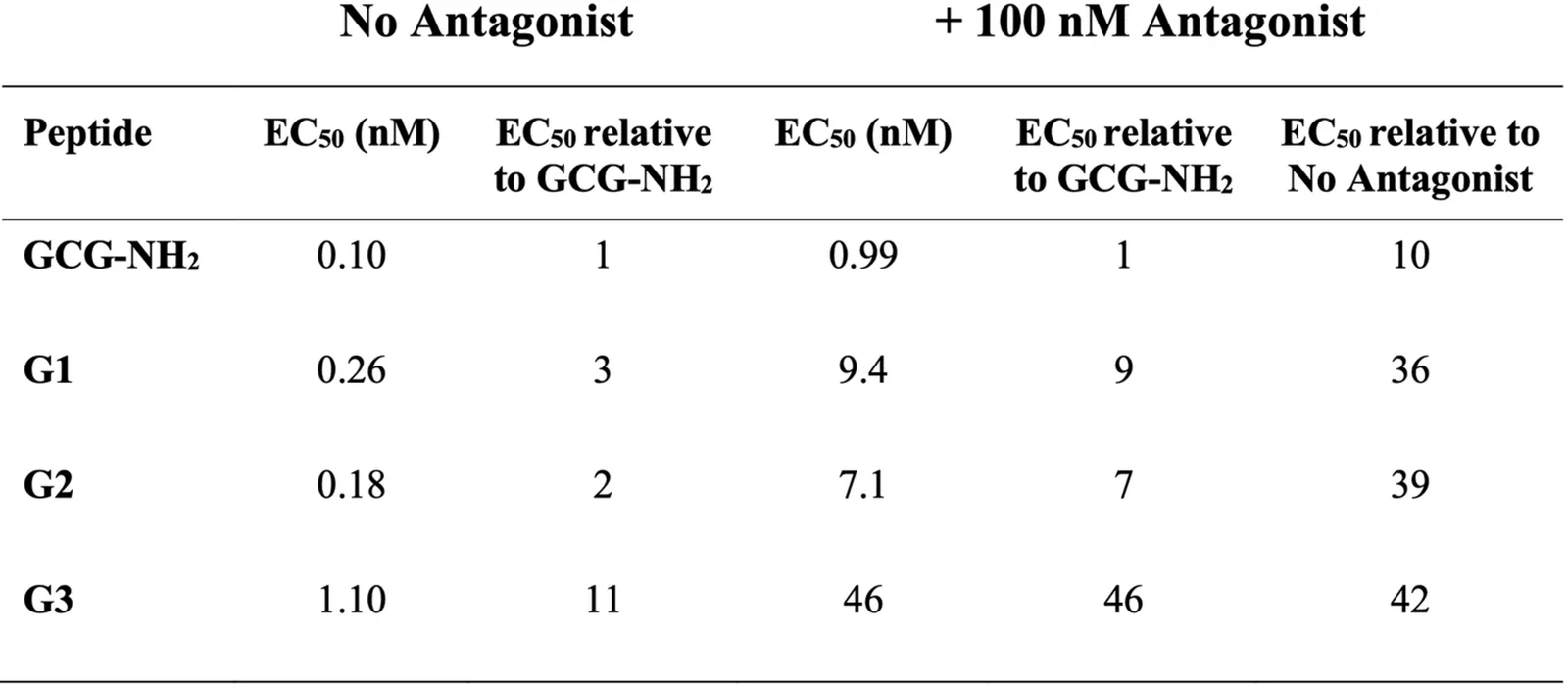
Comparison of cAMP production potency values for GCG-NH2 and heterochiral analogues G1–G3 without the GCGR antagonist or with the +100 nM antagonist

**Left portion of the table**, for the ‘No Antagonist’ condition, EC_50_ and EC_50_ relative values for GCG-NH_2_ and G1–G3 were duplicated from Table 1 (computed from dose–response curves shown in Fig. 1b). **Right portion of the table**, for the ‘+ 100 nM Antagonist’ condition, EC_50_ and EC_50_ relative values for GCG-NH_2_ and **G1**-**G3** were duplicated from Supplementary Table 6 (computed from dose–response curves found in Supplementary Fig. 7).

Source data

**Extended Data Table 2:**
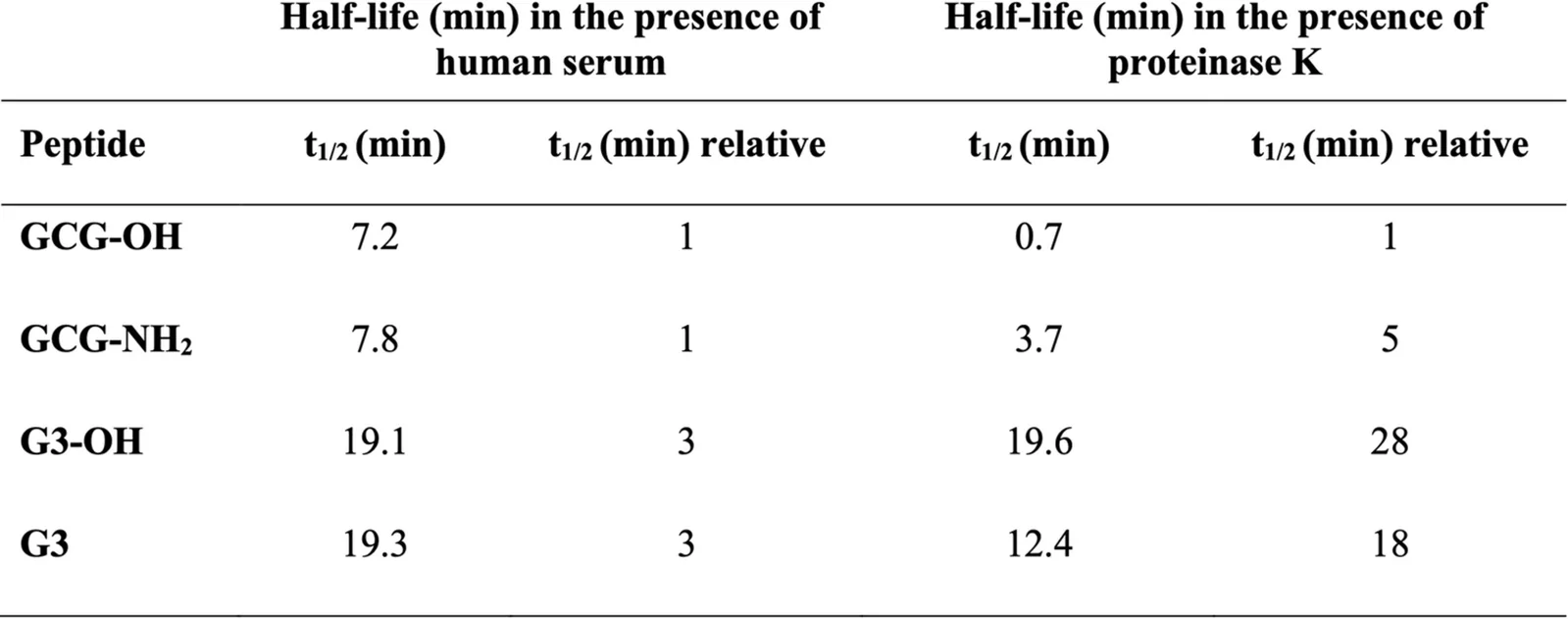
Half-life (min) in the presence of human serum or proteinase K for glucagon and analogues

Half-life values for glucagon and analogues in the presence of human serum (**left portion of the table**) or proteinase K (**right portion of the table**), as determined from one-phase decay curves plotted using data obtained from 3 independent experiments. t_1/2_ (min) relative indicates the half-life normalized to GCG-OH (peptide/GCG-OH).

Source data

**Extended Data Table 3:**
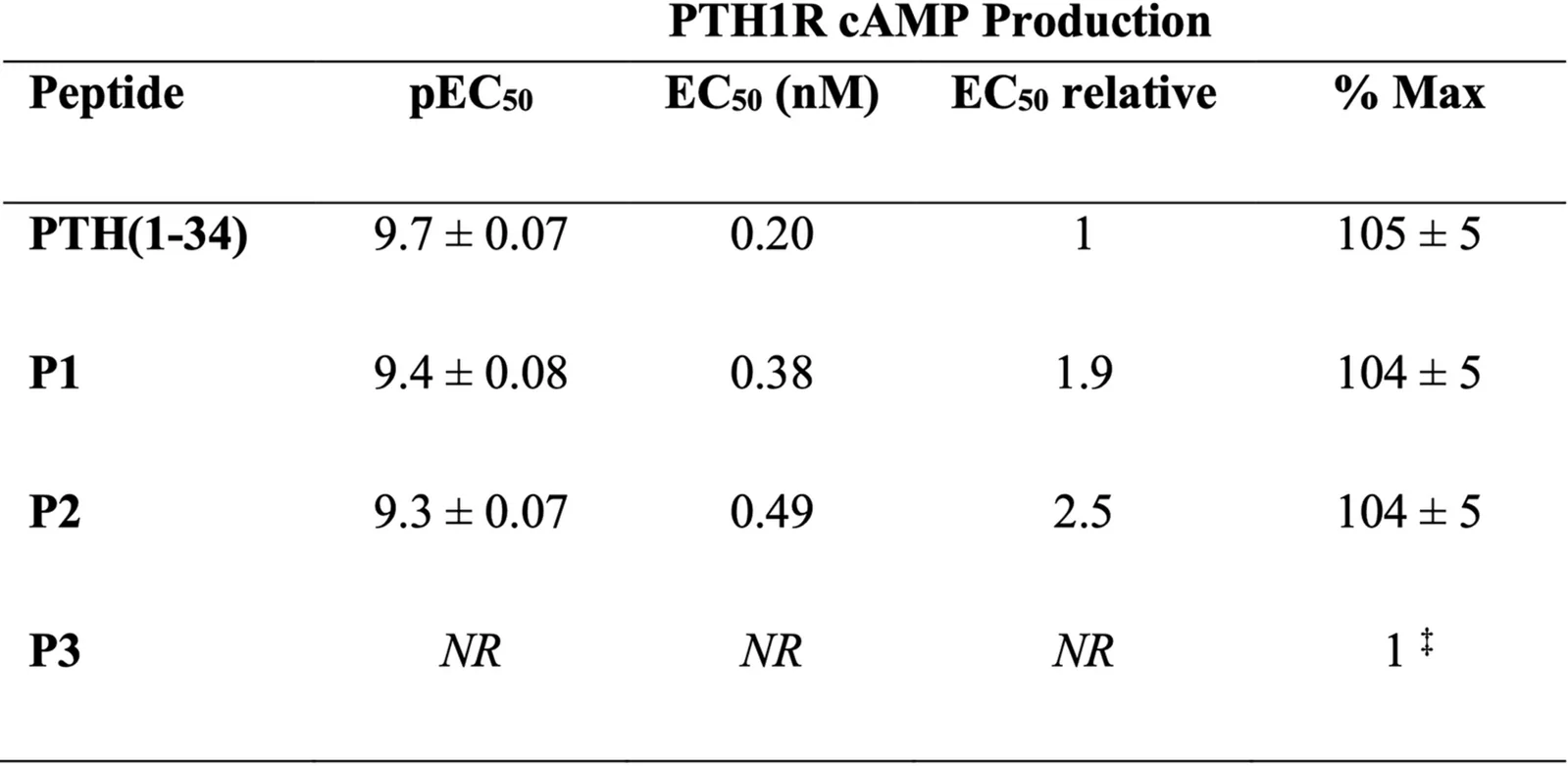
cAMP production potency and maximal response values for PTH(1-34) and heterochiral analogues P1–P3

pEC_50_, EC_50_ and % Max values are derived from 6 independent experiments (Fig. 4b). pEC_50_ indicates the negative logarithm of the half-maximal effective concentration (EC_50_). EC_50_ relative implies the cAMP potency normalized to PTH(1-34) (heterochiral analogue/PTH(1-34)). pEC_50_ and % Max uncertainties are expressed as s.e.m. ^‡^ Value shown represents the activity measured at highest agonist concentration evaluated—the dose—response curve failed to reach a maximum. *NR,* no response.

Source data

**Extended Data Table 4:**
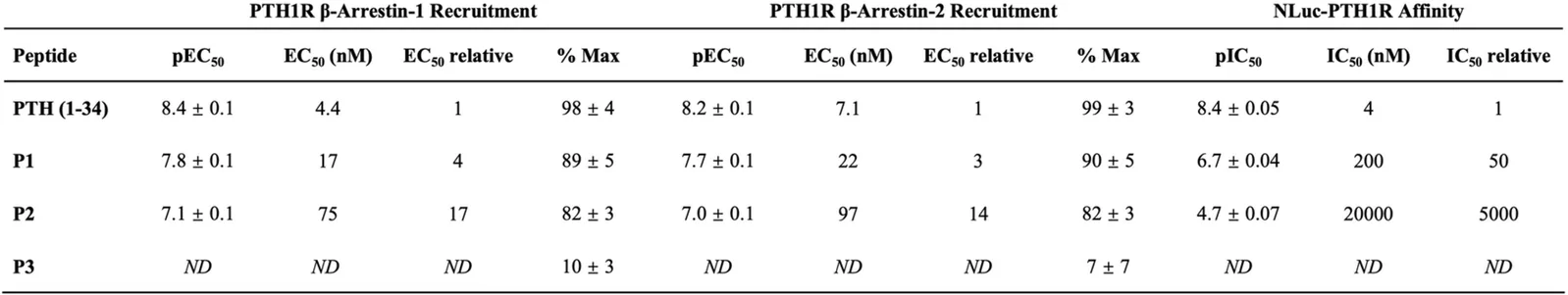
PTH1R β-arrestin-1 and −2 recruitment and NLuc-PTH1R affinity for PTH(1-34) and heterochiral analogues P1–P3

The ability of PTH(1-34) and heterochiral analogues to induce β-arrestin-1 or -2 recruitment to the PTH1R. pEC_50_, EC_50_ and % Max values are derived from ≥5 independent experiments (Fig. 4d, e). pEC_50_ indicates the negative logarithm of the half-maximal effective concentration (EC_50_). NLuc-PTH1R affinity pIC_50_ and IC_50_ values are the average of ≥3 independent experiments (Fig. 4h). pIC_50_ indicates the negative logarithm of the half-maximal inhibitory concentration (IC_50_). All EC_50_ relative and IC_50_ relative values for β-arrestin-1 and -2 recruitment and NLuc-PTH1R affinity were normalized to the values for PTH(1-34) (heterochiral analogue/PTH(1-34)). All pEC50, pIC_50_ and % Max uncertainties are expressed as s.e.m. *ND*,not determined.

## Data Availability

Data reported in this study are available in the Article, Supplementary Information and source data files. Previously published structural models used in Supplementary Fig. 2 are available from the Protein Data Bank (PDB) under accession numbers 6LMK and 8FLQ. MD simulations can be downloaded from the public repository Zenodo at https://zenodo.org/records/19096161 (ref. ^106^). Source data are provided with this paper.
