# Supplementary material for: Potent and biased agonists of class B1 GPCRs from a heterochiral design strategy
Source: Nat Chem. 2026 Jun 16;18(9):1639–49. doi: 10.1038/s41557-026-02182-x (PMC13524830; doi:10.1038/s41557-026-02182-x)
Supplement: Supplementary file 1 [file 41557_2026_2182_MOESM1_ESM.pdf]

nature chemistry

Supplementary information https://doi.org/10.1038/s41557-026-02182-x

Potent and biased agonists of class B1 GPCRs from a heterochiral design strategy

In the format provided by the authors and unedited

Supplementary Information – Table of Contents

I: Supplementary Figures 1-15 2

II: Supplementary Tables 1-10 17

III: Supplementary Note 28

A: Supplementary Methods 28
B: Plasmid sequences 31
C: Instrumentation and Instrumentation Acknowledgments 33
D: Peptide Characterization and Purity 34

IV: Supplementary References 54

I: Supplementary Figures 1-15

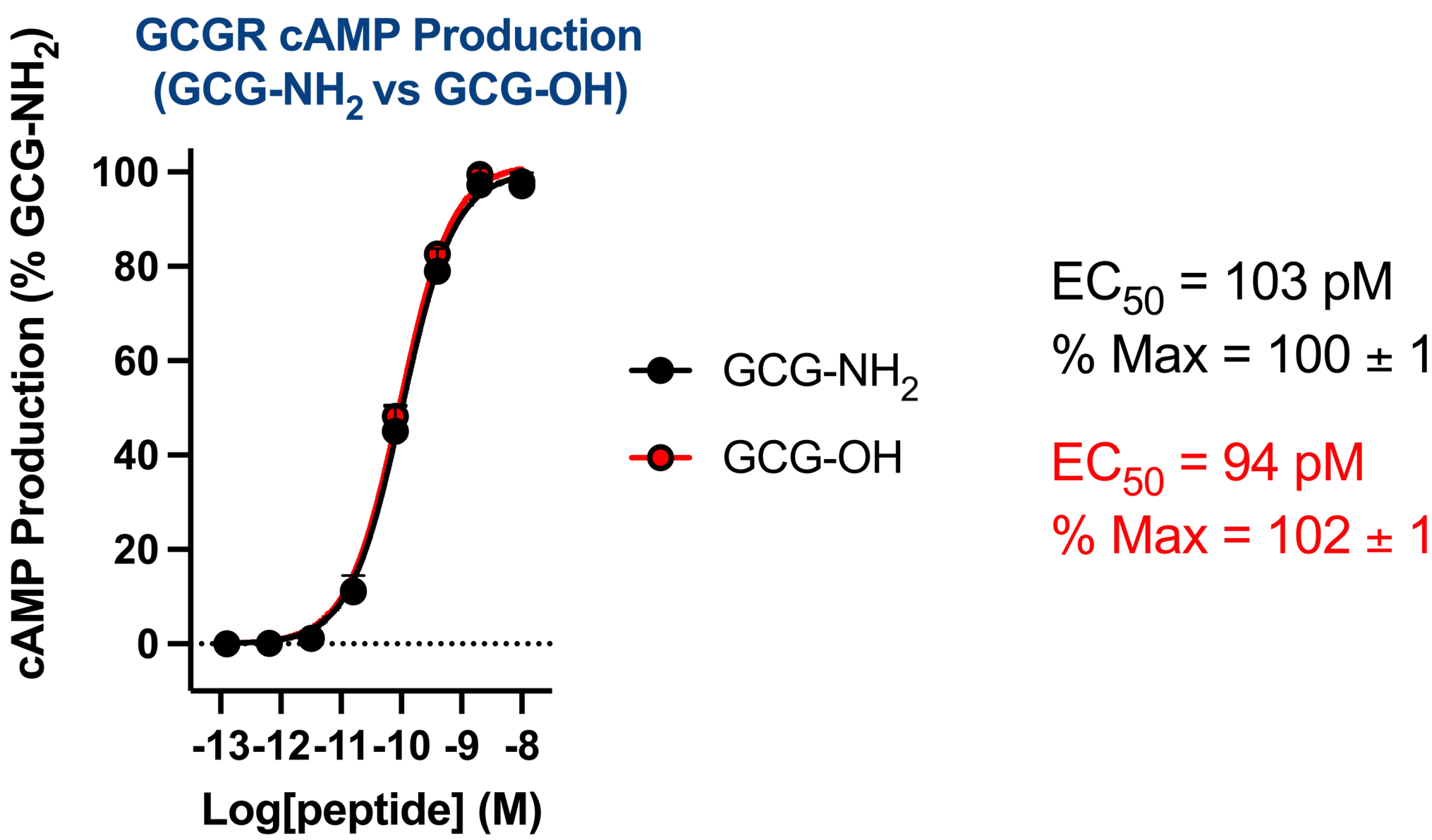

Supplementary Fig. 1: Comparison of cAMP production at the GCGR by GCG-NH_2 and GCG-OH (native form). The assay measured levels of cAMP production in HEK293 cells transiently expressing the GCGR and stably expressing the cAMP-sensing protein GloSensor. Data points represent the average of ≥10 independent experiments (n=13 for GCG-NH_2, n=10 for GCG-OH). Error bar uncertainties are expressed as S.E.M.

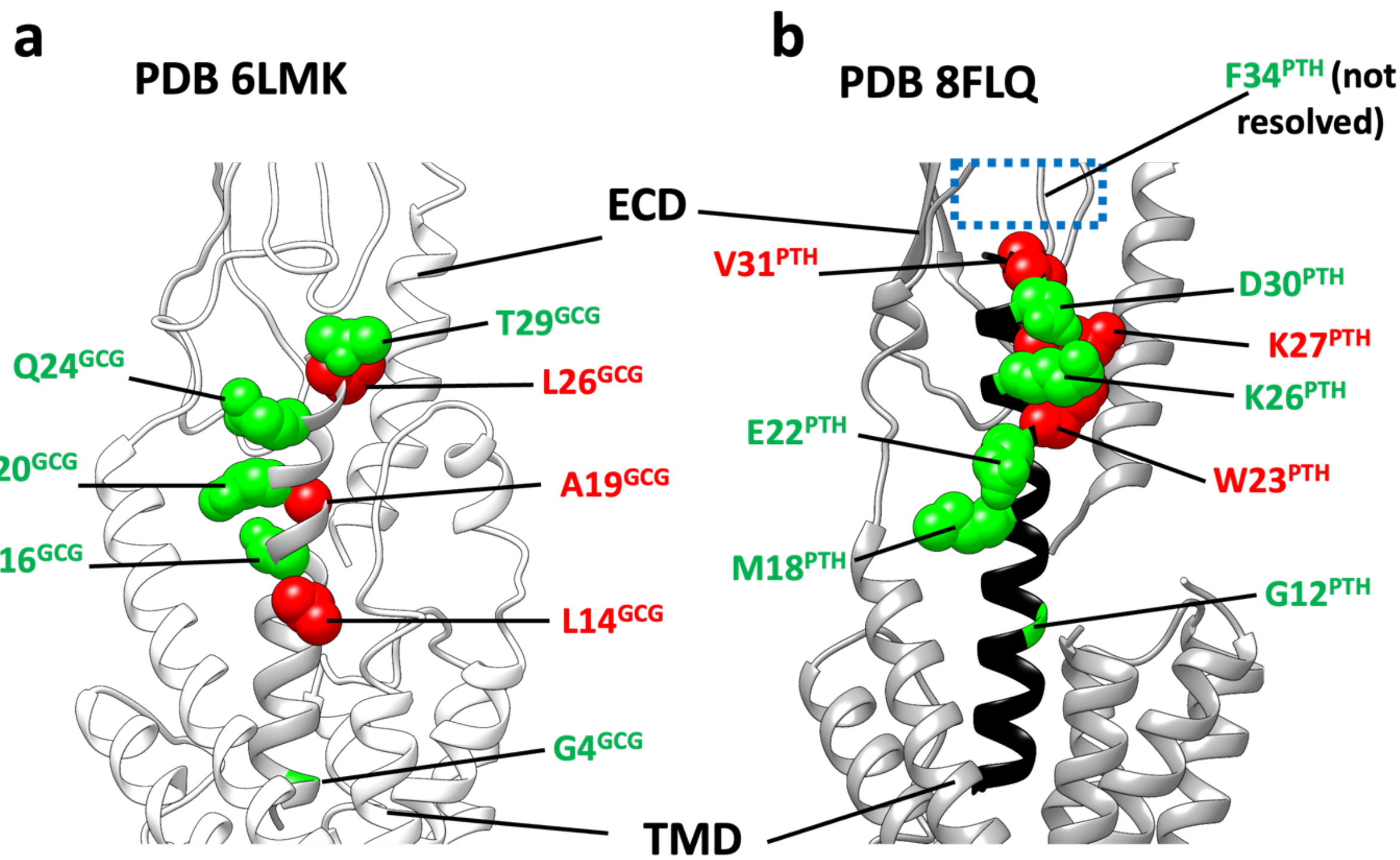

Supplementary Fig. 2: L-residue positions modified in this work, mapped onto glucagon bound to the GCGR (PDB 6LMK, a; glucagon is the grey ribbon) or PTH(1-34) bound to the PTHR1 (PDB 8FLQ, b; PTH(1-34) is the black ribbon). Red van der Waals spheres correspond to peptide residues that form direct interactions with either the ECD (extracellular domain) or TMD (transmembrane domain) of the receptor and that produce inactive peptides (cAMP production assay, Fig. 1b and 4b) if modified to the D-form (**G4** and **P3**). Green van der Waals spheres correspond to residues that do not form direct interactions with either the ECD or TMD of the receptor and that produce active peptides (**G1-G3**, **P1-P2**) in cAMP production assays (Fig. 1b and 4b).

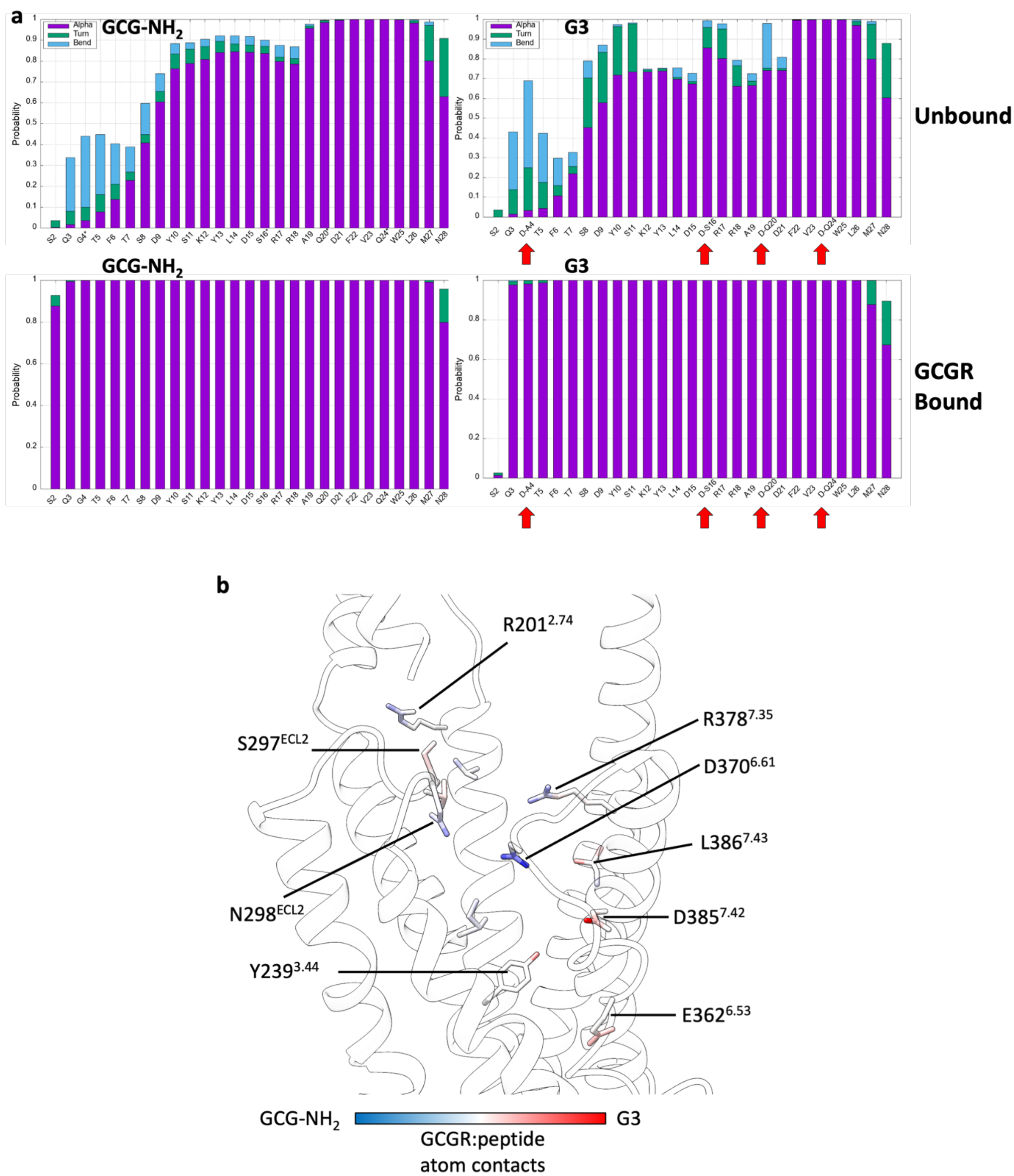

Supplementary Fig. 3: DSSP (*Define Secondary Structure of Proteins* algorithm) analysis of GCG-NH$_2$ and G3. a, DSSP analysis showing GCG-NH$_2$ and **G3** per-residue propensity to form an α-helix (magenta), a β-turn (blue), or a bend (green), during 30 µs of MD simulations in simulated water solution or when bound to the GCGR; red arrows indicate the positions 4, 16, 20, and 24 of the peptide. H1 and T29 are not shown because the backbone of a terminal residue cannot form a secondary structure according to the DSSP algorithm. **b,** Comparison of the total atomic contacts formed by the GCGR (ribbon) in complex with GCG-NH$_2$ or **G3**. Only side chains involved in main differences are shown as sticks; in blue are reported atoms more engaged by GCG-NH$_2$ and in red atoms more engaged by **G3**. Neither peptide is shown.

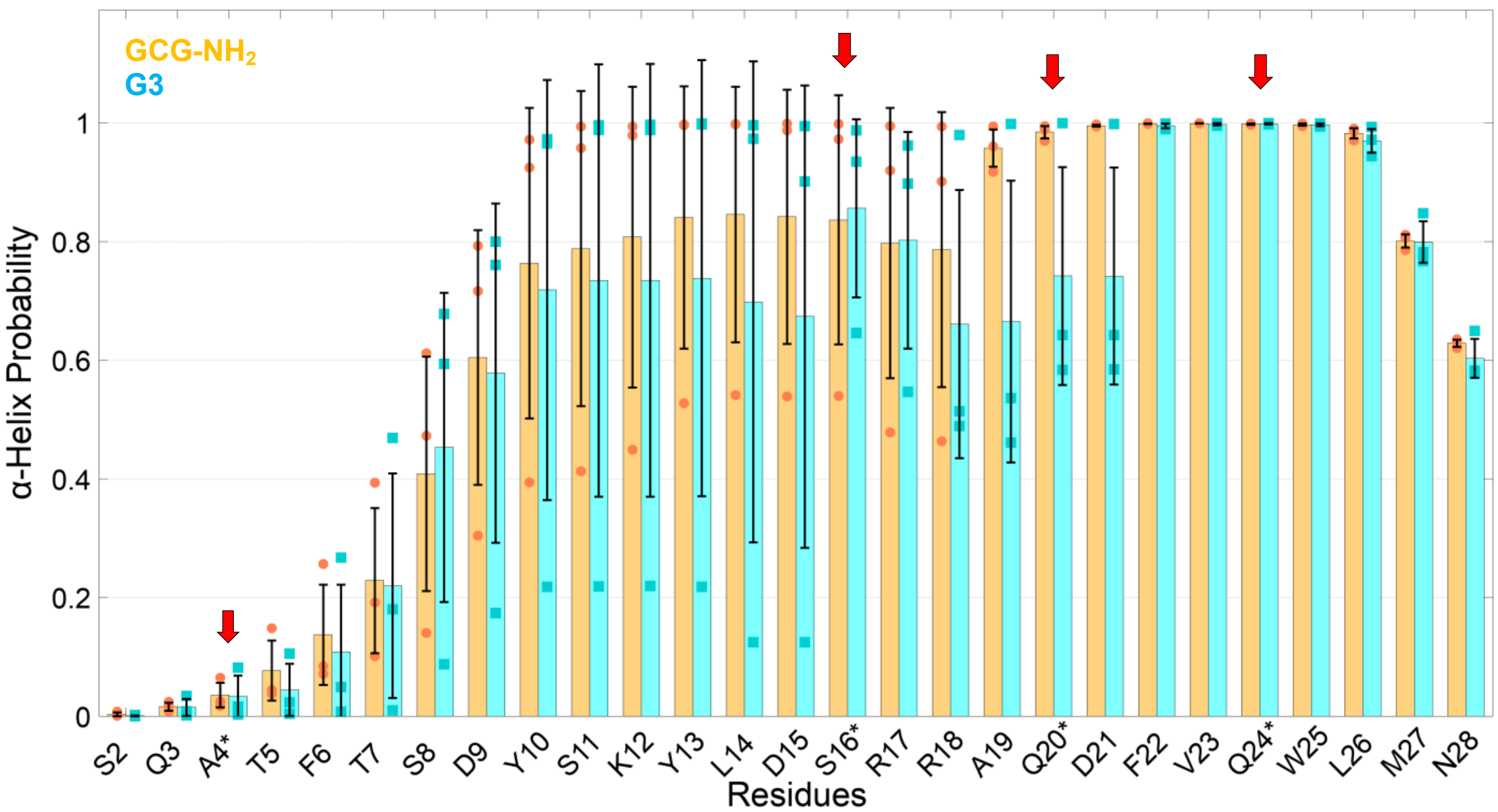

Supplementary Fig. 4: α-Helix probability measurements for GCG-NH_2 and G3 in simulated water solution. Comparison of the α-helix propensity of GCG-NH_2 and **G3** during 30 µs of MD simulation in simulated water solution; standard deviations are computed on three independent replicas of 10 µs each; red arrows indicate residues 4, 16, 20, and 24 of the peptide. His1 and Thr29 are not shown because the backbone of a terminal residue cannot form a secondary structure according to the database of secondary structure (DSSP) assignments algorithm. α-helix probability is expressed as Mean ± S.D. of three independent MD replicas of 10 µs each.

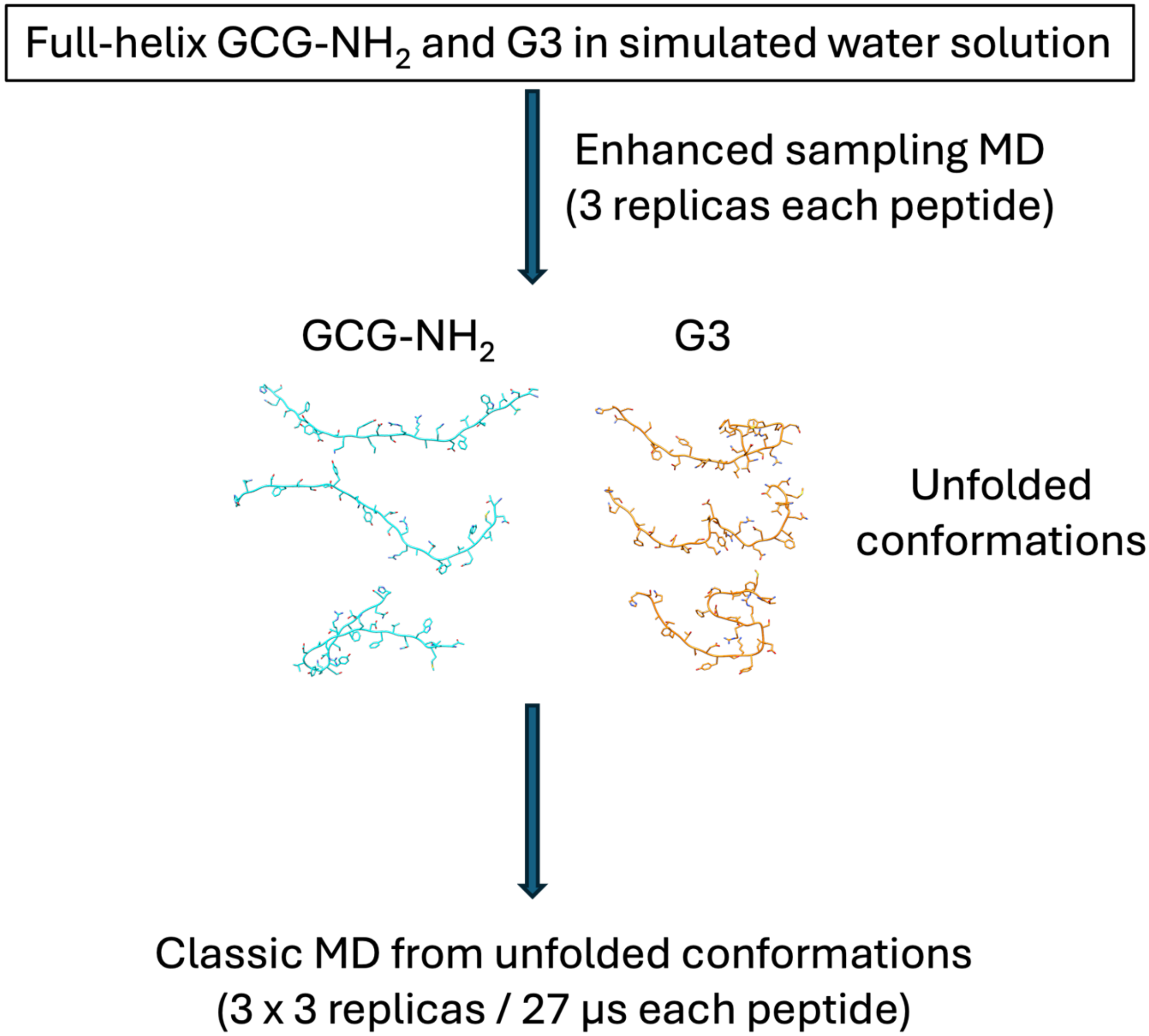

Supplementary Fig. 5: The workflow of molecular dynamics (MD) enhanced sampling protocol. The MD enhanced sampling protocol allowed the sampling of unfolded states of GCG-NH_2 and **G3** in simulated water solutions. Extensive classic MD simulations were successively seeded from peptides' unfolded states to sample the α-helix propensity of each peptide.

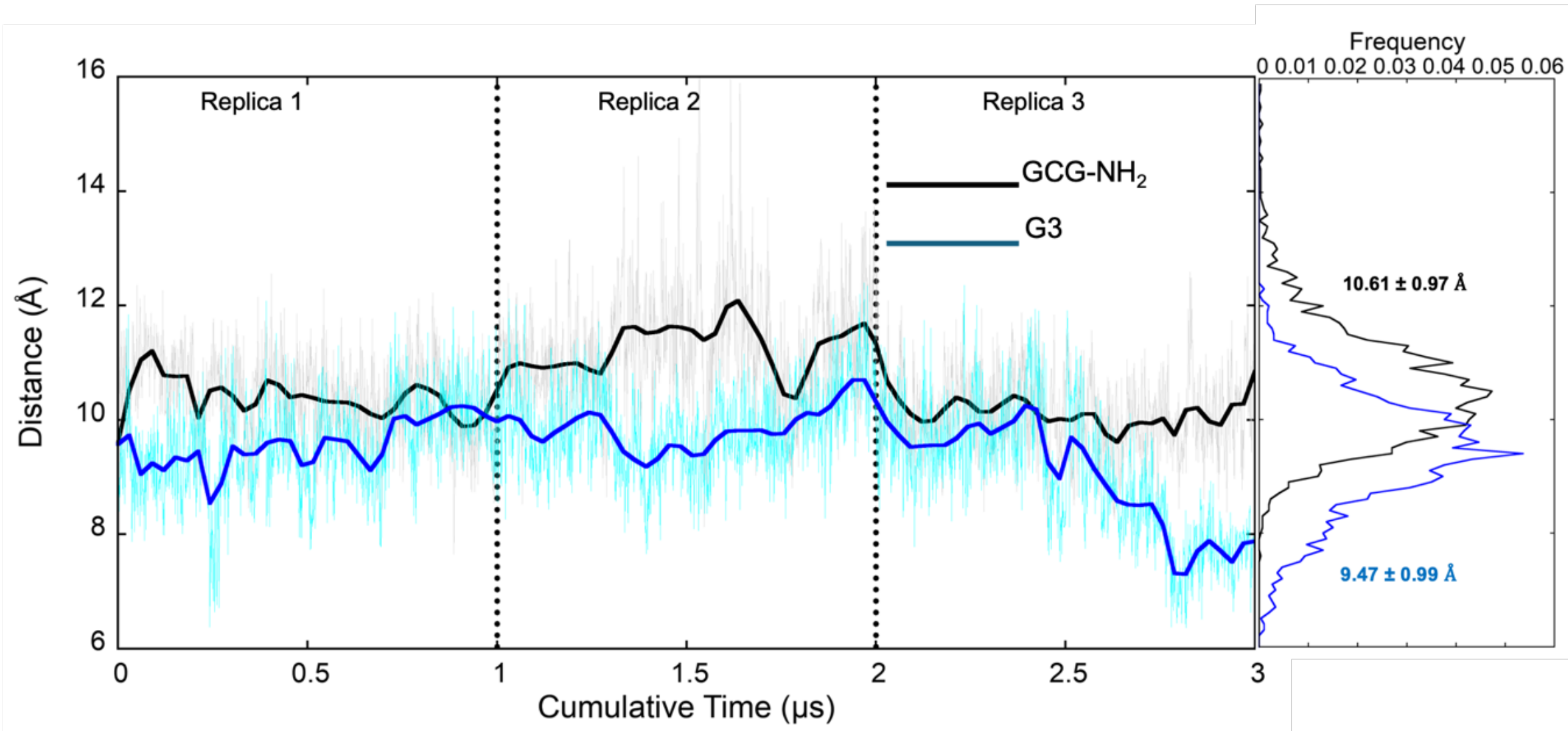

Supplementary Fig. 6: Measured peptide-TM1 distances based on MD simulations of peptide:GCGR complexes shown in Fig. 3b,c. Peptide-TM1 distance measured between the Cα of Ser16 (GCG-NH_2) or D-Ser16 (**G3**) and Gln131$^{1.29}$ of TM1. Black (GCG-NH_2) and blue (G3) bold lines are data interpolations.

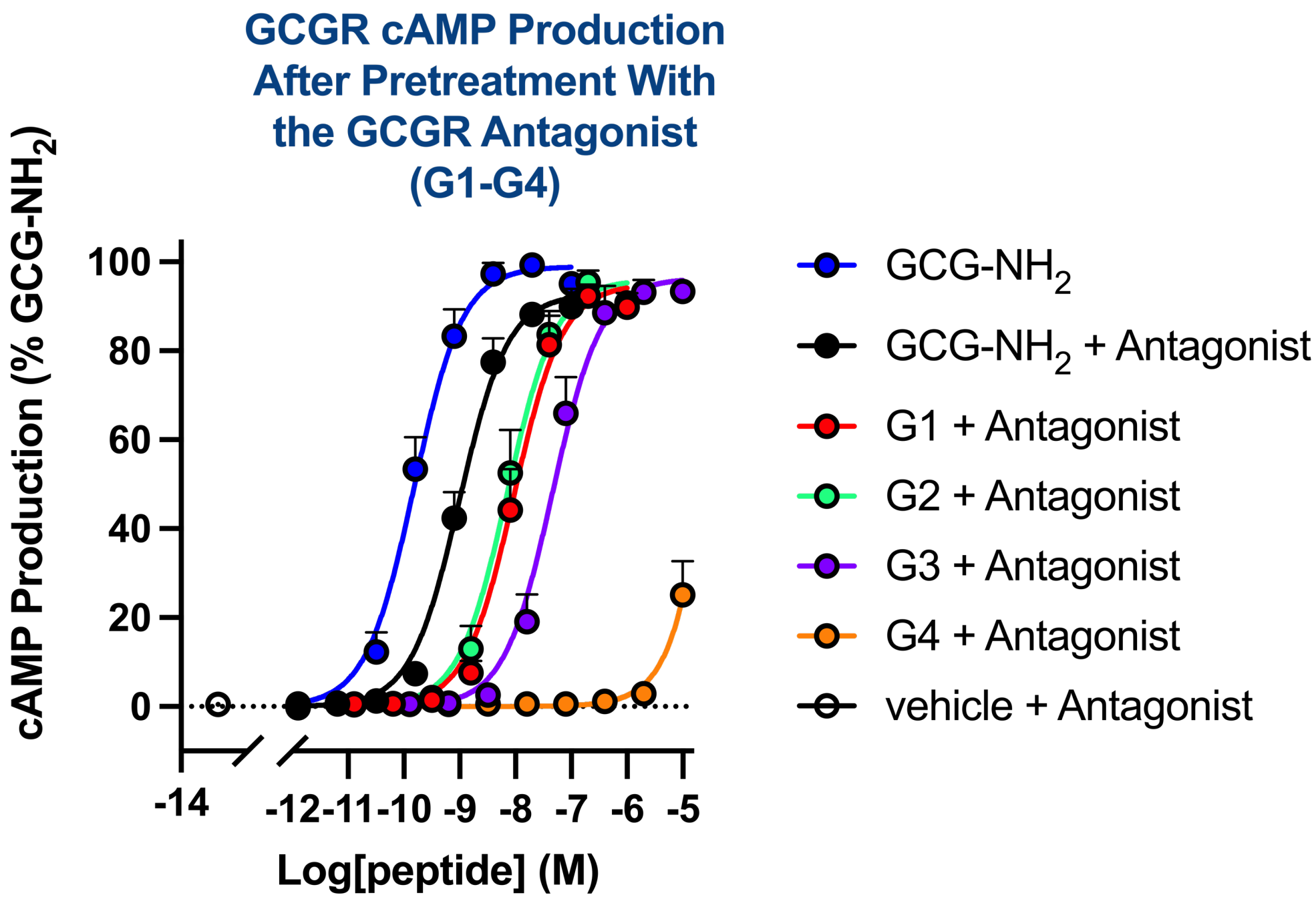

Supplementary Fig. 7: Stimulation of cAMP production at the GCGR by GCG-NH$_2$ and heterochiral analogues after pretreatment with a GCGR antagonist. The assay measured levels of cAMP production in HEK293 cells transiently expressing the GCGR and stably expressing the cAMP-sensing protein GloSensor. The cells were first treated with the antagonist peptide (100 nM) for 20 min, after which cAMP levels were recorded. Immediately after, cells were treated with one of the agonists (GCG-NH$_2$, **G1**-**G4**) or avehicle before cAMP levels were recorded once again; this set of cAMP levels was used to generate dose-response curves shown above. Data points represent the average of 6 independent experiments for all conditions except for "vehicle + Antagonist" (3 independent experiments). All data were normalized against GCG-NH$_2$ (i.e., GCG-NH$_2$ without any added antagonist). Error bar uncertainties are expressed as S.E.M.

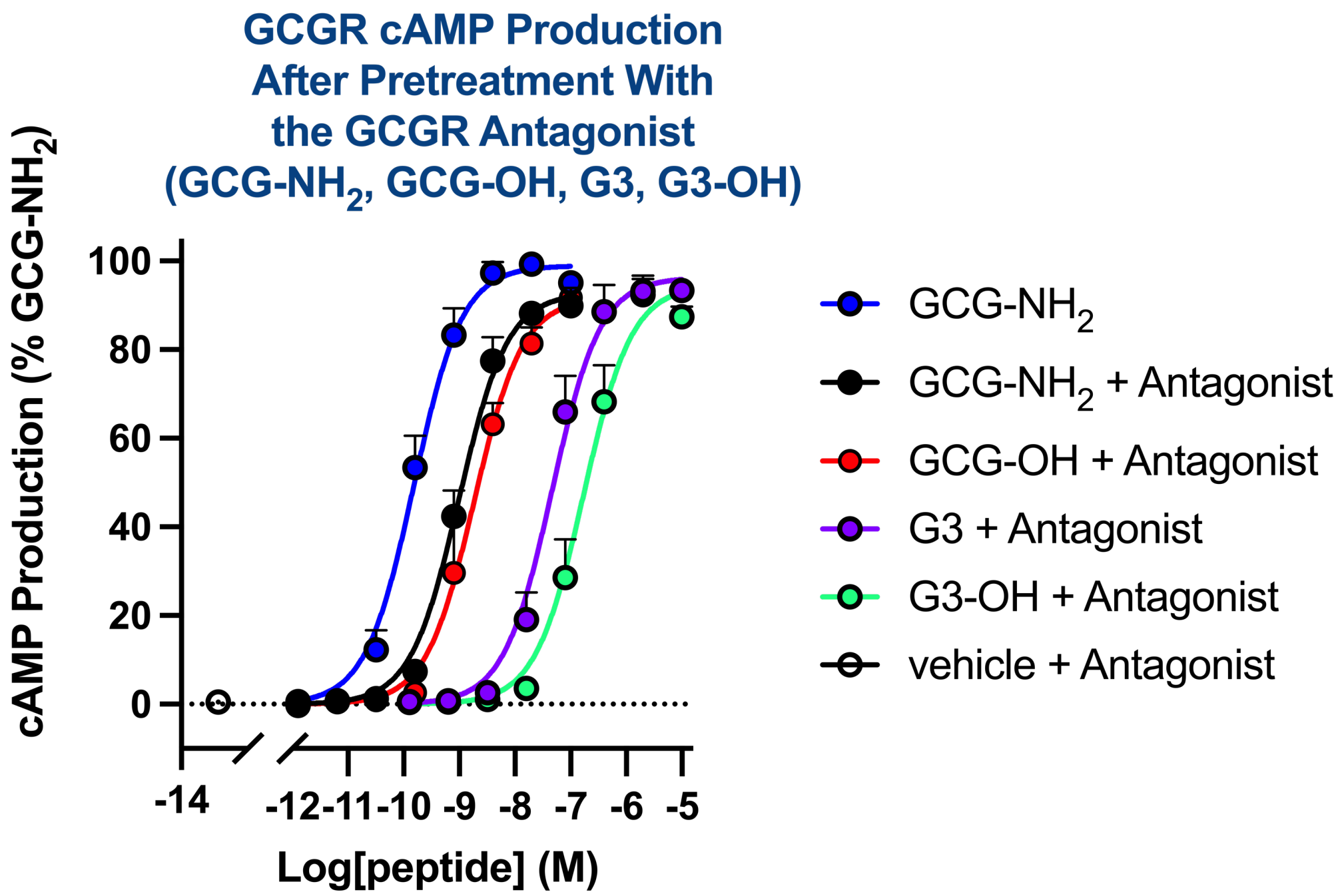

Supplementary Fig. 8: Stimulation of cAMP production at the GCGR by GCG-NH_2, GCG-OH, and heterochiral analogues, G3 or G3-OH, after pretreatment with the GCGR antagonist. The assay measured levels of cAMP production in HEK293 cells transiently expressing the GCGR and stably expressing the cAMP-sensing protein GloSensor. The cells were first treated with the antagonist peptide (100 nM) for 20 min, after which cAMP levels were recorded. Immediately after, cells were treated with one of the agonists or vehicle before cAMP levels were recorded once again; this new set of cAMP measurements was used to generate dose-response curves shown above. Data points represent the average of 6 independent experiments for all conditions except for "vehicle + Antagonist" (3 independent experiments). All data were normalized against GCG-NH_2 (i.e., GCG-NH_2 without any added antagonist). Error bar uncertainties are expressed as S.E.M.

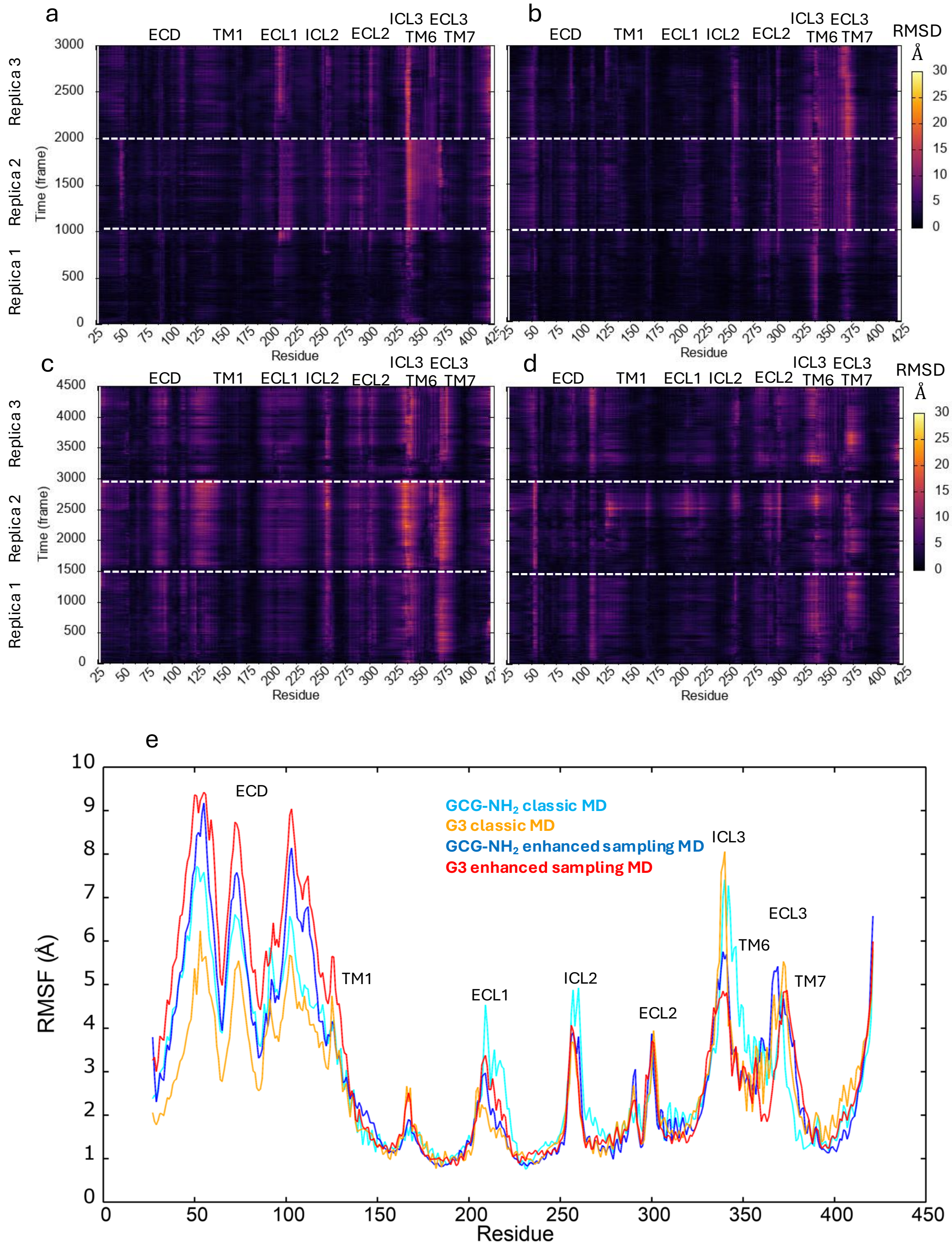

Supplementary Fig. 9: a-d, Reporting of root mean square deviation (RMSD) and root mean square fluctuation (RMSF) for GCGR Cα atoms when the receptor is bound to GCG-NH_2 or G3. Analysis reporting per-residue, per-frame root mean square deviation (RMSD) of GCGR Cα atoms when the receptor is bound to: (**a**) GCG-NH_2 (classic MD), (**b**) **G3** (classic MD), (**c**) GCG-NH_2 (enhanced MD), and (**d**) **G3** (enhanced MD). For each system, three MD replicas (1.0 µs or 1.5 µs each) were concatenated. **e,** Per-residue root mean square fluctuation (RMSF) computed for GCGR Cα atoms when the receptor is bound to GCG-NH_2 (classic MD, cyan), **G3** (classic MD, orange), GCG-NH_2 (enhanced MD, blue), or **G3** (enhanced MD, red).

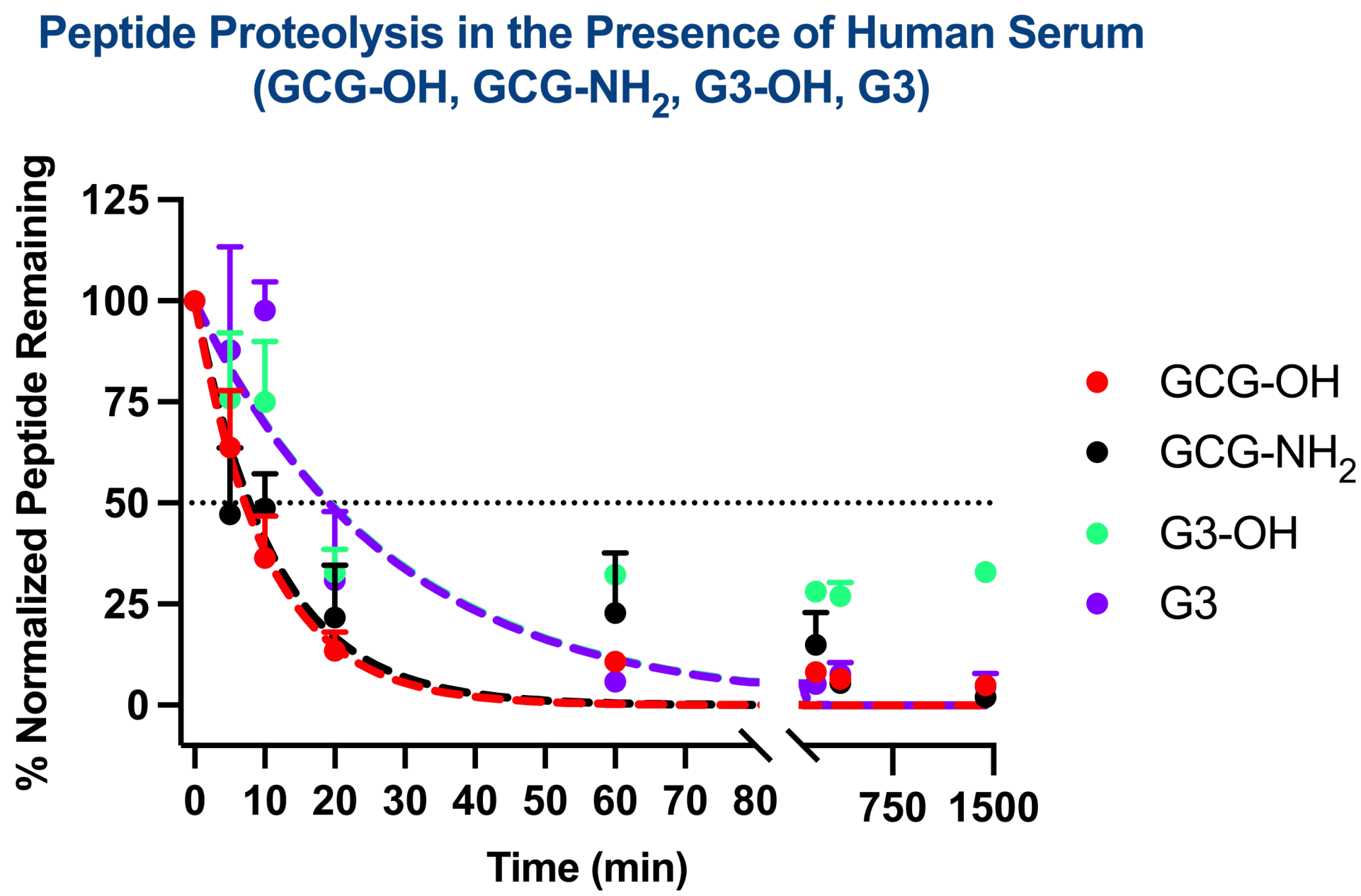

Supplementary Fig. 10: Time course of degradation of GCG-OH, GCG-NH$_2$, G3-OH or G3 in the presence of human serum, as assessed by UPLC. (See Supplementary Methods section for more details.) Data points represent the average of 3 independent experiments. All uncertainties are expressed as S.E.M. The x-axis (Time (min)) was divided into two segments for clarity; no data points were omitted.

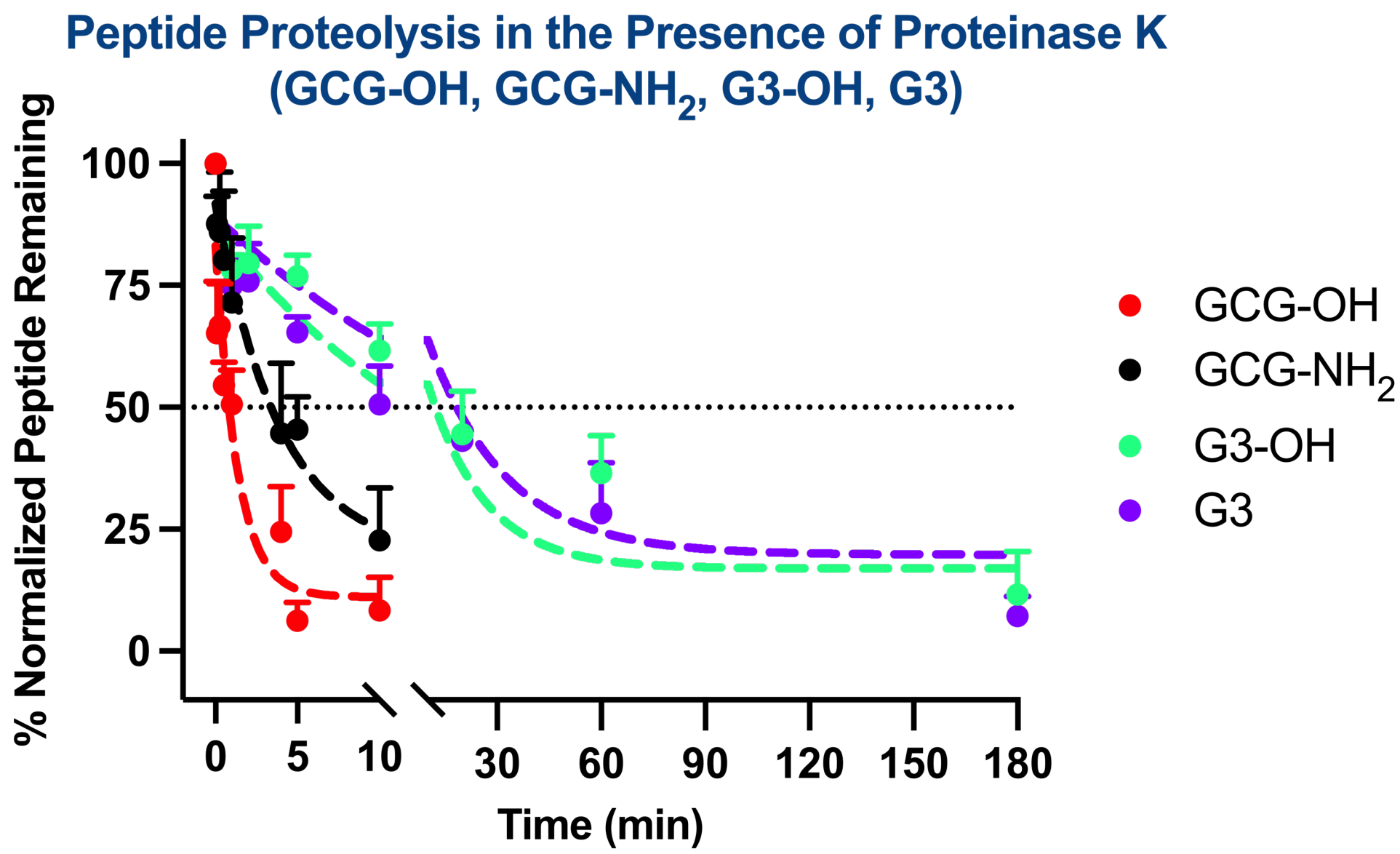

Supplementary Fig. 11: Time course of degradation of GCG-OH, GCG-NH$_2$, G3-OH or G3 in the presence of proteinase K, as assessed by UPLC. (See Methods section for more details.) Data points represent the average of 3 independent experiments. All uncertainties are expressed as S.E.M. The x-axis (Time (min)) was divided into two segments for clarity; no data points were omitted.

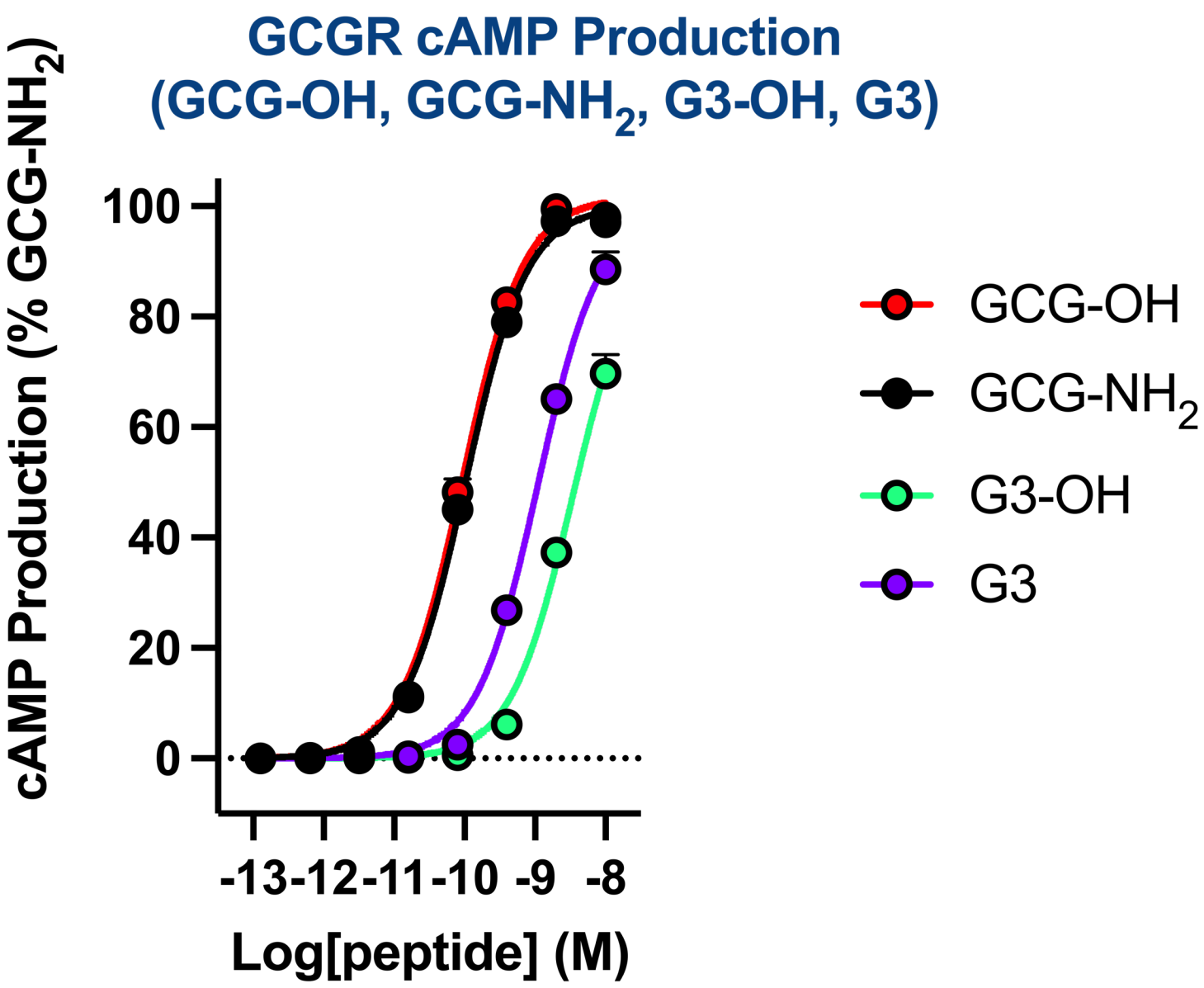

Supplementary Fig. 12: Stimulation of cAMP production at the GCGR by different C-terminal forms of GCG (glucagon) and G3. The assay measured levels of cAMP production in HEK293 cells transiently expressing the GCGR and stably expressing the cAMP-sensing protein GloSensor. Data points represent the average of ≥5 independent experiments (n=10 for GCG-OH, n=13 for GCG-NH₂, n=5 for **G3-OH**, n=8 for **G3**). Error bar uncertainties are expressed as S.E.M.

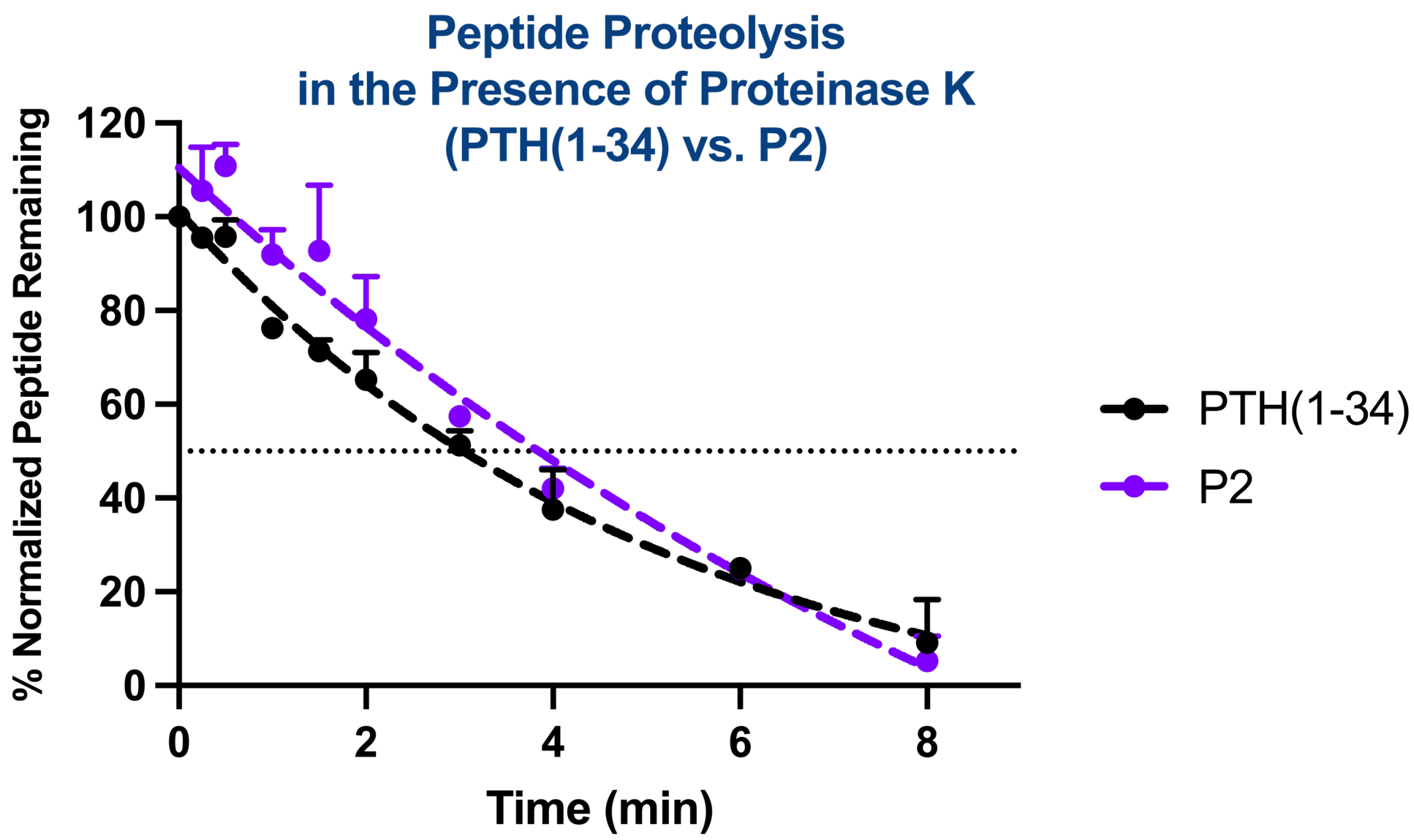

Supplementary Fig. 13: Time course of degradation of PTH(1-34) or P2 in the presence of proteinase K, as assessed by UPLC. (See Supplementary Methods section for more details.) Data points represent the average of 2 independent experiments. All uncertainties are expressed as S.E.M.

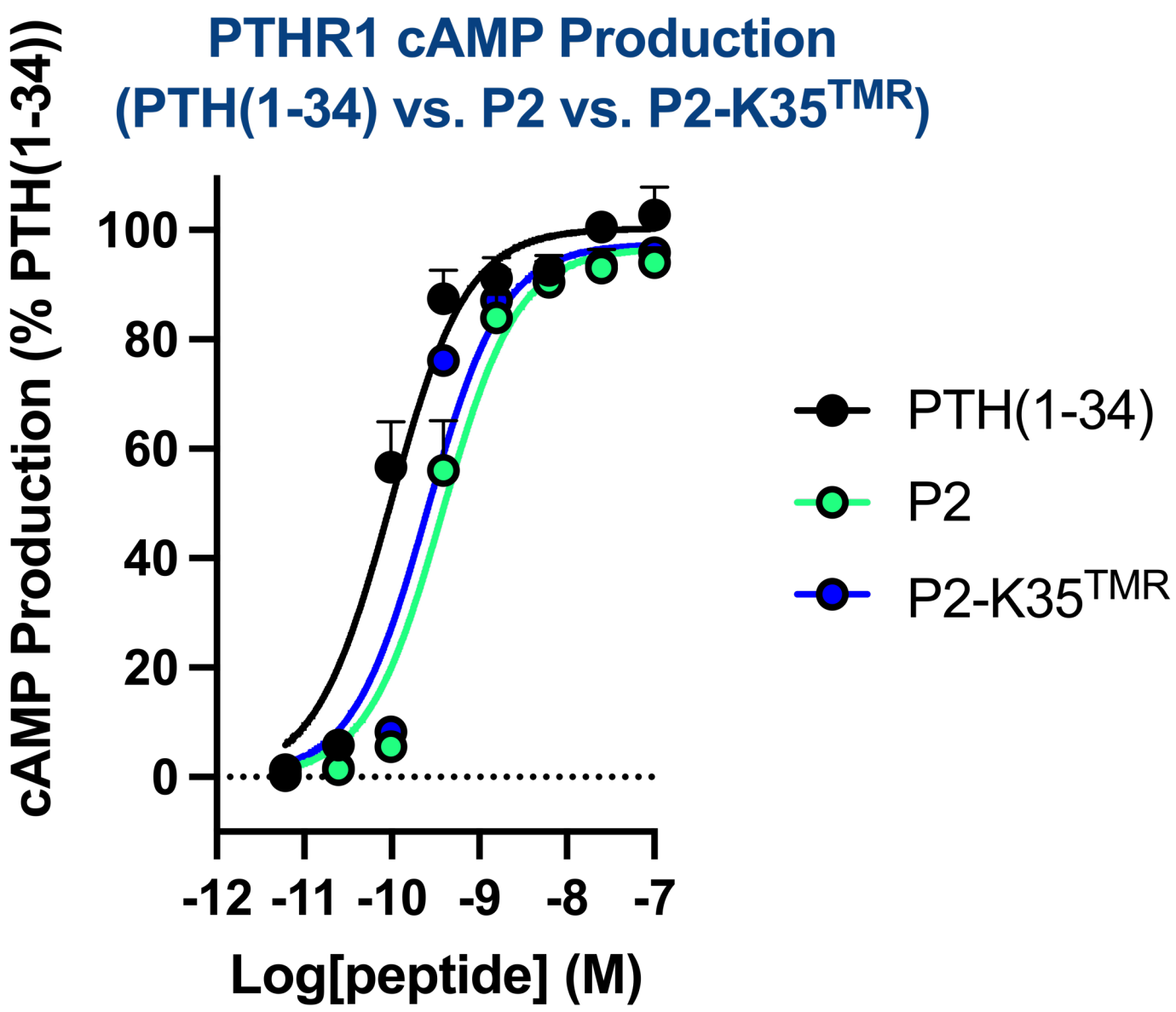

Supplementary Fig. 14: Stimulation of cAMP production profiles at the PTHR1 by PTH(1-34), P2 and P2-K35^{TMR}. The assay monitored levels of cAMP production in HEK293 cells stably expressing the PTHR1 and the cAMP-sensing protein GloSensor. Data points represent the average of 3 independent experiments. All data were normalized against PTH(1-34), and all uncertainties are expressed as S.E.M. **P2**-K35^{TMR} was a tetramethylrhodamine (TMR)-labeled derivative of **P2**, P2(1-35)-(Lys35-TMR)-NH_2.

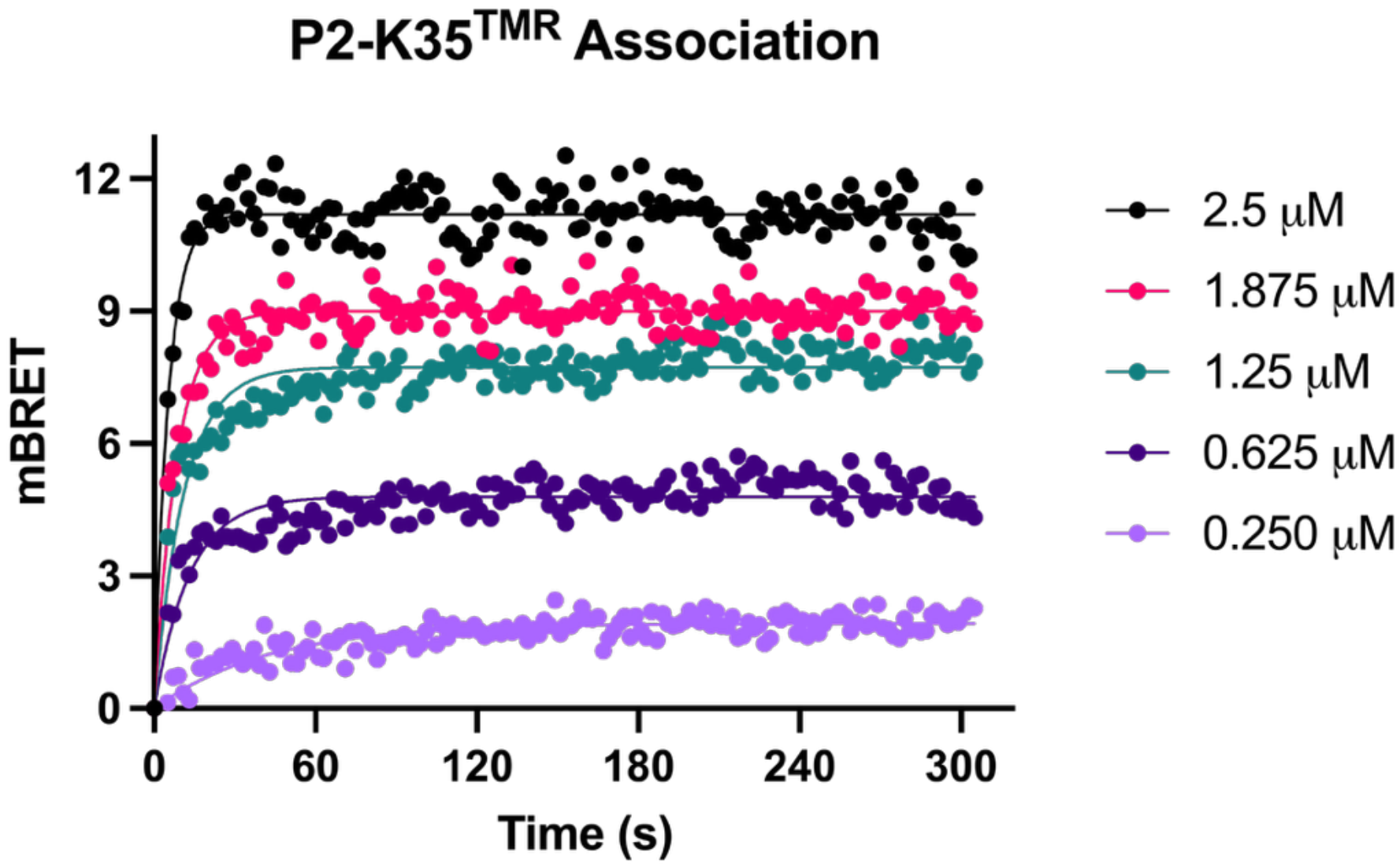

k_{on} (x10^5 s^{-1} M^{-1})	k_{off} (s^{-1})	K_D (nM)
0.61 ± 0.2	0.030 ± 0.003	492

Supplementary Fig. 15: Kinetic direct binding measurements for P2-K35TMR at the NLuc-PTHR1. Representative milli bioluminescence resonance energy transfer (mBRET) data for heterochiral **P2-**K35^{TMR} binding to the NLuc-PTHR1 at five different peptide concentrations measured over 300 seconds (s). Each trace shows the result of a single measurement. Calculated k_{on}, k_{off} and K_D values were derived from 6 independent experiments.

II: Supplementary Tables 1-10

Supplementary Table 1. Intermolecular hydrogen bonds formed by N-terminal residues of GCG-NH_2 or G3 and the receptor during MD simulations of each peptide bound to the GCGR. Interaction occupancy is reported as % MD frames in which they occurred.

GCG-NH_2 Residue	GCGR Residue	Occupancy (% MD frames)	G3 Residue	GCGR Residue	Occupancy (% MD frames)
H1	D370	75.7 (sb)	H1	D385	88.4 (sb)
	D385	34.5		D370	22.0 (sb)
	K187	33.4		E362	20.4
	D299	10.5 (sb)		E362	14.8
				Y239	16.5
				K187	9.2
S2	D385	88.1	S2	D385	57.7
	D385	58.0 (sb)		D385	45.5 (sb)
Q3	Y149	14.9	Q3	K187	32.4
	Y145	13.2		D385	29.4
				Y145	27.7
				Y149	25.5
T5	D370	46.0	T5	R378	18.2
	R378	19.5			

sb = hydrogen bond between side chain and backbone atoms.

Supplementary Table 2. Intramolecular hydrogen bonds formed by residues 4, 16, 20, 24, and 29 of GCG-NH_2 or G3 during MD simulations of each peptide bound to the GCGR. Interaction occupancy is reported as % MD frames in which they occurred.

GCG-NH_2 Residue	GCG Residue	Occupancy (% MD frames)	G3 Residue	G3 Residue	Occupancy (% MD frames)
G4	S8	26.1 (sb)	D-A4	H1	11.0 (sb)
	Q3	21.7 (sb)		T7	10.7 (sb)
	H1	14.3 (sb)		S8	8.9 (sb)
S16	K12	41.1 (sb)	D-S16	D-Q20	35.8 (sb)
	Q20	25.9 (sb)		D-Q20	14.4
	Q20	8.5			
Q20	Q24	8.3 (sb)	D-Q20	D-Q24	44.6 (sb)
	R17	6.3		R17	14.1
Q24	N28	13.7	D-Q24	N28	14.2 (sb)
	N28	8.1 (sb)			
T29	W25	92.9 (sb)	D-T29	N.D (W25)	N.D. (65.5 (sb))

N.D. = occupancy < 5 %; sb = hydrogen bond between side chain and backbone atoms.

Supplementary Table 3. Hydrogen bonds between the GCGR and GCG-NH_2 or G3 residues 4, 16, 20, 24, 29 and part of the N-terminus during MD simulations. Interaction occupancy is reported as % MD frames in which they occurred.

GCG-NH_2 Residue	GCGR Residue	Occupancy (% MD frames)	G3 Residue	GCGR Residue	Occupancy (% MD frames)
G4	N.D	N.D	D-A4	N.D	N.D
S16	N.D	N.D	D-S16	N.D	N.D
Q20	$Q122^{ECD}$	18.1 (wb)	D-Q20	$Q122^{ECD}$	8.1 (wb)
Q20	$Q131^{1.29}$	14.4	D-Q20	$Q131^{1.29}$	9.5
		17.0 (wb)			17.9 (wb)
Q24	$Q122^{ECD}$	14.8	D-Q24	$Q122^{ECD}$	5.1
		38.8 (wb)			10.7 (wb)
T29	$R116^{ECD}$	77.9 (sb)	D-T29	$K64^{ECD}$	6.3

N.D. = occupancy < 5 %; sb = hydrogen bond between side chain and backbone atoms; wb = hydrogen bond mediated by a water molecule.

Supplementary Table 4. Computed binding energies of GCG-NH_2:GCGR and G3:GCGR from MD simulations.

GCG-NH_2:GCGR (kcal mol^{-1})	G3:GCGR (kcal mol^{-1})
-128.1 ± 20.8	-113.3 ± 18.6

Molecular mechanics energies combined with the generalized Born and surface area continuum solvation (MM/GBSA) of simulation for both GCG-NH2:GCGR and **G3**:GCGR complexes. The computed binding energy for **G3**:GCGR complex was statistically different from that of GCG-NH_2:GCGR complex (the t_score is 20.614 (p value = 2.0806562852324225e-88)). Computed binding energies are expressed as mean ± S.D. (computed on 1500 molecular dynamics frames corresponding to 3 μs from three independent replicas).

Supplementary Table 5. cAMP production potency (pEC_{50}, EC_{50}, EC_{50} relative) and maximal response (% Max) values for GCG-NH_2, GCG-OH, G3 and G3-OH at the GCGR.

	GCGR cAMP Production			
**Peptide**	**pEC_{50}**	**EC_{50} (nM)**	**EC_{50} relative**	**% Max**
**GCG-OH**	10.0 ± 0.03	0.094	1	102 ± 1
**GCG-NH_2**	10.0 ± 0.02	0.10	1.1	100 ± 1
**G3-OH**	8.5 ± 0.05	3.32	35	70 ‡
**G3**	9.0 ± 0.03	1.10	12	88 ‡

pEC_{50}, EC_{50} and % Max values are derived from ≥5 independent experiments (n=10 for GCG-OH, n=13 for GCG-NH_2, n=5 for **G3-OH**, n=8 for **G3**). pEC_{50} indicates the negative logarithm of the half-maximal effective concentration (EC_{50}). EC_{50} relative implies the cAMP potency normalized to GCG-OH by the quotient (peptide/GCG-OH). pEC_{50} and % Max uncertainties are expressed as S.E.M. ‡ Value shown represents the activity measured at highest agonist concentration evaluated; dose-response curve failed to reach a maximum.

Supplementary Table 6. cAMP production potency (pEC_{50}, EC_{50}, EC_{50} relative) and maximal response (% Max) values for $GCG\text{-}NH_2$ and analogues when GCGR-expressing cells were pretreated with the antagonist.

	GCGR cAMP Production			
**Peptide**	**pEC_{50}**	**EC_{50} (nM)**	**EC_{50} relative**	**% Max**
**$GCG\text{-}NH_2$**	9.8 ± 0.06	0.15	0.15	99 ± 2
**$GCG\text{-}NH_2$ + Antagonist**	9.0 ± 0.06	0.99	1	93 ± 2
**G1 + Antagonist**	8.0 ± 0.07	9.4	9	95 ± 3
**G2 + Antagonist**	8.1 ± 0.07	7.1	7	96 ± 3
**G3 + Antagonist**	7.3 ± 0.07	46	46	96 ± 3
**G4 + Antagonist**	*ND*	*ND*	*ND*	25 ‡

pEC_{50}, EC_{50} and % Max values are derived from 6 independent experiments (data in Supplementary Fig. 7). pEC_{50} indicates the negative logarithm of the half-maximal effective concentration (EC_{50}). EC_{50} relative implies the cAMP potency normalized to the "$GCG\text{-}NH_2$ + Antagonist" condition. pEC_{50} and % Max uncertainties are expressed as S.E.M. *ND* = Not Determined. ‡ Value shown represents the activity measured at the highest agonist concentration evaluated; dose-response curve failed to reach a maximum.

Supplementary Table 7. cAMP production potency (pEC_{50}, EC_{50}, EC_{50} relative) and maximal response (% Max) values for GCG-NH_2, GCG-OH, G3 and G3-OH when GCGR-expressing cells were pretreated with the antagonist.

GCGR cAMP Production

Peptide	pEC_{50}	EC_{50} (nM)	EC_{50} relative	% Max
**GCG-NH_2**	9.8 ± 0.06	0.15	0.15	99 ± 2
**GCG-NH_2 + Antagonist**	9.0 ± 0.06	0.99	1	93 ± 2
**GCG-OH + Antagonist**	8.7 ± 0.09	1.8	2	92 ± 4
**G3 + Antagonist**	7.3 ± 0.07	46	46	96 ± 3
**G3-OH + Antagonist**	6.8 ± 0.08	170	170	95 ± 4

pEC_{50}, EC_{50} and % Max values are derived from 6 independent experiments (data in Supplementary Fig. 8). pEC_{50} indicates the negative logarithm of the half-maximal effective concentration (EC_{50}). EC_{50} relative implies the cAMP potency normalized to the "GCG-NH_2 + Antagonist" condition. pEC_{50} and % Max uncertainties are expressed as S.E.M.

Supplementary Table 8. Half-life (min) in the presence of proteinase K for PTH(1-34) and P2.

	Half-life (min)	
**Peptide**	**$t_{1/2}$ (min)**	**$t_{1/2}$ (min) relative**
**PTH(1-34)**	2.9	1
**P2**	3.7	1.3

Half-life values for peptides in the presence of proteinase K, as determined from one-phase decay curves plotted using data obtained from 2 independent experiments. $t_{1/2}$ (min) relative indicates the half-life normalized to PTH(1-34): (heterochiral analogue/PTH(1-34)).

Supplementary Table 9. cAMP production potency (pEC_{50}, EC_{50}, EC_{50} relative) and maximal response (% Max) values for PTH(1-34), P2 and P2-K35^{TMR}.

	PTHR1 cAMP Production			
**Peptide**	**pEC_{50}**	**EC_{50} (nM)**	**EC_{50} relative**	**% Max**
**PTH(1-34)**	10.0 ± 0.08	0.10	1	100 ± 3
**P2**	9.4 ± 0.08	0.38	3.8	97 ± 3
**P2-K35^{TMR}**	9.6 ± 0.09	0.25	2.5	97 ± 3

pEC_{50}, EC_{50} and % Max values are derived from 3 independent experiments (data in Supplementary Fig. 14). pEC_{50} indicates the negative logarithm of the half-maximal effective concentration (EC_{50}). EC_{50} relative implies the cAMP potency normalized to PTH(1-34) (heterochiral analogue/PTH(1-34)). pEC_{50} and % Max uncertainties are expressed as S.E.M. **P2**-K35^{TMR} was a tetramethylrhodamine (TMR)-labeled derivative of **P2**, P2(1-35)-(Lys35-TMR)-NH_2.

Supplementary Table 10. Table of reagents and resources used in this work.

REAGENT / RESOURCE	SOURCE	CATALOG # / IDENTIFIER
**Solid-phase Peptide Synthesis**		
**α-N-Fmoc-L-amino acids**	Chem Impex	Various
**α-N-Fmoc-D-amino acids**	Chem Impex	Various
Fmoc-L-β^3-homotyrosine-OH	Chem Impex	12764
Acetonitrile HPLC-grade	Millipore Sigma	34851
Ethyl (hydroxyimino) cyanoacetate (Oxyma)	Chem Impex	26426
N,N′-Diisopropylcarbodiimide (DIC)	Chem Impex	00110
Piperidine ReagentPlus®	Millipore Sigma	104094
α-Cyano-4-hydroxycinnamic acid (CHCA) for MALDI	Millipore Sigma	70990
SPPS reaction vessel syringes/caps	Torviq	SF-1000/PC-SF
N, N Dimethylformamide (DMF) biotech-grade	Millipore Sigma	494488
N, N Dimethylformamide (DMF) ACS-grade	Millipore Sigma	319937
Trifluoroacetic acid (TFA) ReagentPlus®	Millipore Sigma	T6508
1,2 ethanedithiol	Millipore Sigma	02390
Thioanisole ReagentPlus®	Millipore Sigma	T28002
Triisopropylsilane (TIPS)	Millipore Sigma	233781
2,2′-(Ethylenedioxy)diethanethiol	Millipore Sigma	465178
Anisole ReagentPlus®	Millipore Sigma	123226
ProTide™ Rink Amide Resin (LL)	CEM	R002
**Materials for Cell Assays**		
D-Liciferin Potassium Salt	GoldBio	LUCK-100
Coelenterazine 400 a (DeepBlueC™)	GoldBio	C-320-10
Coelenterazine-H	Promega	S2001
FuGENE® HD Transfection Reagent	Promega	E2311
HEK293FT Cells	Thermo Fisher	R70007
HEK293GS22 (GloSensor™) Cells	Promega	E1261 (Kind gift from Thomas Gardella)
HEK293 GP2.3 cells (PTH1R + GloSensor™)	Promega	E1261 (Kind gift from Thomas Gardella)
HEK293H (+NLuc-PTH1R)	In house previously	Reference[1]
CHO-FlpIn (+PTH1R-RLuc8 and β-arrestin-1-Venus)	Kind gift from Denise Wooten and Patrick Sexton labs	Reference[2]
CHO-FlpIn (+PTH1R-RLuc8 and β-arrestin-2-Venus)	Kind gift from Denise Wooten and Patrick Sexton labs	Reference[2]
Transporter 5® (PEI MAX®) Transfection Reagent	Polysciences	26008-5
DMEM High Glucose	Gibco	11965092
Opti-MEM™ Reduced Serum Medium	Gibco	31985070
McCoy's 5A (Modified) Medium HEPES	Gibco	12330031
Dulbecco's Phosphate Buffered Saline (DPBS)	Millipore Sigma	D8537

Dulbecco's Phosphate Buffered Saline (DPBS) + Glucose	Gibco	14287080
L-Glutamine 200 mM (100X)	Gibco	25030081
Sodium Pyruvate 100 mM (100X)	Gibco	11360070
MEM Non-Essential Amino Acids Solution (100X)	Gibco	11140050
HyClone™ Penicillin-Streptomycin Solution (100X)	GE Healthcare Life Sciences	SV30010
Fetal Bovine Serum (FBS) Heat Inactivated	Gibco	10082147
10 cm Fisherbrand™ Surface Treated Tissue Culture Dishes	Fisher Scientific	FB012924
75 cm^2 Vented Tissue Treated Flasks	Corning	430641U
0.05% Trypsin-EDTA Phenol Red	Gibco	25300054
96-well Solid White Flat Bottom Polystyrene Tissue Culture Treated Microplates	Corning	3917
GCG(1-29)-OH	Anaspec	AS-22456
PTH(1-35)-(Lys35-tetramethylrhodamine)-NH_2	In house previously	Reference[3]
PathHunter® eXpress GCGR Total GPCR Internalization Assay	Eurofins DiscoverX Products Company	98-0820E1CP0M
Costar 50 mL Reagent Reservoirs	DOT Scientific	4870
GFP^2 β-arrestin-2 (R393E, R395E)	In house previously	Reference[4]
GCGR-FLAG	Genscript	OHu27939D
GCGR-RLuc8	This paper	N/A
**Circular Dichroism (CD) and Proteolysis**		
2,2,2-Trifluoroethanol ReagentPlus®	Millipore Sigma	T63002
Proteinase K Lyophilized	Millipore Sigma	70663-4
Acetaminophen BioXtra	Millipore Sigma	A7085
**Softwares and Illustrations**		
GraphPad Prism 10	GraphPad Software, LLC	N/A
Microsoft PowerPoint	Microsoft	N/A
ChemDraw Prime 16.0	PerkinElmer	N/A

III: Supplementary Note

A: Supplementary Methods

Peptide synthesis of (P2-(Lys^{35}-TMR)

P2 labeled with tetramethylrhodamine (**P2**-(Lys^{35}-TMR)) was synthesized as described for general peptide synthesis in the Methods section, but with side chain Alloc-protected Lys in position 35. Before resin cleavage and global deprotection, orthogonal removal of the Alloc group was performed with one molar equivalent of tetrakis(triphenylphosphine)palladium(0) and ten equivalents of phenylsilane in 1,2-dichloromethane at room temperature for 10 min; this process of Alloc group removal was performed twice. Labeling of Lys35 with the TMR group was performed using 6-carboxytetramethylrhodamine, COMU and DIPEA (3 equivalents each) in biotech grade DMF at room temperature for 2 h, protected from light. Subsequent cleavage, purification, and storage were performed while the peptide was protected from light to preserve the tetramethylrhodamine fluorescent group.

Signaling bias analysis

The transduction coefficients ($\log(\tau/K_A)$) of each peptide at each signaling pathway was calculated according to the scale reported by Kenakin et al.[5], based on the Black and Leff operational model[6]. Transduction coefficients were calculated in Prism 10 using the below equations, where LogR = $\log(\tau/K_A)$. Differences in transduction coefficients ($\Delta\log(\tau/K_A)$ between the reference agonist (GCG-NH_2 or PTH(1-34) and test agonists at a given pathway were calculated by subtracting the reference $\log(\tau/K_A)$ from each respective test agonist $\log(\tau/K_A)$. Bias factors ($\Delta\Delta\log(\tau/K_A)$) were calculated as the difference between $\Delta\log(\tau/K_A)$ of each pathway being compared. Standard deviation was propagated from the individual standard deviations of each $\log(\tau/K_A)$ used in $\Delta\Delta\log(\tau/K_A)$ calculation.

$$
\begin{aligned}
A &= 10^{X} \\
operate_1 &= \left(\frac{\frac{A}{10^{LogKA_1}} + 1}{A \cdot 10^{LogR_1}} \right)^n \\
operate_2 &= \left(\frac{\frac{A}{10^{LogKA_2}} + 1}{A \cdot 10^{LogR_2}} \right)^n \\
operate_3 &= \left(\frac{\frac{A}{10^{LogKA_3}} + 1}{A \cdot 10^{LogR_3}} \right)^n \\
operate_4 &= \left(\frac{\frac{A}{10^{LogKA_4}} + 1}{A \cdot 10^{LogR_4}} \right)^n \\
operate_5 &= \left(\frac{\frac{A}{10^{LogKA_5}} + 1}{A \cdot 10^{LogR_5}} \right)^n \\
operate_6 &= \left(\frac{\frac{A}{10^{LogKA_6}} + 1}{A \cdot 10^{LogR_6}} \right)^n \\
operate_7 &= \left(\frac{\frac{A}{10^{LogKA_7}} + 1}{A \cdot 10^{LogR_7}} \right)^n \\
operate_8 &= \left(\frac{\frac{A}{10^{LogKA_8}} + 1}{A \cdot 10^{LogR_8}} \right)^n
\end{aligned}
$$

$$
\begin{aligned}
<A> Y &= basal + \frac{Emax - basal}{operate_1 + 1} \\
<B> Y &= basal + \frac{Emax - basal}{operate_2 + 1} \\
<C> Y &= basal + \frac{Emax - basal}{operate_3 + 1} \\
<D> Y &= basal + \frac{Emax - basal}{operate_4 + 1} \\
<E> Y &= basal + \frac{Emax - basal}{operate_5 + 1} \\
<F> Y &= basal + \frac{Emax - basal}{operate_6 + 1} \\
<G> Y &= basal + \frac{Emax - basal}{operate_7 + 1} \\
<H> Y &= basal + \frac{Emax - basal}{operate_8 + 1}
\end{aligned}
$$

Proteolysis with proteinase K

The protocol was adapted from Checco et al.[7] with some modifications. Stock solutions of 50 μg/mL proteinase K were prepared in DPBS, pH 7.4. For each proteolysis reaction, 4 μL of 1 mM DMSO stock solution of glucagon or heterochiral analogue was diluted with 76 μL of DPBS, after which 20 μL of proteinase K (5 μg/mL) was added. The final solution contained 40 μM of peptide (4% v/v DMSO) and 1 μg/mL of proteinase K. At each timepoint, a 10 μL aliquot of the solution was removed and mixed with 20 μL of the quenching solution: 49.5% H_2O/49.5% acetonitrile/1% TFA (v/v) with 40 μg/mL of acetaminophen included as an internal standard. For each reaction, a "0 min" control was prepared by mixing 2 μL of 1 mM DMSO stock of glucagon or heterochiral analogue with 48 μL of DPBS, after which 10 μL of this solution was removed and mixed with 20 μL of the quenching solution described above. A 10 μL aliquot of each quenched reaction solution was injected onto a Waters Acquity H-Class UPLC equipped with an Acquity BEH C18 column. A 10-60% B (A: Nanopure water+0.1% TFA; B: HPLC-grade acetonitrile (MeCN)+0.1% TFA) gradient over 10 min with a 0.3 mL/min flow rate was used for elution. The relative amount of peptide was quantified by integration of the GCG-NH_2/analogue peak at 220 nm and normalization to the acetaminophen internal standard. For each sample, the % peptide remaining was calculated by dividing the normalized peak area by the normalized peak area from the "0 min" control. Each reaction was run in triplicate. Half-life values were determined by fitting normalized data to a one-phase decay model in GraphPad Prism 10.

For proteolysis of PTH(1-34) and **P2**, stock solutions of 5 μg/mL proteinase K from *Tritirachium album* were prepared in 1X TBS, pH 7.4, with 1 mM $CaCl_2$. For each proteolysis reaction, 44 μL of 100 μM peptide was diluted with 33 μL of Nanopure water and 11 μL of 10X TBS, to which 22 μL of proteinase K (5 μg/mL) was added. The final mixture consisted of 40 μM peptide and 1 μg/mL proteinase K, and the reaction was allowed to proceed at room temperature. At each timepoint, 10 μL of reaction solution was removed and mixed with 20 μL of a quenching solution: 99% (v/v) Nanopure water /1% (v/v) TFA with 40 μg/mL of acetaminophen included as an internal standard. A "0 min" timepoint was prepared by diluting 4 μL of 100 μM peptide with 6 μL of Nanopure water, after which the solution was mixed with 20 μL of the quenching solution described above.

Quenched timepoint solutions were filtered through 0.22 μm centrifuge tube filters and injected onto a Waters Acquity UPLC H-Class instrument equipped with a Waters UPLC BEH C18 column and eluted with a 10-60% (A: Nanopure water+0.1% TFA; B: HPLC-grade acetonitrile(MeCN)+0.1% TFA) gradient over 6 min using 0.45 mL/min flow rate. The relative amount of peptide was quantified by integration of the intact peptide peak at 210 nm and normalization to the acetaminophen internal standard. For each reaction, the percent peptide remaining for each timepoint was calculated by dividing its normalized peak area by the normalized peak area at the "0 min" timepoint. Each proteolysis reaction was run in triplicate. Half-life values were determined by fitting the time course of peptide degradation to a one-phase decay model using GraphPad Prism 10.

Proteolysis with human serum

Stock solutions of 25% human serum from Male AB Human Serum were prepared by diluting serum in sterile Nanopure water and stored at –20 °C until further use. Immediately before use, 25% human serum stock was allowed to thaw at room temperature, after which it was pre-warmed to 37 °C for 30 min before beginning the assay. For each proteolysis reaction, a 5 mM stock of each peptide dissolved in DMSO was

diluted to 1 mM with DPBS. 20 µL of 1 mM solution of glucagon or heterochiral analogue was then mixed with 180 µL of pre-warmed 25% human serum, and this mixture was incubated at 37 °C. The final solution contained 100 µM peptide (2% v/v DMSO) in 22.5% v/v of human serum. At each timepoint, a 20 µL aliquot of the reaction was removed and mixed with 20 µL of the quenching solution: 42.5% H_2O/42.5% acetonitrile/15% trichloroacetic acid (TCA) with 40 µg/mL of glibenclamide included as an internal standard. For each reaction, a "0 min" control was prepared by aliquoting 20 µL from the proteolysis solution after vigorously mixing it for 10 sec. After quenching the reaction, the aliquots were incubated at 4 °C for 40 min. Individual aliquots were then centrifuged to separate precipitated serum proteins. 20 µL of the supernatant was then mixed with 13.5 µL of 1 M NaOH to adjust pH to 7, followed by a second round of centrifugation to remove any precipitate that formed. A 10 µL aliquot of each quenched and neutralized reaction solution was injected onto a Waters Acquity H-Class UPLC equipped with an Acquity BEH C18 column. A 5-90% B (A: Nanopure water+0.1% TFA; B: HPLC-grade acetonitrile (MeCN)+0.1% TFA) gradient over 10 min with a 0.3 mL/min flow rate was used for elution. The relative amount of peptide was quantified by integration of the GCG-NH_2/analogue peak at 220 nm and normalization to the glibenclamide internal standard. For each sample, the % peptide remaining was calculated by dividing the normalized peak area by the normalized peak area from the "0 min" control. Each reaction was run in triplicate. Half-life values were determined by fitting normalized data to a one-phase decay model in GraphPad Prism 10.

NLuc-PTH1R kinetic direct binding for P2

Protocol was adapted from Yu et al.[1] with slight modifications. HEK293 cells stably expressing NLuc-PTH1R were seeded on a black, clear-bottomed 96-well plate at 10,000 cells/well. After incubation at 37 °C for 24 h, the medium was aspirated, and each well was rinsed with 50 µL of DPBS. 80 µL of DPBS supplemented with 1 mM $CaCl_2$, 0.5 mM $MgCl_2$, 0.1% BSA and 0.02% NaN_3 was added, and the plate was allowed to incubate at room temperature for 30 min. **P2**-(Lys^{35}-TMR solutions at various concentrations were prepared from the above DPBS solution supplemented with 5 µM furimazine substrate. The medium was aspirated from wells, 100 µL of peptide solution was added, and the luminescence at 460 and 610 nm was immediately read using a BioTek Synergy Neo2 plate reader. The milli BRET (mBRET) values were calculated as the ratio of the luminescence at 610 nm over luminescence at 450 nm with a 1000× multiplier, with the mBRET value from vehicle wells subtracted. The mBRET values were plotted against time and fitted to a one-phase association (Eq 1) to calculate k_{obs}. k_{obs} were plotted against peptide concentration and fitted to a linear regression, according to Eq 2, to calculate k_{on} and k_{off}. K_D was calculated as the ratio of k_{off} to k_{on} (Eq 3).

Equation 1: $Y = Y_{t(\mathrm{inf})} \times (1 - e^{-k_{\mathrm{obs}}\mathrm{t}})$

Equation 2: $k_{\mathrm{obs}} = k_{\mathrm{on}}[\mathrm{L}] + k_{\mathrm{off}}$

Equation 3: $K_{\mathrm{D}} = k_{\mathrm{off}}/k_{\mathrm{on}}$

B: Plasmid sequences

GCGR-RLuc8 plasmid DNA sequence and translated protein sequence

Length: 2413 bp
Vector: pcDNA3.1(+)
Cloning sites: NheI-HindIII

gct agc = NheI
aag ctt = HindIII
gcc acc = Kozak sequence
Linkers = **black**
Signal peptide = orange
RLuc8 = purple
GCGR = brown

```
gcta gcc acc atg ccc cca tgc cag ccc cag agg ccc ctg ctg ctg ctg ctc ctg ctg ctg
              M   P   P   C   Q   P   Q   R   P   L   L   L   L   L   L   L   L
gcc tgc cag cct cag gtg ccc agc gct cag gtc atg gat ttc ctg ttc gag aag tgg aag
 A   C   Q   P   Q   V   P   S   A   Q   V   M   D   F   L   F   E   K   W   K
ctg tac ggc gac cag tgc cac cac aat ctg agc ctg ctg ccc ccc cct aca gag ctg gtg
 L   Y   G   D   Q   C   H   H   N   L   S   L   L   P   P   P   T   E   L   V
tgc aat agg aca ttc gac aag tac agc tgc tgg ccc gat acc cct gcc aat acc aca gcc
 C   N   R   T   F   D   K   Y   S   C   W   P   D   T   P   A   N   T   T   A
aac atc tcc tgt ccc tgg tac ctg ccc tgg cac cac aag gtg cag cac aga ttc gtg ttc
 N   I   S   C   P   W   Y   L   P   W   H   H   K   V   Q   H   R   F   V   F
aag aga tgt ggc ccc gac ggc cag tgg gtg aga ggc cct agg ggc cag cct tgg aga gac
 K   R   C   G   P   D   G   Q   W   V   R   G   P   R   G   Q   P   W   R   D
gcc agc cag tgt cag atg gac ggc gag gag atc gag gtg cag aag gag gtg gcc aag atg
 A   S   Q   C   Q   M   D   G   E   E   I   E   V   Q   K   E   V   A   K   M
tac tcc tcc ttt cag gtc atg tac acc gtg ggc tac tcc ctg agc ctg ggc gcc ctg ctg
 Y   S   S   F   Q   V   M   Y   T   V   G   Y   S   L   S   L   G   A   L   L
ctg gct ctg gct atc ctg ggc ggc ctg agc aag ctg cac tgc acc aga aac gcc atc cac
 L   A   L   A   I   L   G   G   L   S   K   L   H   C   T   R   N   A   I   H
gcc aac ctg ttc gcc tcc ttt gtg ctg aag gcc tcc tcc gtg ctg gtc att gat ggc ctg
 A   N   L   F   A   S   F   V   L   K   A   S   S   V   L   V   I   D   G   L
ctg agg aca aga tac agc cag aag atc ggc gac gat ctg agc gtg agc acc tgg ctg tcc
 L   R   T   R   Y   S   Q   K   I   G   D   D   L   S   V   S   T   W   L   S
gac ggc gcc gtg gct gga tgt agg gtg gcc gct gtg ttc atg cag tac ggc atc gtg gcc
 D   G   A   V   A   G   C   R   V   A   A   V   F   M   Q   Y   G   I   V   A
aat tac tgc tgg ctg ctg gtg gag ggc ctg tac ctg cac aat ctg ctg ggc ctg gcc acc
 N   Y   C   W   L   L   V   E   G   L   Y   L   H   N   L   L   G   L   A   T
ctg cct gag aga tcc ttc ttt agc ctg tac ctg ggc atc ggc tgg ggc gcc cct atg ctg
 L   P   E   R   S   F   F   S   L   Y   L   G   I   G   W   G   A   P   M   L
ttt gtg gtg cct tgg gcc gtg gtg aag tgc ctg ttt gag aac gtg cag tgc tgg aca tcc
 F   V   V   P   W   A   V   V   K   C   L   F   E   N   V   Q   C   W   T   S
aac gat aat atg ggc ttt tgg tgg atc ctg aga ttc ccc gtg ttc ctg gcc atc ctg atc
 N   D   N   M   G   F   W   W   I   L   R   F   P   V   F   L   A   I   L   I
aac ttt ttc atc ttt gtg agg atc gtg cag ctg ctg gtg gcc aag ctg aga gcc agg cag
 N   F   F   I   F   V   R   I   V   Q   L   L   V   A   K   L   R   A   R   Q
atg cac cac aca gac tac aag ttc aga ctg gcc aag tcc acc ctg acc ctg atc cct ctg
 M   H   H   T   D   Y   K   F   R   L   A   K   S   T   L   T   L   I   P   L
ctg ggc gtg cac agag gtg gtg ttt gcc ttt gtg aca gat gag cac gcc cag ggc acc ctg
 L   G   V   H   E   V   V   F   A   F   V   T   D   E   H   A   Q   G   T   L
aga agc gcc aag ctg ttt ttc gac ctg ttt ctg tcc tcc ttt caa ggc ctg ctg gtg gct
 R   S   A   K   L   F   F   D   L   F   L   S   S   F   Q   G   L   L   V   A
```

```
gtg ctg tac tgc ttt ctg aat aag gag gtg cag tcc gag ctg agg agg agg tgg cac aga
 V   L   Y   C   F   L   N   K   E   V   Q   S   E   L   R   R   R   W   H   R
tgg aga ctg ggc aag gtg ctg tgg gag gag aga aat acc agc aac cac aga gcc agc agc
 W   R   L   G   K   V   L   W   E   E   R   N   T   S   N   H   R   A   S   S
tcc ccc ggc cac gga cct cct agc aag gag ctg cag ttc ggc agg ggc ggc ggc tct cag
 S   P   G   H   G   P   P   S   K   E   L   Q   F   G   R   G   G   G   S   Q
gac agc tct gcc gag acc cct ctg gcc ggc gga ctg cct aga ctg gcc gag tcc cct ttc
 D   S   S   A   E   T   P   L   A   G   G   L   P   R   L   A   E   S   P   F
ggc ggc ggc gga tct ggc gga gga gga tct atg gcc agc aag gtg tac gat ccc gag cag
 G   G   G   G   S   G   G   G   G   S   M   A   S   K   V   Y   D   P   E   Q
aga aag agg atg atc acc ggc cct cag tgg tgg gcc agg tgc aag cag atg aac gtg ctg
 R   K   R   M   I   T   G   P   Q   W   W   A   R   C   K   Q   M   N   V   L
gat tcc ttc atc aat tac tac gac agc gag aag cac gcc gag aac gcc gtg atc ttt ctg
 D   S   F   I   N   Y   Y   D   S   E   K   H   A   E   N   A   V   I   F   L
cac ggc aat gcc aca tcc tcc tac ctg tgg aga cac gtg gtg cct cac atc gag ccc gtg
 H   G   N   A   T   S   S   Y   L   W   R   H   V   V   P   H   I   E   P   V
gcc agg tgc atc atc cct gac ctg atc ggc atg ggc aag tcc ggc aag agc ggc aat ggc
 A   R   C   I   I   P   D   L   I   G   M   G   K   S   G   K   S   G   N   G
agc tac aga ctg ctg gat cac tac aag tac ctg aca gcc tgg ttc gag ctg ctg aac ctg
 S   Y   R   L   L   D   H   Y   K   Y   L   T   A   W   F   E   L   L   N   L
cct aag aag atc atc ttc gtg ggc cac gac tgg ggc gcc gcc ctg gct ttt cac tac gcc
 P   K   K   I   a   F   V   G   H   D   W   G   A   A   L   A   F   H   Y   A
tac gag cac cag gac aga atc aag gcc atc gtg cac atg gag agc gtg gtg gac gtg atc
 Y   E   H   Q   D   R   I   K   A   I   V   H   M   E   S   V   V   D   V   I
gag agc tgg gac gag tgg cct gat atc gag gag gac atc gcc ctg atc aag agc gag gag
 E   S   W   D   E   W   P   D   I   E   E   D   I   A   L   I   K   S   E   E
ggc gag aag atg gtg ctg gag aat aac ttt ttc gtg gag aca gtg ctg ccc agc aag atc
 G   E   K   M   V   L   E   N   N   F   F   V   E   T   V   L   P   S   K   I
atg aga aag ctg gag cct gag gag ttc gcc gcc tac ctg gag cct ttt aag gag aag ggc
 M   R   K   L   E   P   E   E   F   A   A   Y   L   E   P   F   K   E   K   G
gag gtg agg agg ccc acc ctg agc tgg cct aga gag atc cct ctg gtg aag ggc ggc aag
 E   V   R   R   P   T   L   S   W   P   R   E   I   P   L   V   K   G   G   K
cct gac gtg gtg cag atc gtg aga aac tac aac gcc tac ctg agg gcc agc gac gat ctg
 P   D   V   V   Q   I   V   R   N   Y   N   A   Y   L   R   A   S   D   D   L
ccc aag ctg ttt atc gag agc gac ccc ggc ttt ttc tcc aat gcc atc gtg gag ggc gcc
 P   K   L   F   I   E   S   D   P   G   F   F   S   N   A   I   V   E   G   A
aag aag ttc cct aat aca gag ttt gtg aag gtg aag ggc ctg cac ttc ctg cag gag gat
 K   K   F   P   N   T   E   F   V   K   V   K   G   L   H   F   L   Q   E   D
gcc ccc gat gag atg ggc aag tac atc aag agc ttt gtg gag agg gtg ctg aag aac gag
 A   P   D   E   M   G   K   Y   I   K   S   F   V   E   R   V   L   K   N   E
cag tga aag ctt
 Q   -
```

C: Instrumentation and Instrumentation Acknowledgments

The purchase of the Bruker microflex LRF MALDI-TOF-MS in 2015 was funded by the Bender gift to the Department of Chemistry. This instrument was used for peptide characterization.

Bruker Impact II (ESI-Q-TOF-MS) mass spectrometer with an UltiMate 3000 HPLC, was made possible by the Bender legacy gift to the Department of Chemistry. This instrument was used for peptide characterization.

The purchase of the JASCO J-1500 CD spectrometer was funded by NIH R01 GM061238. This instrument was used for analysis of the peptide α-helicity.

The purchase of the Waters Acquity H-Class UPLC was supported by DARPA N66001-15-2-4023. This instrument was used for proteolysis measurements and peptide purity analysis.

A ThermoScientfic NanoDrop 200 was used for peptide concentration measurements.

A BioTek Synergy 2 Microplate Reader and BioTek Synergy Neo2 Hybrid Multimode Reader (purchased with funds provided by the UW-Madison Vilas Trust) were used for all cell-based experiments.

An Agilent 1260 Infinity II reverse-phase HPLC equipped with a Waters or Supelco C18 column was used for purification of peptides.

D: Peptide Characterization and Purity

GCG-OH

This peptide was available from a commercial vendor (see Supplementary Table 10).

GCG-NH$_2$

MALDI-TOF-MS: Calculated monoisotopic [M+H]$^+$ =3480.639; Observed monoisotopic M+H]$^+$ =3480.967

Analytical RP-HPLC (ESI-MS): H_2O/MeCN + 0.1% FA, 45-95% MeCN, 10 min, 0.3 mL/min; Agilent PLRP-S 300Å (3 µm, 2.1 x 50 mm) column.

Calculated monoisotopic [M+3H]$^{+3}$ = 1160.8850; [M+4H]$^{+4}$ = 870.9157; [M+5H]$^{+5}$ = 696.9341
Observed monoisotopic [M+3H]$^{+3}$ = 1161.5530; [M+4H]$^{+4}$ = 870.9156; [M+5H]$^{+5}$ = 696.9344

UPLC (220 nm): H_2O/MeCN + 0.1% TFA, 10-90% MeCN 0-10 min, wash and column equilibration 10-16 min, 0.3 mL/min; CSH C18 (1.7 µm, 2.1 x 150 mm) column.

Purity: 97.49%

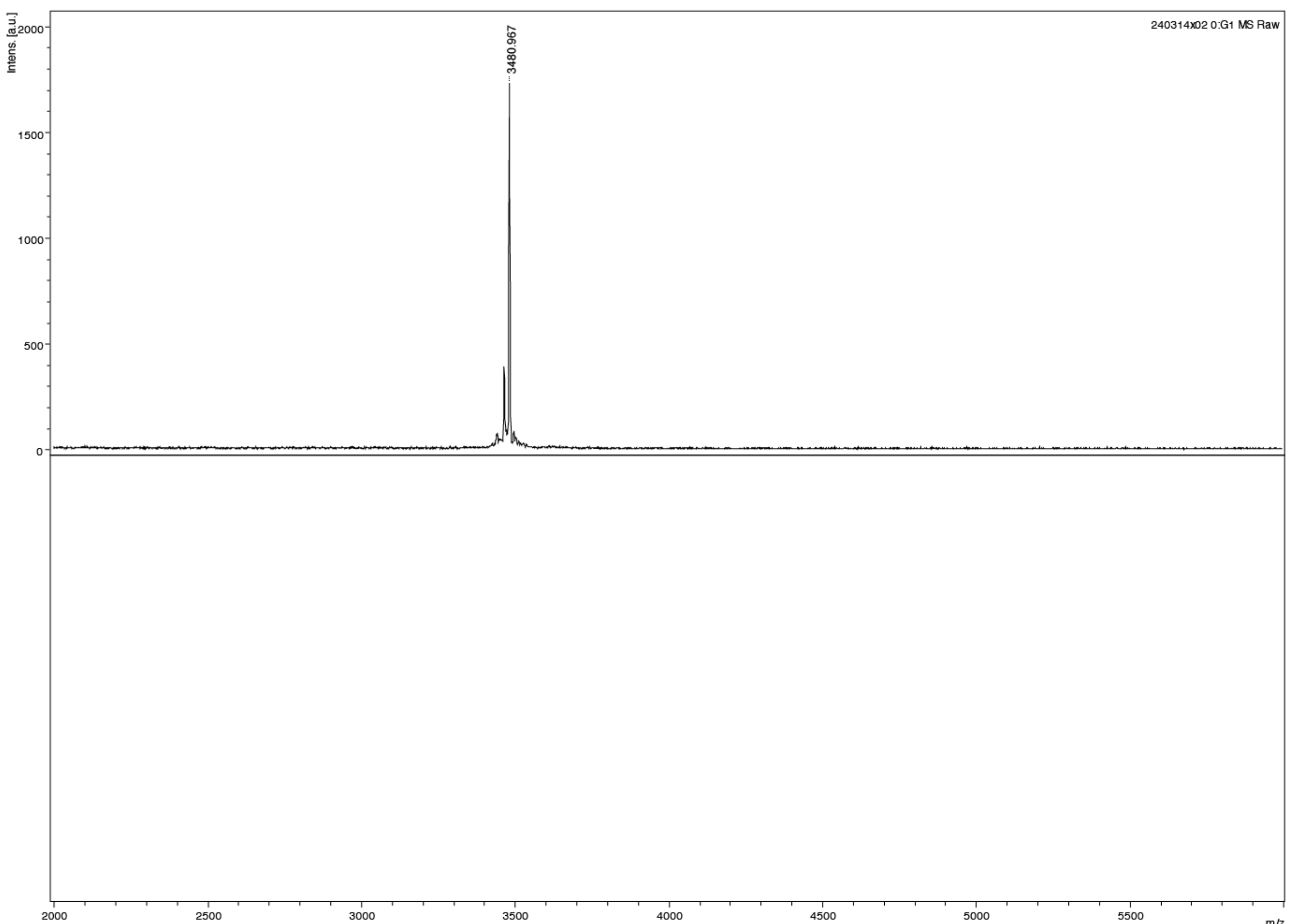

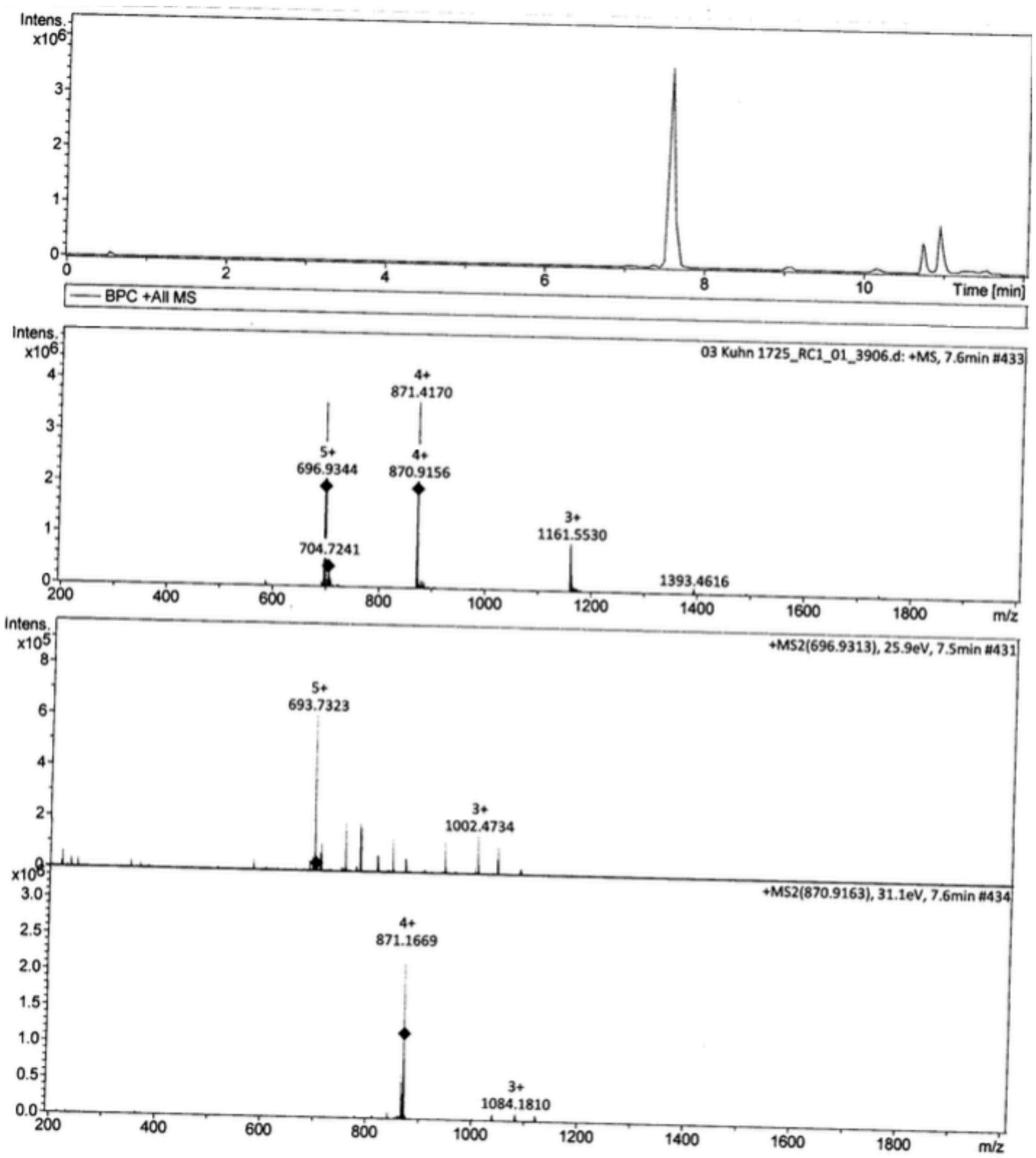

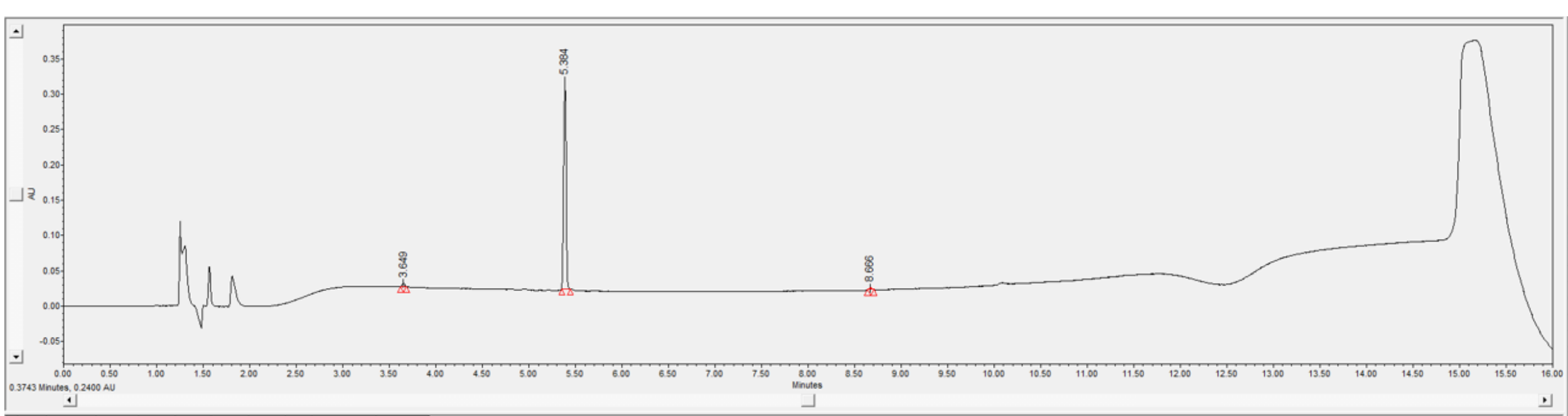

	Name	Retention Time (min)	Area (µV*sec)	% Area	Height (µV)	Int Type	Amount	Units	Peak Type	Peak Codes
1		3.649	8922	1.57	4863	bb			Unknown	
2		5.384	554478	97.49	295615	bb			Unknown	
3		8.666	5341	0.94	2471	bb			Unknown	

G1

MALDI-TOF-MS: Calculated monoisotopic $[M+H]^{+}$ =3480.639; Observed monoisotopic $M+H]^{+}$ =3480.698

Analytical RP-HPLC (ESI-MS): H_2O/MeCN + 0.1% FA, 45-95% MeCN, 10 min, 0.3 mL/min; Agilent PLRP-S 300Å (3 µm, 2.1 x 50 mm) column.

Calculated monoisotopic $[M+2H]^{+2}$ = 1740.8236; $[M+3H]^{+3}$ = 1160.8850; $[M+4H]^{+4}$ = 870.9157; $[M+5H]^{+5}$ = 696.9341
Observed monoisotopic $[M+2H]^{+2}$ = 1741.8268; $[M+3H]^{+3}$ = 1161.5534; $[M+4H]^{+4}$ = 870.9158; $[M+5H]^{+5}$ = 696.9345

UPLC (220 nm): H_2O/MeCN + 0.1% TFA, 10-90% MeCN 0-10 min, wash and column equilibration 10-16 min, 0.3 mL/min; CSH C18 (1.7 µm, 2.1 x 150 mm) column.

Purity: 97.04%

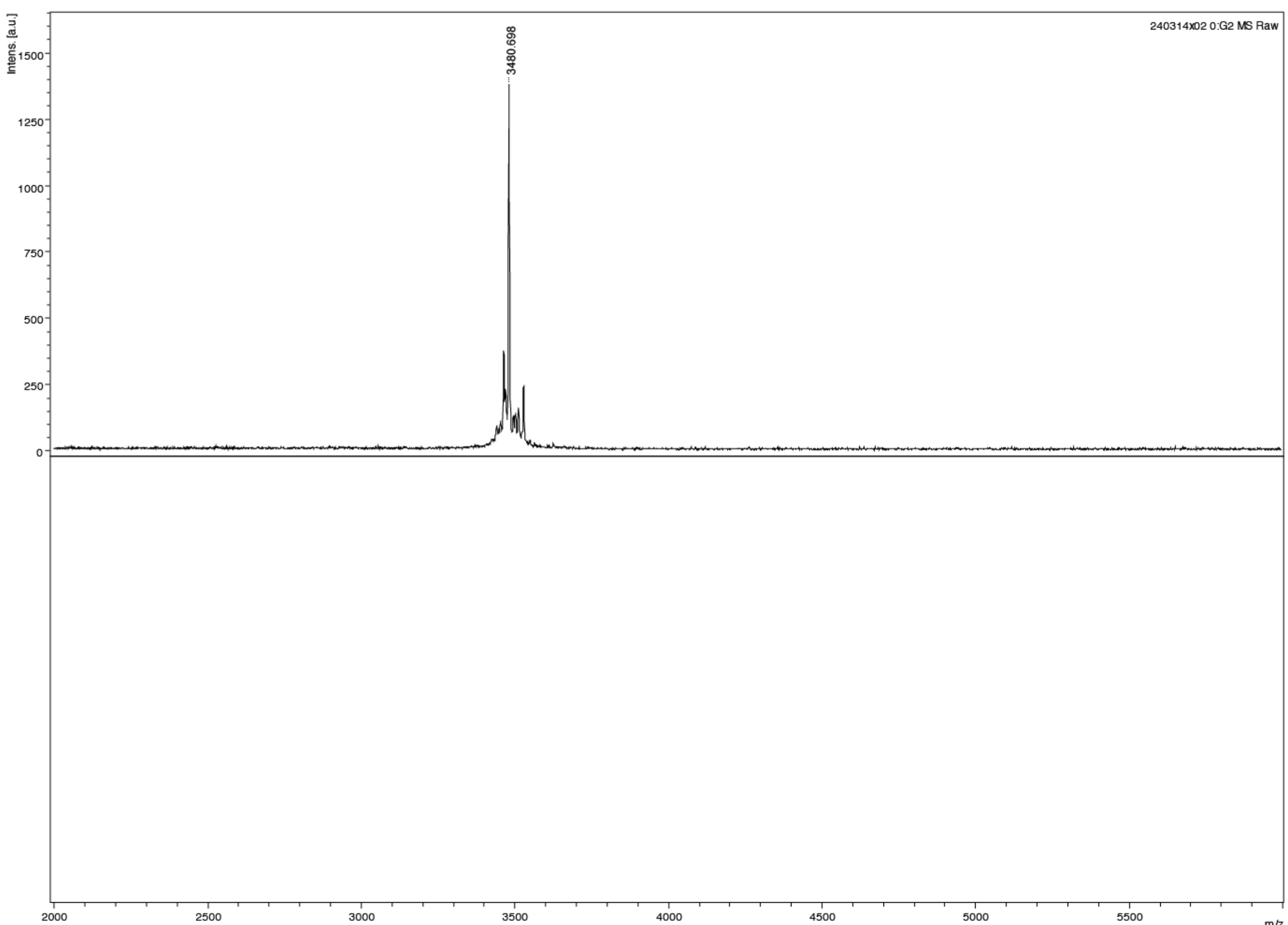

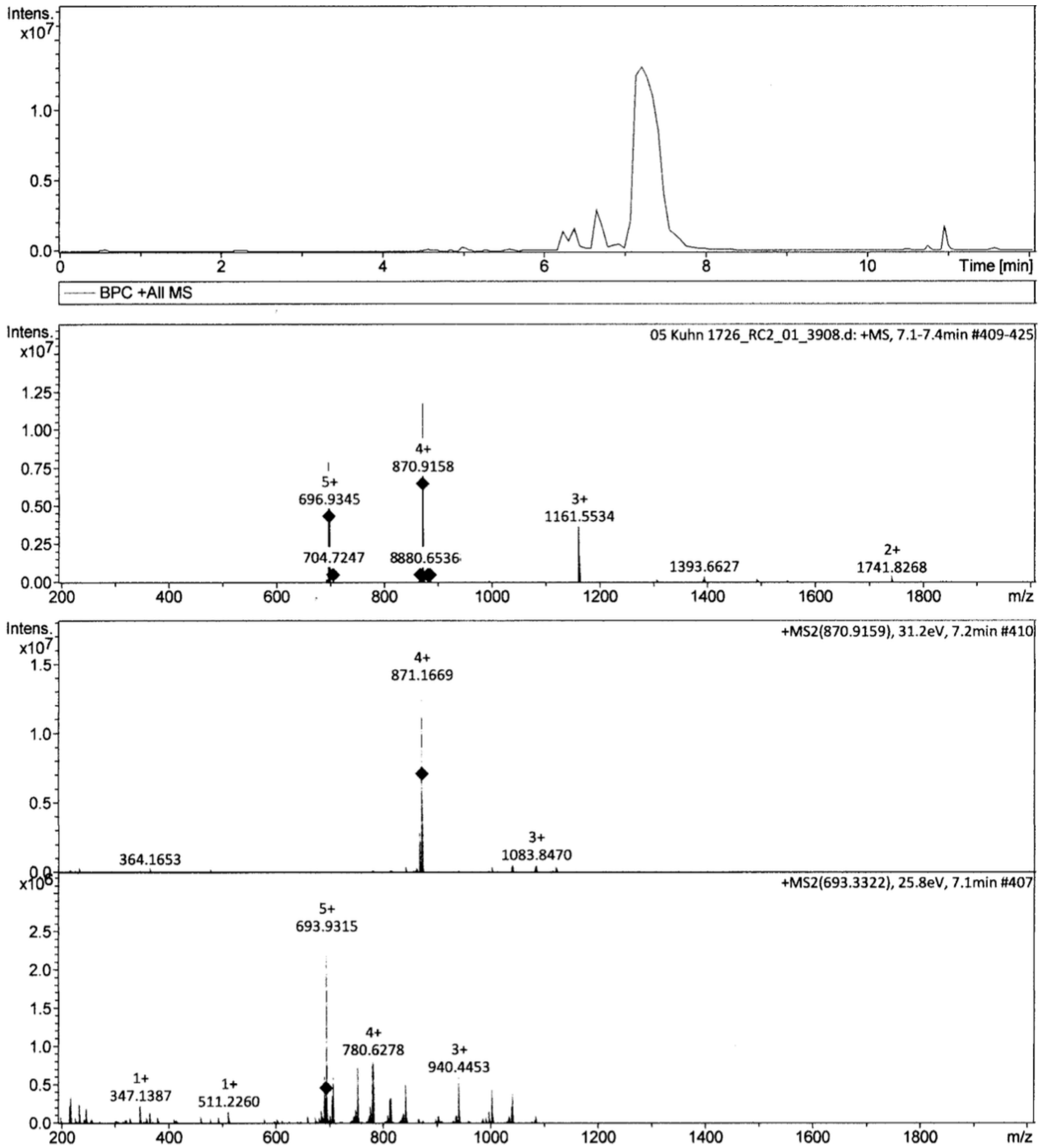

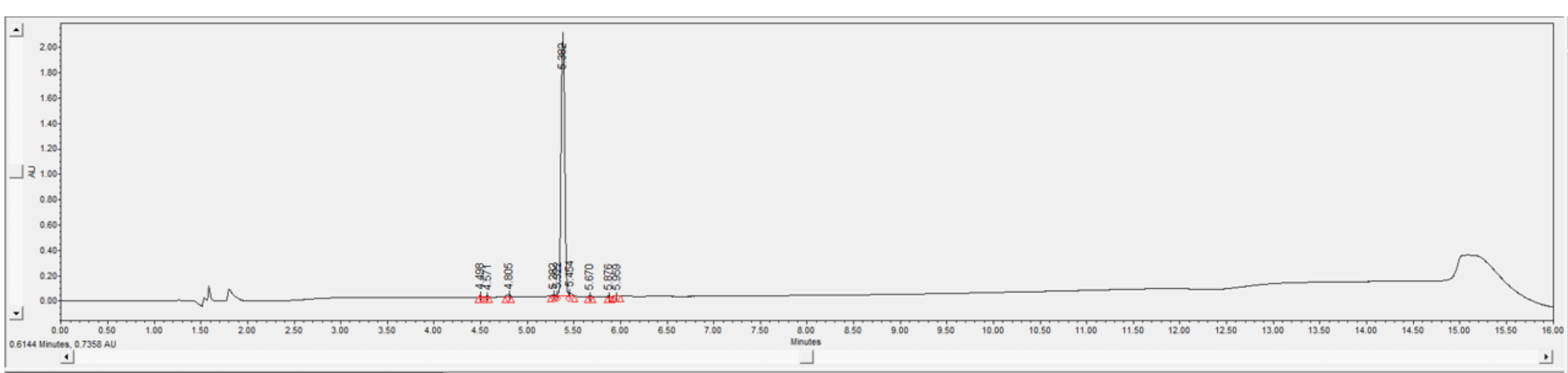

	Name	Retention Time (min)	Area (µV*sec)	% Area	Height (µV)	Int Type	Amount	Units	Peak Type	Peak Codes
1		4.498	19755	0.37	11365	bb			Unknown	
2		4.571	11553	0.22	6873	bb			Unknown	
3		4.805	34383	0.65	12191	bb			Unknown	
4		5.282	10748	0.20	5863	bv			Unknown	
5		5.322	12027	0.23	11851	vv			Unknown	I06
6		5.382	5123296	97.04	2039644	vv			Unknown	
7		5.454	50886	0.96	21950	vb			Unknown	
8		5.670	7981	0.15	4024	bb			Unknown	
9		5.876	3161	0.06	2180	bb			Unknown	
10		5.959	5722	0.11	2160	bb			Unknown	

G2

MALDI-TOF-MS: Calculated monoisotopic $[M+H]^+$ =3494.655; Observed monoisotopic $M+H]^+$ =3494.282

Analytical RP-HPLC (ESI-MS): H_2O/MeCN + 0.1% FA, 45-95% MeCN, 10 min, 0.3 mL/min; Agilent PLRP-S 300Å (3 µm, 2.1 x 50 mm) column.

Calculated monoisotopic $[M+2H]^{+2}$ = 1747.8314; $[M+3H]^{+3}$ = 1165.5569; $[M+4H]^{+4}$ = 874.4196; $[M+5H]^{+5}$ = 699.7372
Observed monoisotopic $[M+2H]^{+2}$ = 1748.8334; $[M+3H]^{+3}$ = 1166.2246; $[M+4H]^{+4}$ = 874.4193; $[M+5H]^{+5}$ = 699.7373

UPLC (220 nm): H_2O/MeCN + 0.1% TFA, 10-90% MeCN 0-10 min, wash and column equilibration 10-16 min, 0.3 mL/min; CSH C18 (1.7 µm, 2.1 x 150 mm) column.

Purity: 99.13%

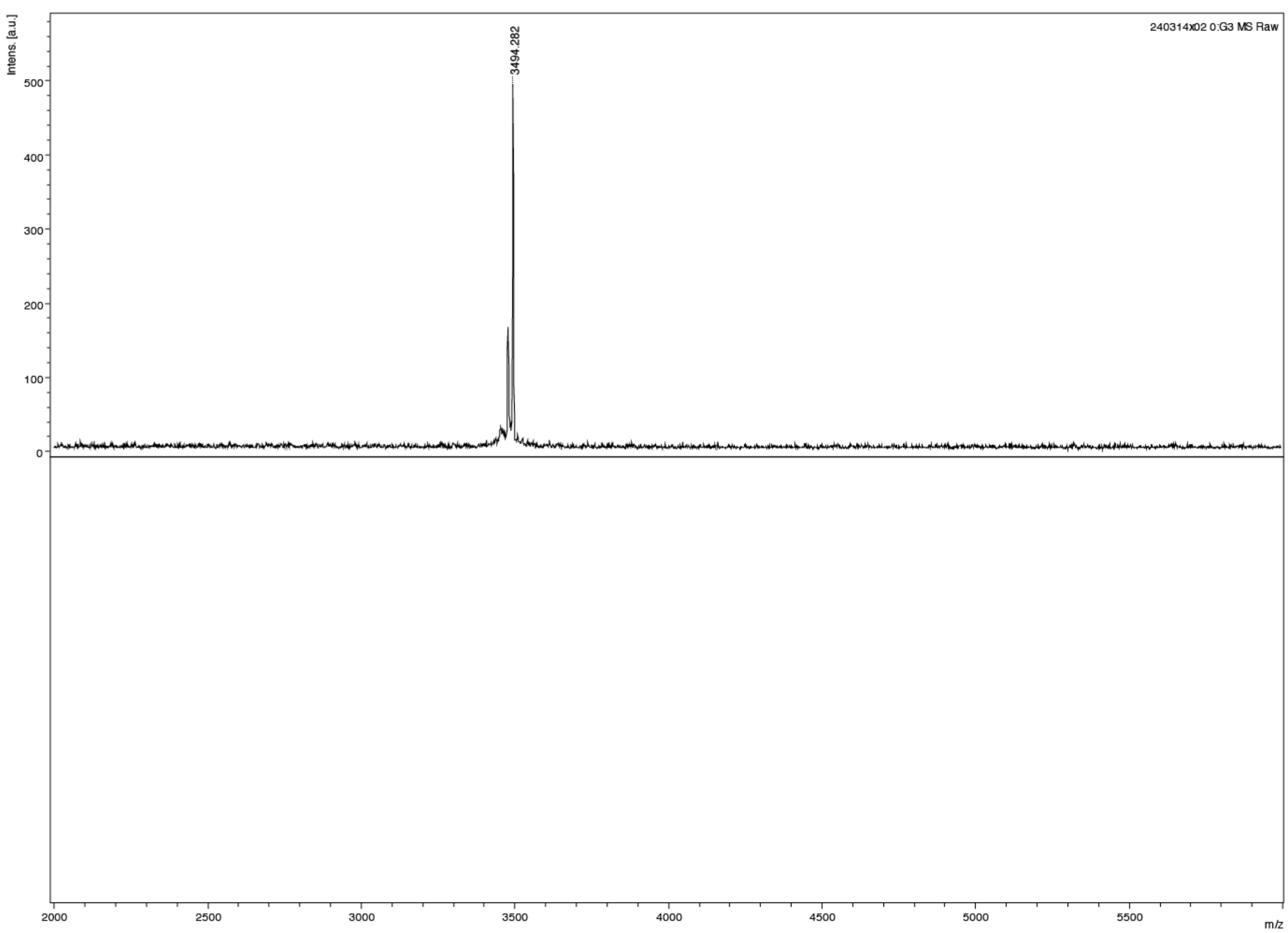

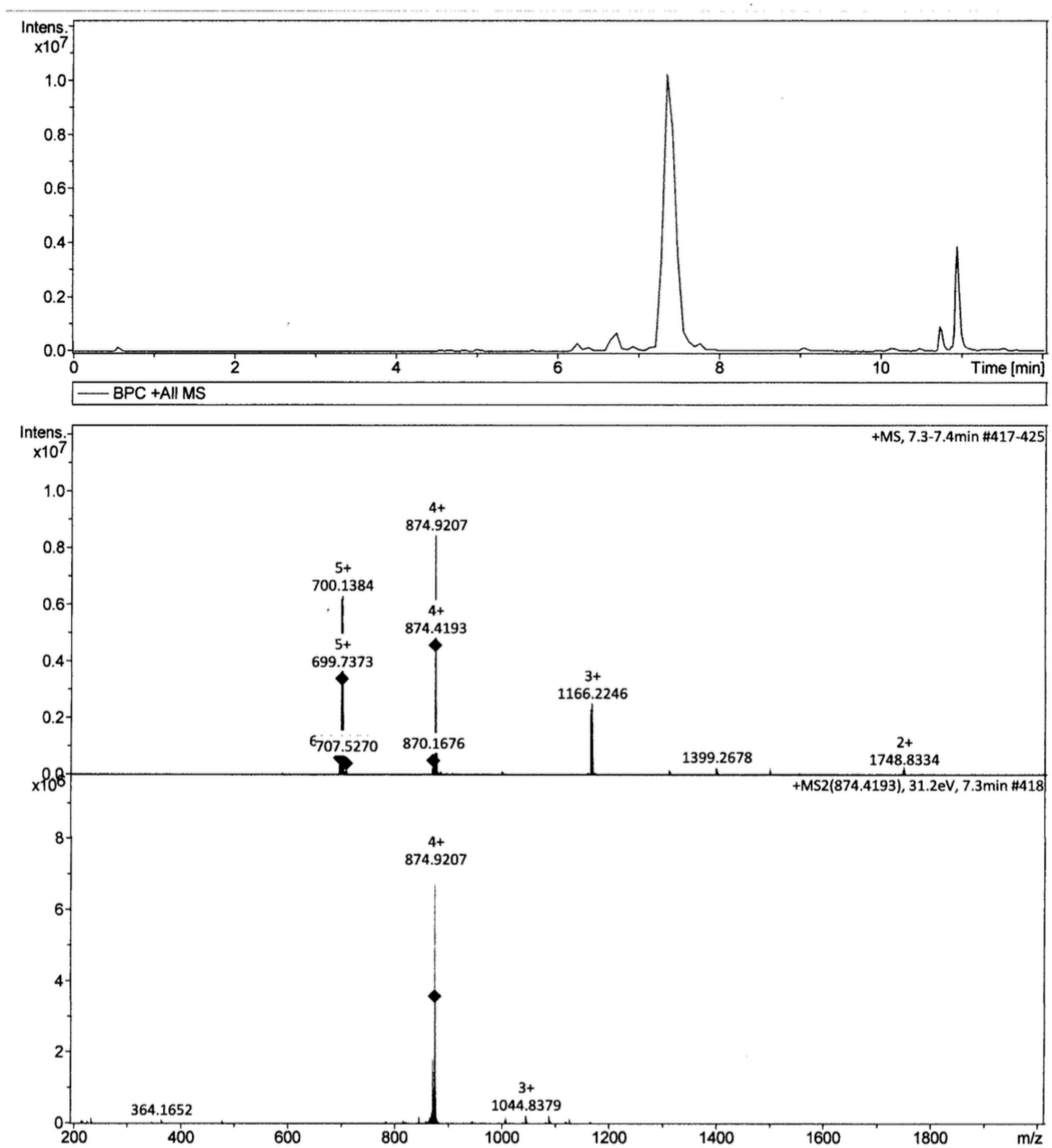

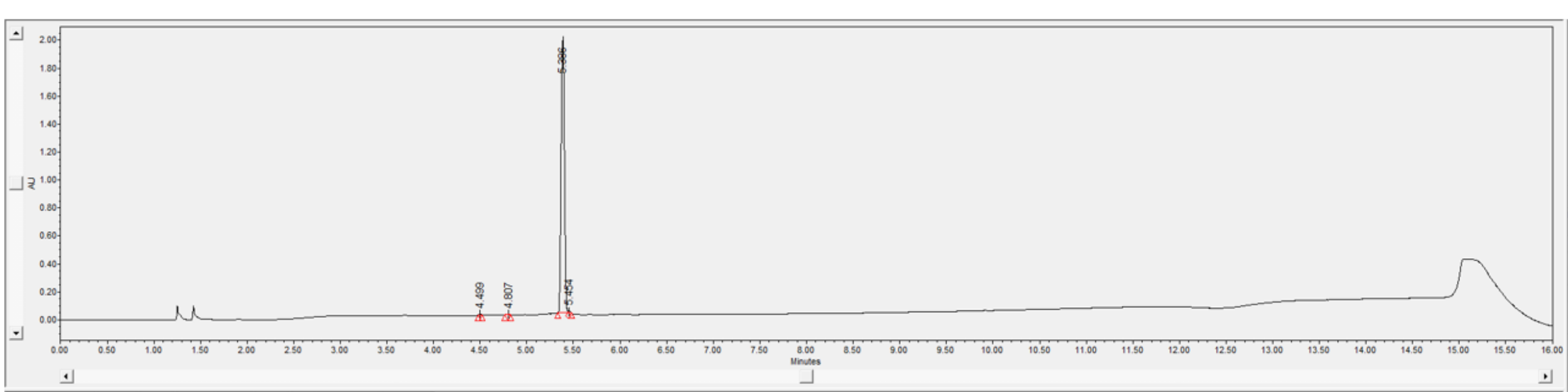

	Name	Retention Time (min)	Area (µV*sec)	% Area	Height (µV)	Int Type	Amount	Units	Peak Type	Peak Codes
1		4.499	9131	0.20	6000	bb			Unknown	
2		4.807	12137	0.27	4802	bb			Unknown	
3		5.386	4472474	99.13	1943503	bv			Unknown	
4		5.454	18128	0.40	11585	vb			Unknown	

G3

MALDI-TOF-MS: Calculated monoisotopic [M+H]$^+$ =3494.655; Observed monoisotopic M+H]$^+$ =3494.571

Analytical RP-HPLC (ESI-MS): H_2O/MeCN + 0.1% FA, 45-95% MeCN, 10 min, 0.3 mL/min; Agilent PLRP-S 300Å (3 µm, 2.1 x 50 mm) column.

Calculated monoisotopic [M+2H]$^{+2}$ = 1747.8314; [M+3H]$^{+3}$ = 1165.5569; [M+4H]$^{+4}$ = 874.4196; [M+5H]$^{+5}$ = 699.7372
Observed monoisotopic [M+2H]$^{+2}$ = 1748.8300; [M+3H]$^{+3}$ = 1166.2224; [M+4H]$^{+4}$ = 874.4177; [M+5H]$^{+5}$ = 700.1372

UPLC (220 nm): H_2O/MeCN + 0.1% TFA, 10-90% MeCN 0-10 min, wash and column equilibration 10-16 min, 0.3 mL/min; CSH C18 (1.7 µm, 2.1 x 150 mm) column.

Purity: 95.40%

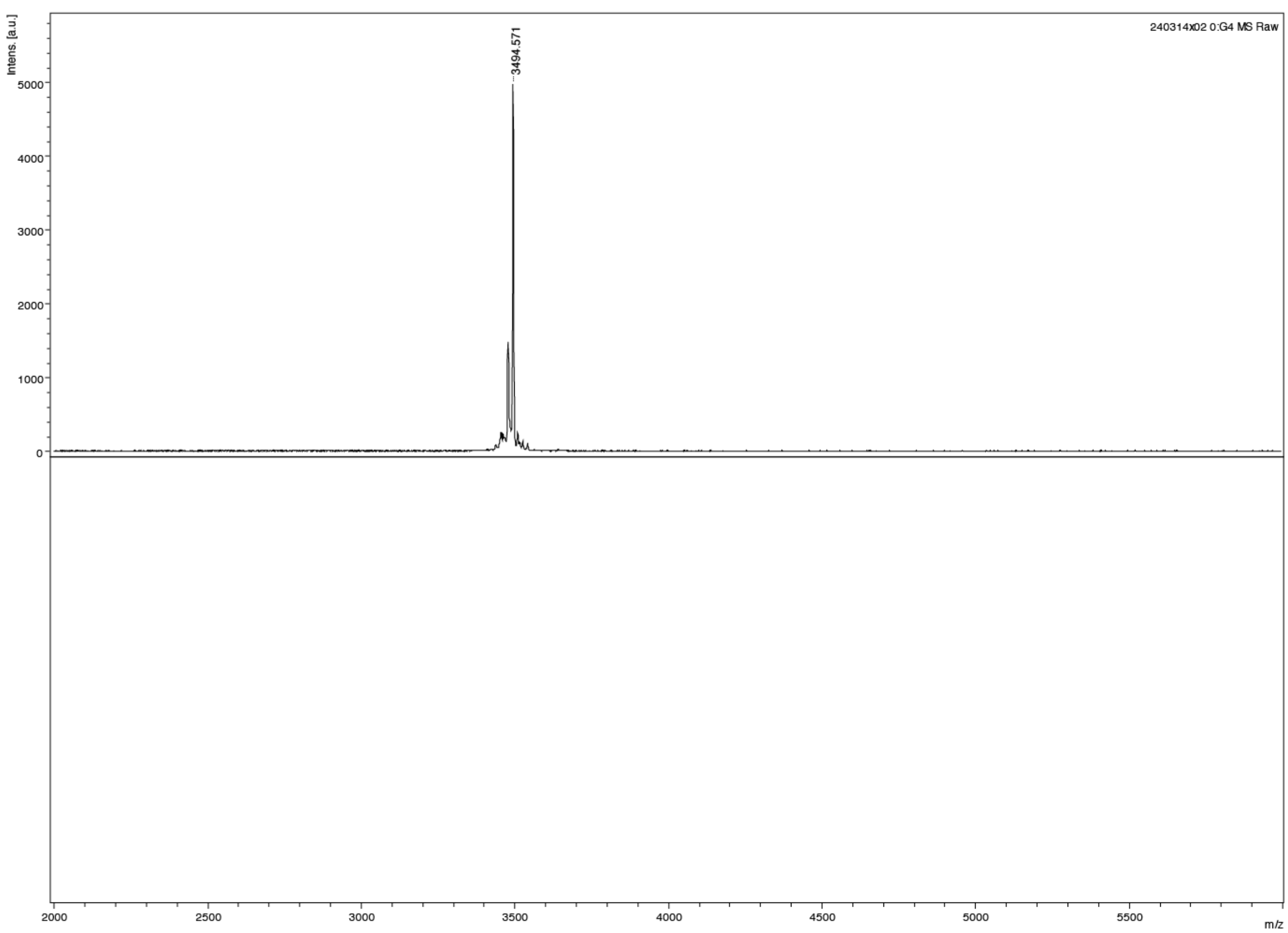

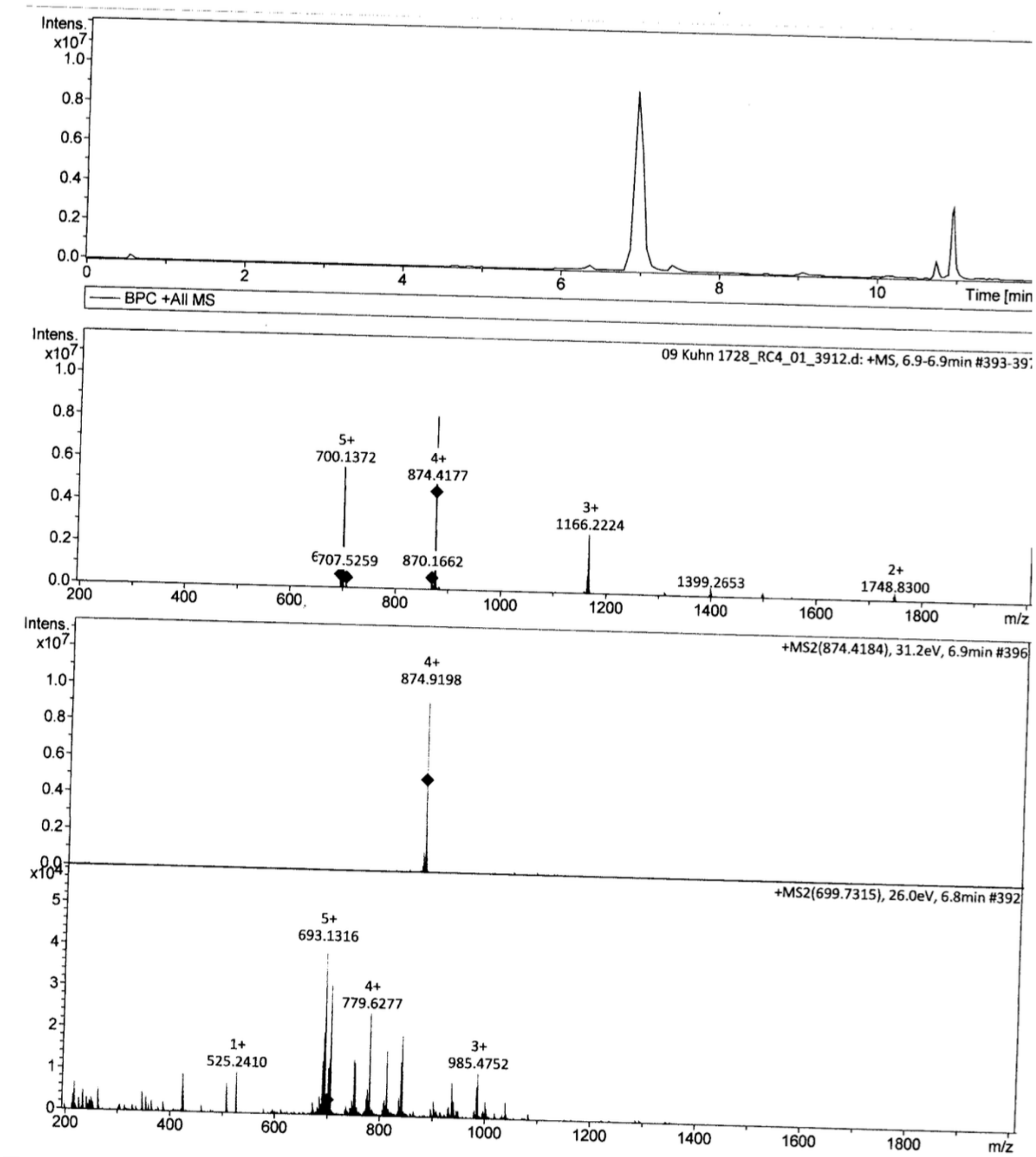

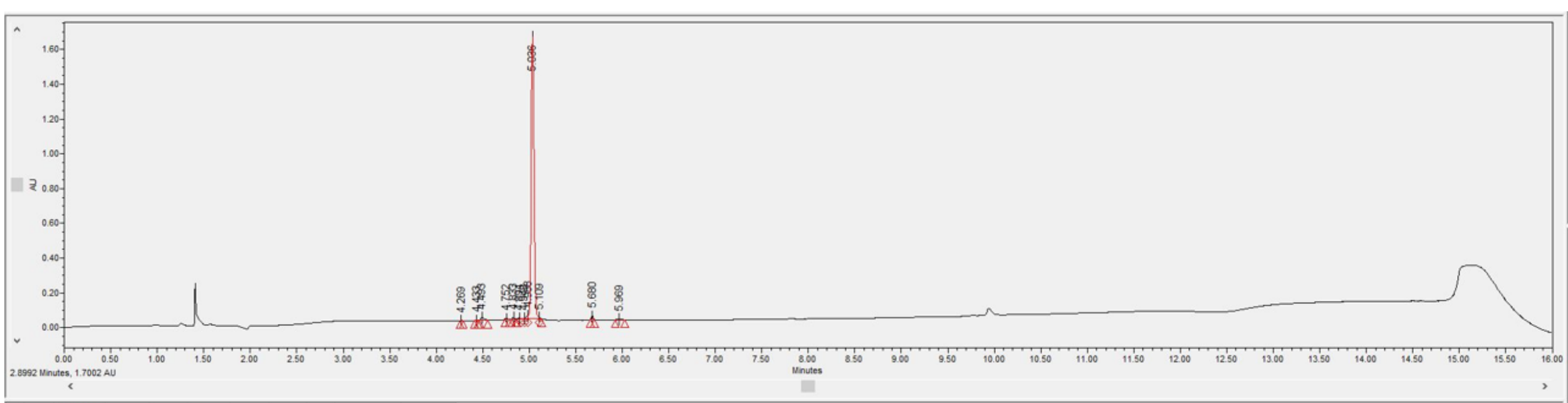

	Name	Retention Time (min)	Area (µV*sec)	% Area	Height (µV)	Int Type	Amount	Units	Peak Type	Peak Codes
1		4.269	1568	0.04	1242	bb			Unknown	
2		4.433	1866	0.05	1627	bb			Unknown	
3		4.493	35102	0.98	15899	bb			Unknown	
4		4.752	8564	0.24	5729	bb			Unknown	
5		4.833	13185	0.37	9140	bb			Unknown	
6		4.894	9417	0.26	5530	bv			Unknown	
7		4.948	9093	0.26	4872	vv			Unknown	I06
8		4.988	28591	0.80	20979	vv			Unknown	I06
9		5.036	3401113	95.40	1620973	vv			Unknown	
10		5.109	6165	0.17	8637	vb			Unknown	I06
11		5.680	31005	0.87	20805	bb			Unknown	
12		5.969	19312	0.54	5642	bb			Unknown	

G3-OH

MALDI-TOF-MS: Calculated monoisotopic $[M+H]^+$ =3495.639; Observed monoisotopic $M+H]^+$ =3495.585

Analytical RP-HPLC (ESI-MS): H_2O/MeCN + 0.1% FA, 45-95% MeCN, 10 min, 0.3 mL/min; Agilent PLRP-S 300Å (3 µm, 2.1 x 50 mm) column.

Calculated monoisotopic $[M+3H]^{+3}$ = 1165.8849; $[M+4H]^{+4}$ = 874.6656; $[M+5H]^{+5}$ = 699.9340
Observed monoisotopic $[M+3H]^{+3}$ = 1166.5504; $[M+4H]^{+4}$ = 874.6638; $[M+5H]^{+5}$ = 700.3340

UPLC (220 nm): H_2O/MeCN + 0.1% TFA, 10-90% MeCN 0-10 min, wash and column equilibration 10-16 min, 0.3 mL/min; CSH C18 (1.7 µm, 2.1 x 150 mm) column.

Purity: 92.36%

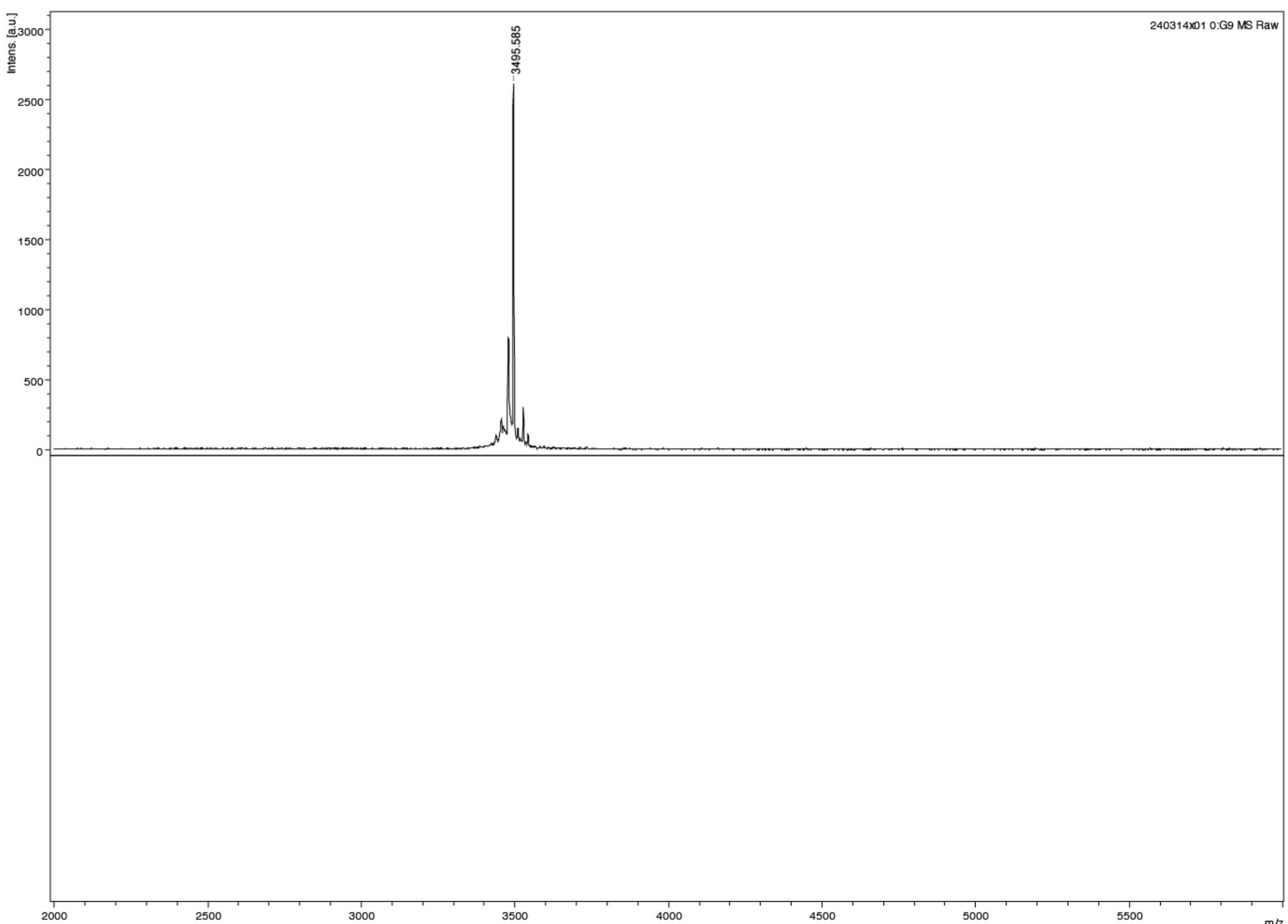

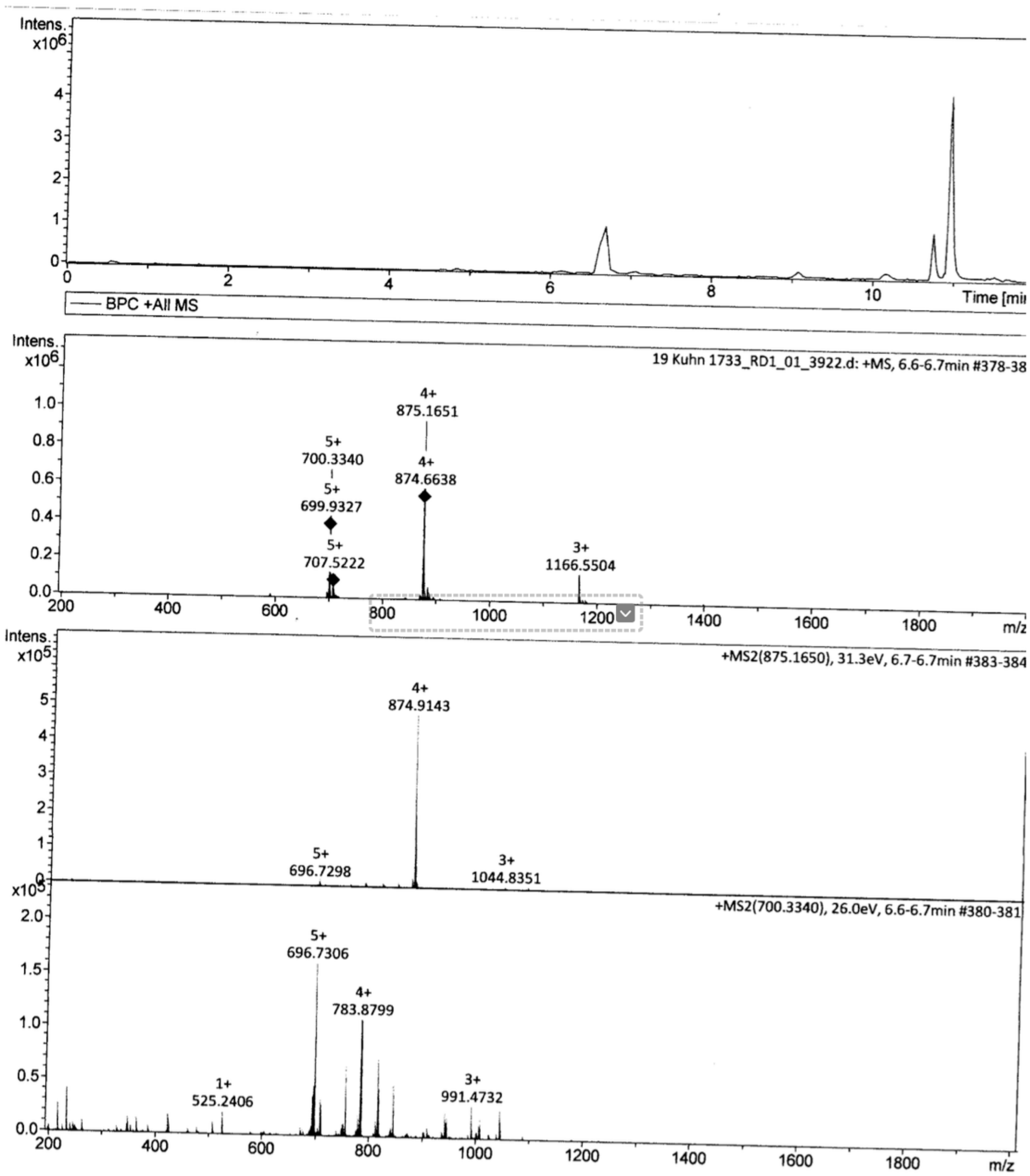

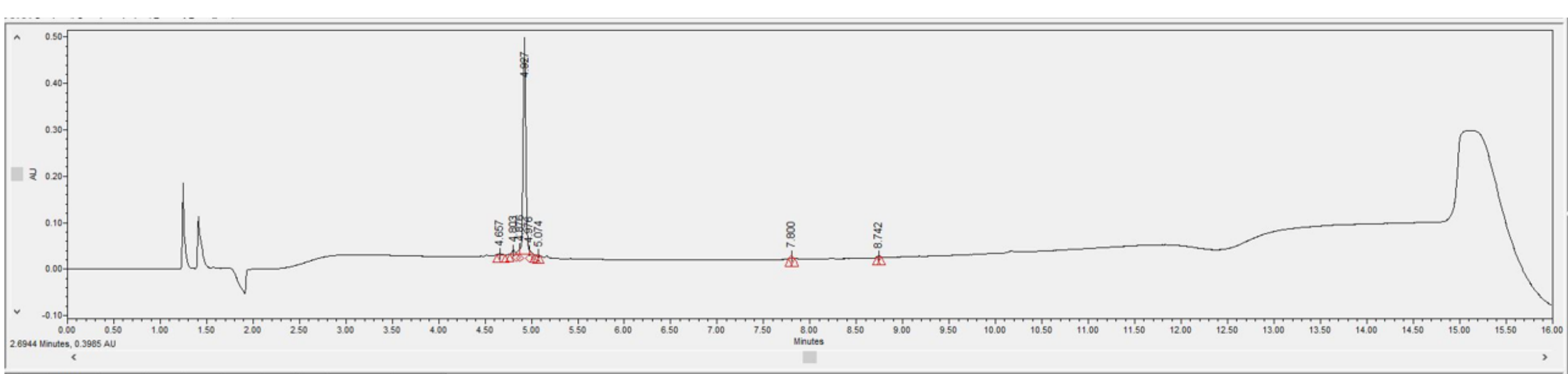

	Name	Retention Time (min)	Area (μV*sec)	% Area	Height (μV)	Int Type	Amount	Units	Peak Type	Peak Codes
1		4.657	6017	0.64	2472	bb			Unknown	
2		4.803	17487	1.87	8082	bv			Unknown	
3		4.876	16533	1.77	11534	vv			Unknown	I06
4		4.927	864521	92.36	454164	vv			Unknown	
5		4.976	13187	1.41	8363	vb			Unknown	I06
6		5.074	1656	0.18	1239	bb			Unknown	
7		7.800	12318	1.32	5878	bb			Unknown	
8		8.742	4317	0.46	2204	bb			Unknown	

G4

MALDI-TOF-MS: Calculated monoisotopic $[M+H]^+$ =3480.639; Observed monoisotopic $M+H]^+$ =3480.757

Analytical RP-HPLC (ESI-MS): H_2O/MeCN + 0.1% FA, 45-95% MeCN, 10 min, 0.3 mL/min; Agilent PLRP-S 300Å (3 µm, 2.1 x 50 mm) column.

Calculated monoisotopic $[M+3H]^{+3}$ = 1160.8850; $[M+4H]^{+4}$ = 870.9157; $[M+5H]^{+5}$ = 696.9341
Observed monoisotopic $[M+3H]^{+3}$ = 1161.5537; $[M+4H]^{+4}$ = 870.9167; $[M+5H]^{+5}$ = 696.9351

UPLC (220 nm): H_2O/MeCN + 0.1% TFA, 10-90% MeCN 0-10 min, wash and column equilibration 10-16 min, 0.3 mL/min; CSH C18 (1.7 µm, 2.1 x 150 mm) column.

Purity: 94.94%

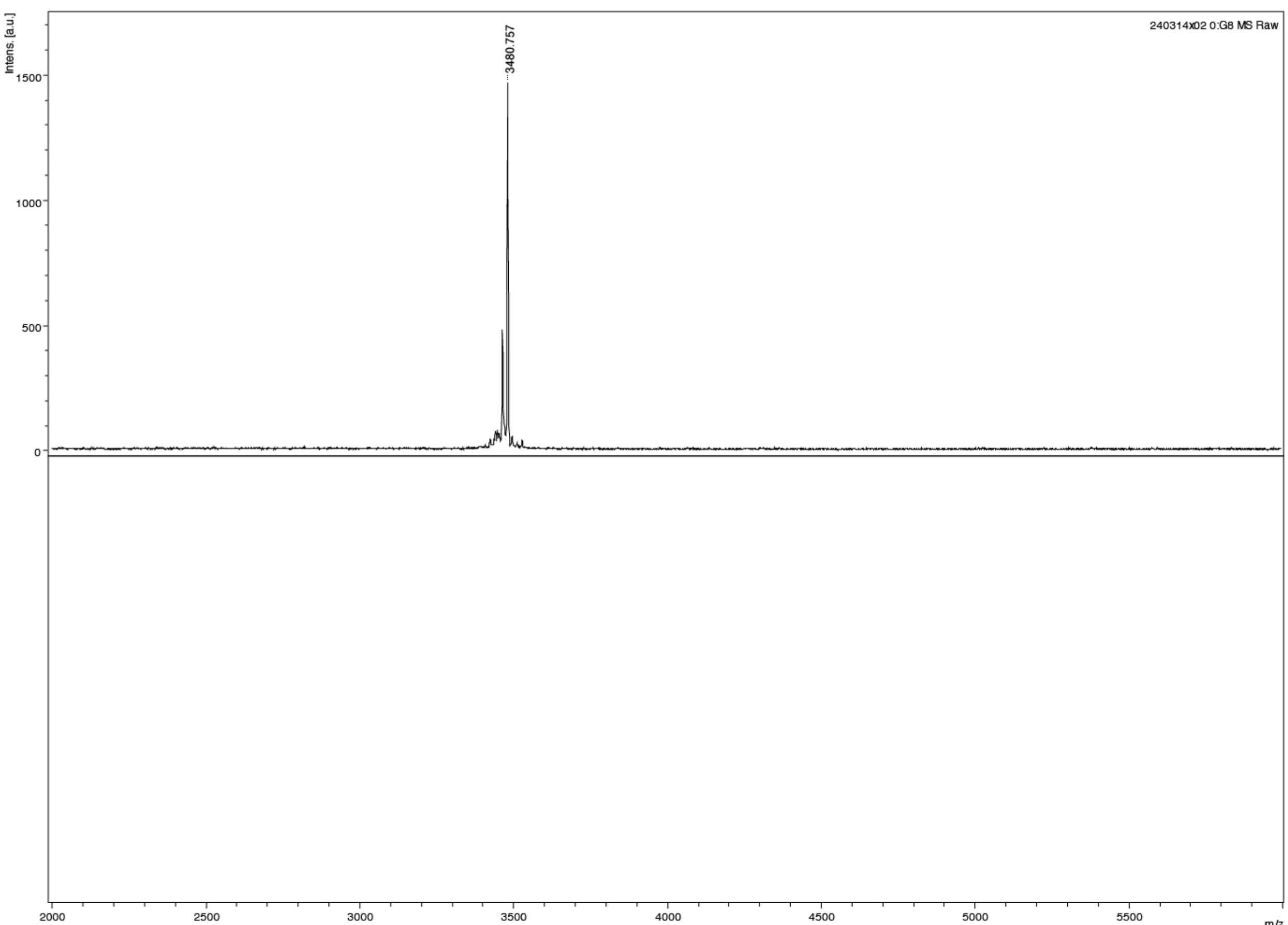

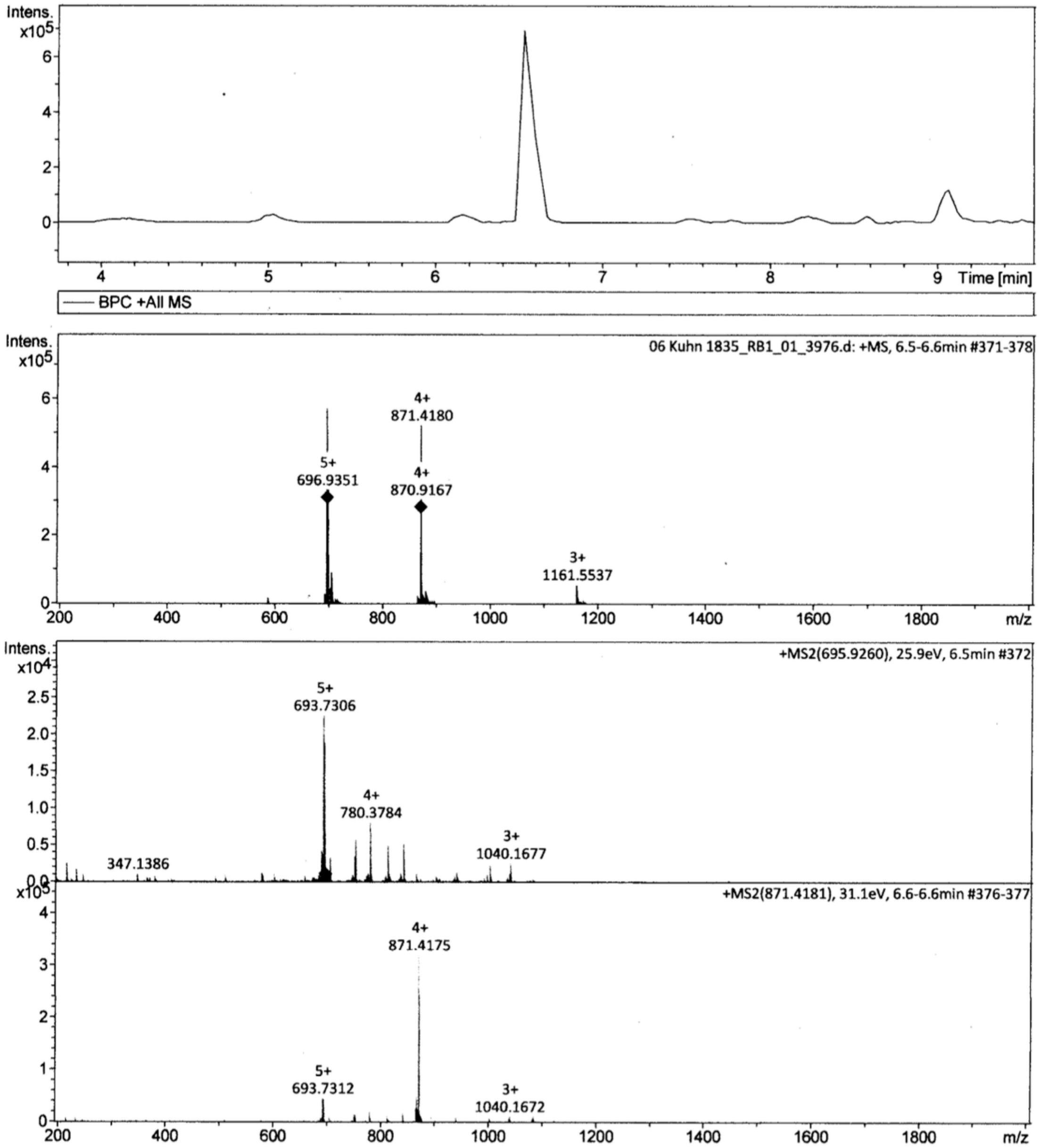

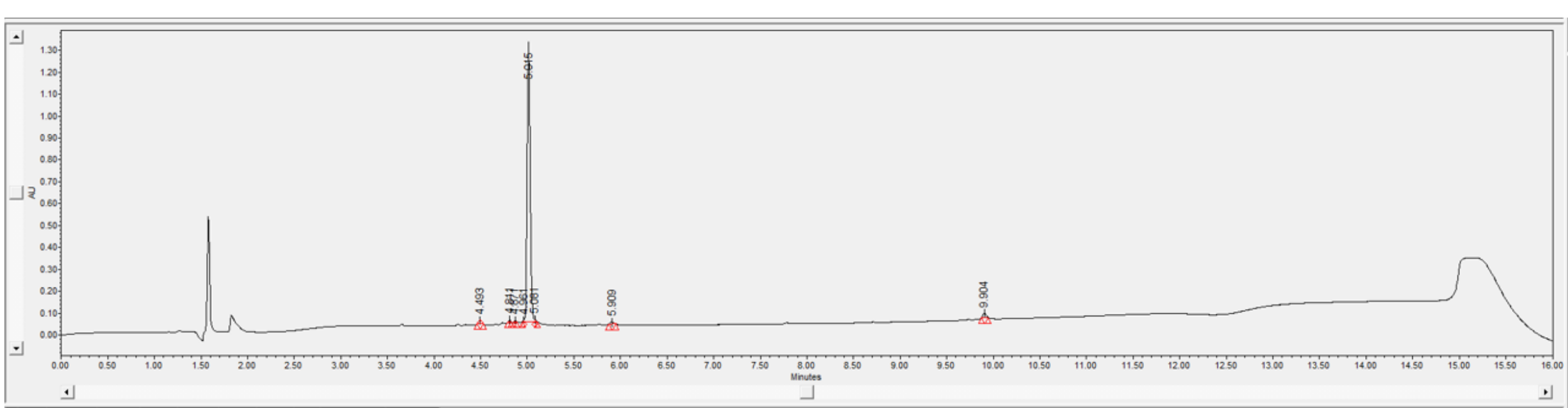

	Name	Retention Time (min)	Area (µV*sec)	% Area	Height (µV)	Int Type	Amount	Units	Peak Type	Peak Codes
1		4.493	36475	1.33	14653	bb			Unknown	
2		4.811	13705	0.50	10013	bb			Unknown	
3		4.871	5991	0.22	5300	bb			Unknown	
4		4.961	2367	0.09	6538	bv			Unknown	I06
5		5.015	2610655	94.94	1262443	vv			Unknown	
6		5.081	11505	0.42	10850	vb			Unknown	I06
7		5.909	22135	0.81	7853	bb			Unknown	
8		9.904	46875	1.70	19714	bb			Unknown	

GCGR antagonist (Peptide 11 reported by Yang et al.[8])

MALDI-TOF-MS: Calculated monoisotopic $[M+H]^+$ =2984.43; Observed monoisotopic $M+H]^+$ =2984.48

UPLC (220 nm): H_2O/MeCN + 0.1% TFA, 10-90% MeCN 0-10 min, wash and column equilibration 10-1 min, 0.3 mL/min; CSH C18 (1.7 µm, 2.1 x 150 mm) column.

Purity: 99.42%

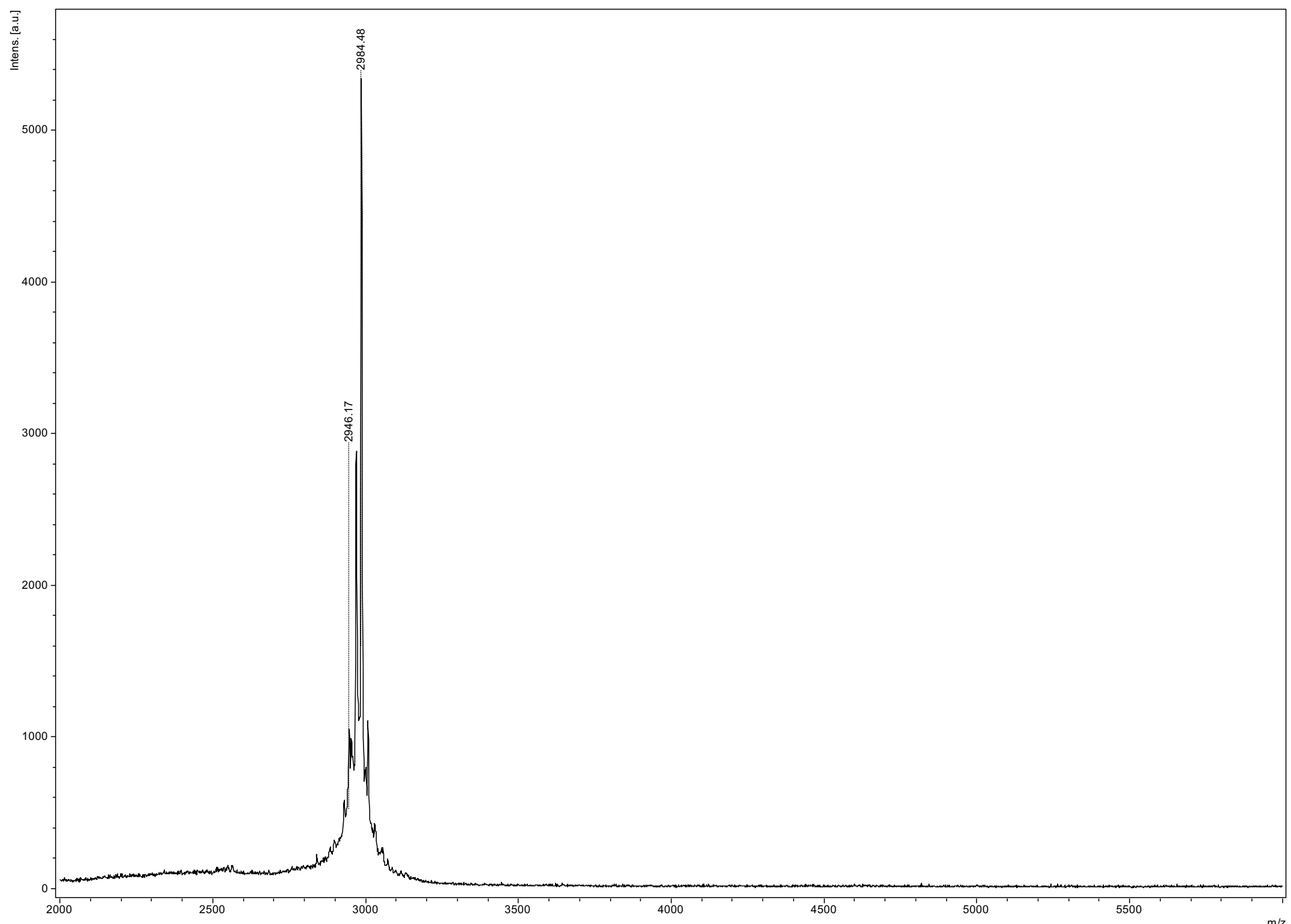

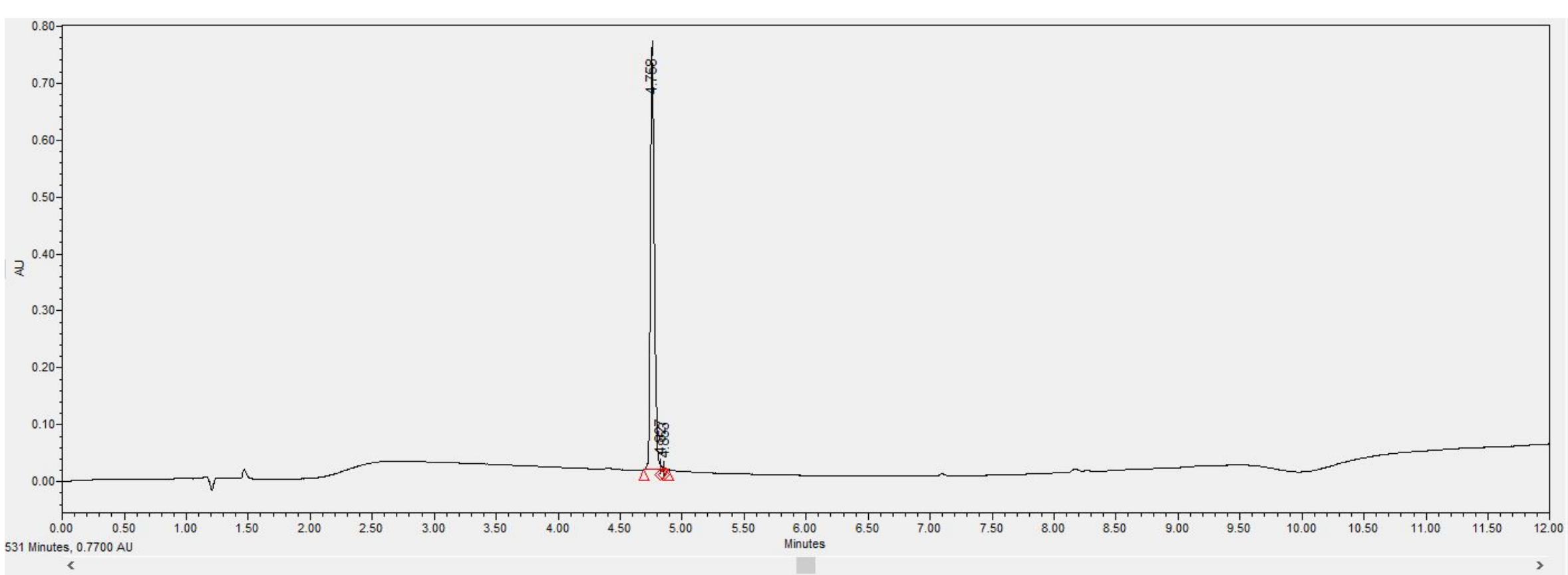

Name	Retention Time (min)	Area (µV*sec)	% Area	Height (µV)	Int Type	Amount	Units	Peak Type	Peak Codes
	4.758	1601912	99.42	740952	bv			Unknown	I37
	4.827	6633	0.41	6968	vv			Unknown	I37 I06 I21 I22 I23
	4.853	2717	0.17	1843	vb			Unknown	I37 I06 I21 I22 I23

PTH(1-34)

MALDI-TOF-MS and UPLC characterization of this peptide was reported previously[3].

P1

MALDI-TOF-MS: Calculated monoisotopic $[M+H]^+$ =4115.154; Observed monoisotopic $M+H]^+$ =4113.957

UPLC (220 nm): H_2O/MeCN + 0.1% TFA, 10-60% MeCN 0-10 min, wash and column equilibration 10-15 min, 0.3 mL/min; CSH C18 (1.7 µm, 2.1 x 100 mm) column.

Purity: 96.65%

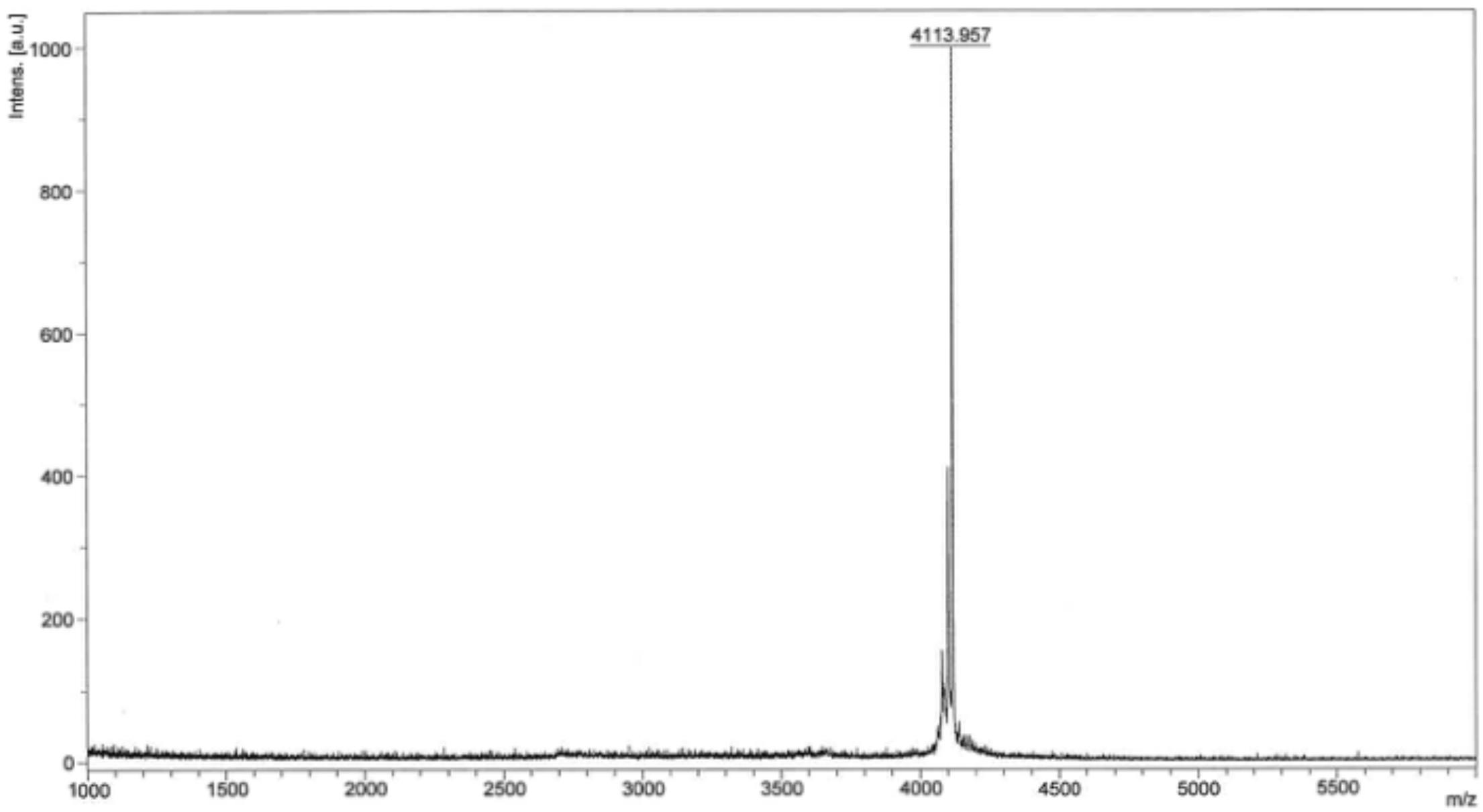

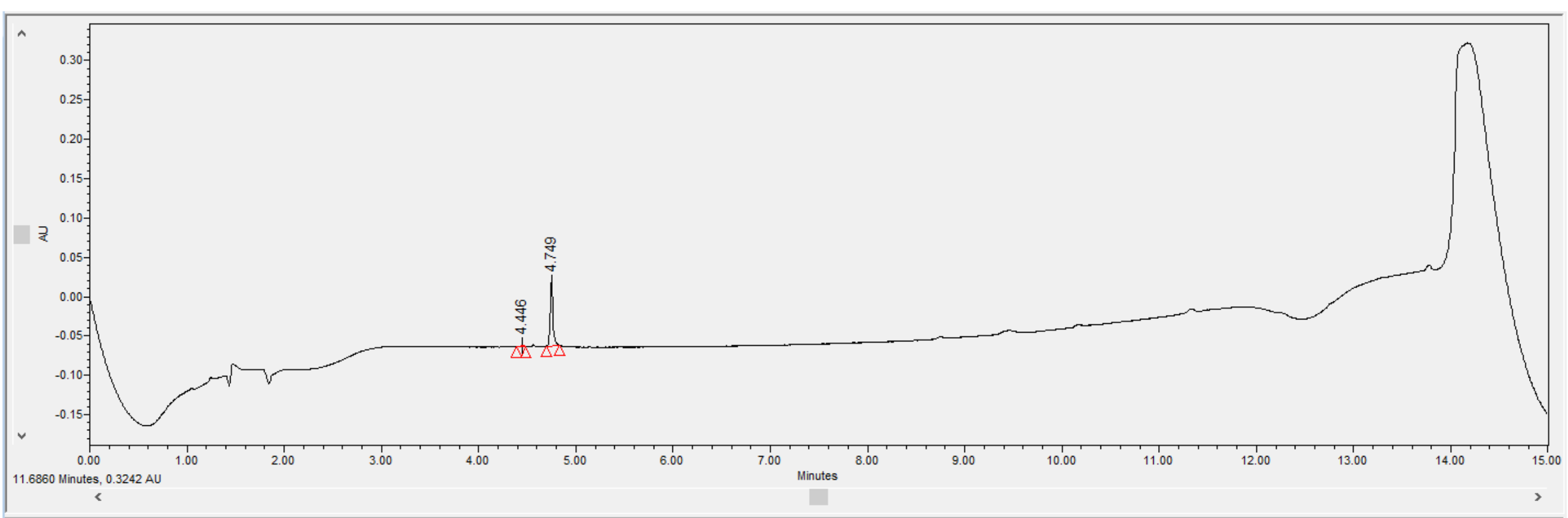

	Name	Retention Time (min)	Area (µV*sec)	% Area	Height (µV)	Int Type	Amount	Units	Peak Type	Peak Codes
1		4.446	5498	3.35	2248	bb			Unknown	
2		4.749	158469	96.65	81198	bb			Unknown	

P2

MALDI-TOF-MS: Calculated monoisotopic $[M+H]^+$ =4129.170; Observed monoisotopic $M+H]^+$ =4127.629

UPLC (220 nm): H_2O/MeCN + 0.1% TFA, 10-60% MeCN 0-10 min, wash and column equilibration 10-15 min, 0.3 mL/min; CSH C18 (1.7 µm, 2.1 x 100 mm) column.

Purity: 93.54%

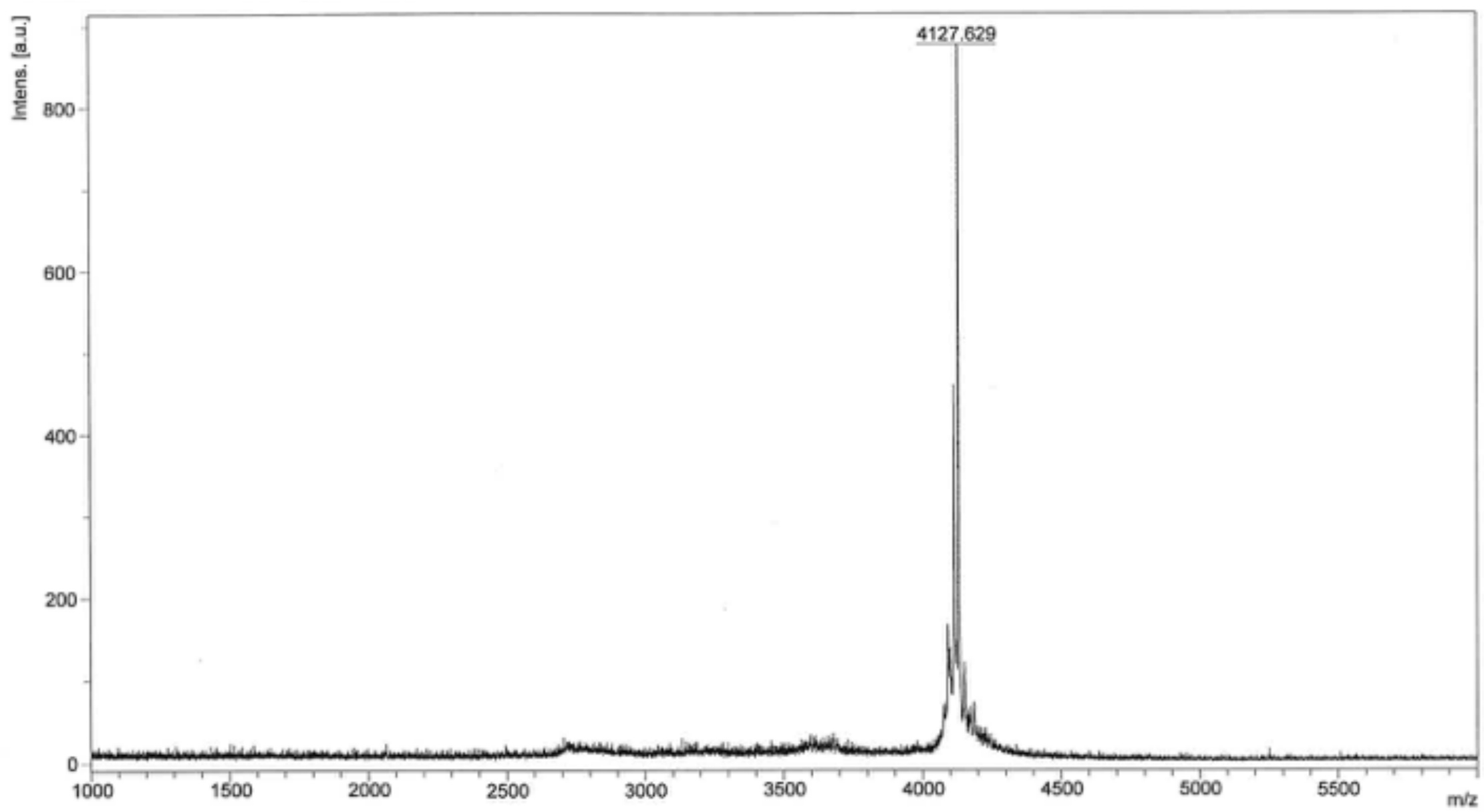

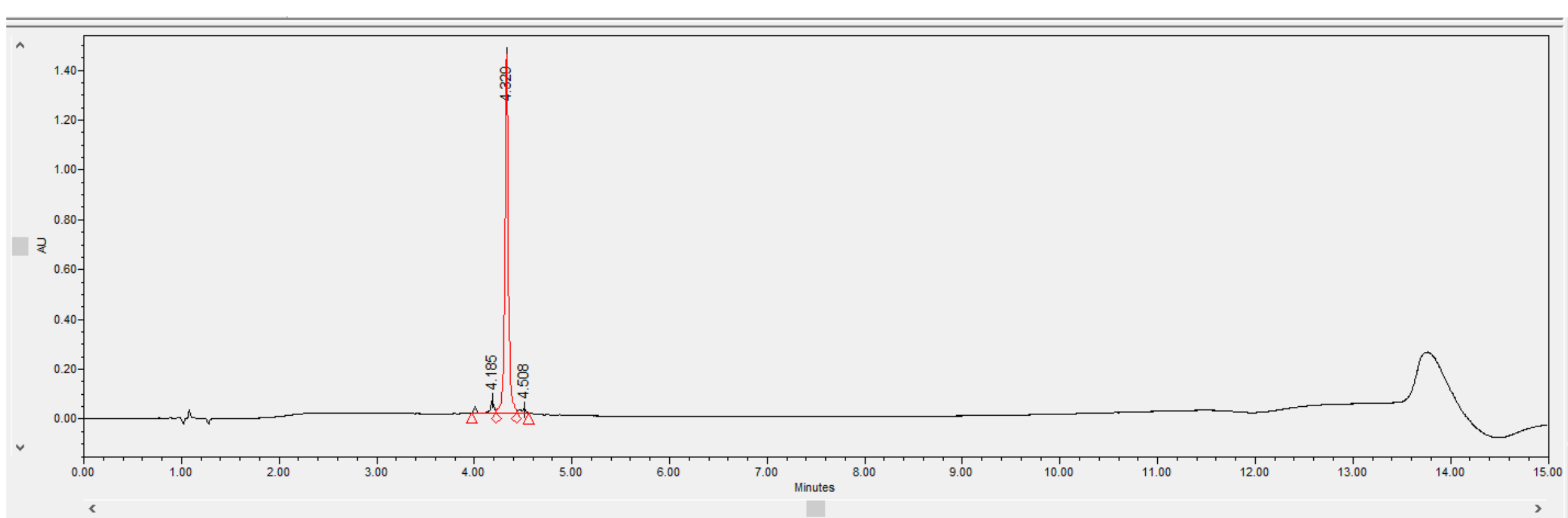

	Name	Retention Time (min)	Area (µV*sec)	% Area	Height (µV)	Int Type	Amount	Units	Peak Type	Peak Codes
1		4.185	166856	4.45	50941	bv			Unknown	
2		4.329	3504833	93.54	1437409	vv			Unknown	
3		4.508	75291	2.01	15205	vb			Unknown	

P3

MALDI-TOF-MS: Calculated monoisotopic $[M+H]^+ = 4115.154$; Observed monoisotopic $M+H]^+ = 4114.115$

UPLC (220 nm): H_2O/MeCN + 0.1% TFA, 10-60% MeCN 0-10 min, wash and column equilibration 10-15 min, 0.3 mL/min; CSH C18 (1.7 µm, 2.1 x 100 mm) column.

Purity: >99%

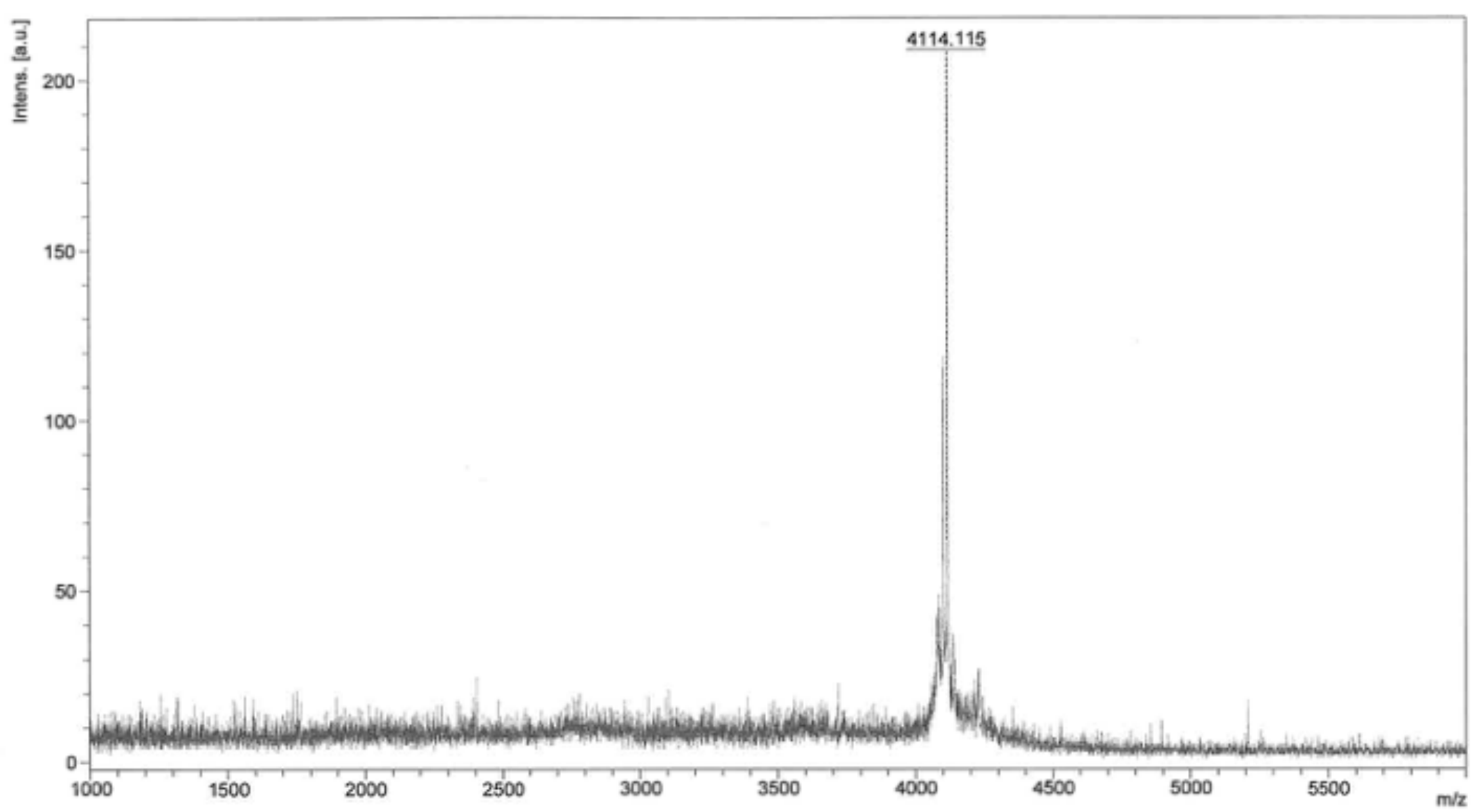

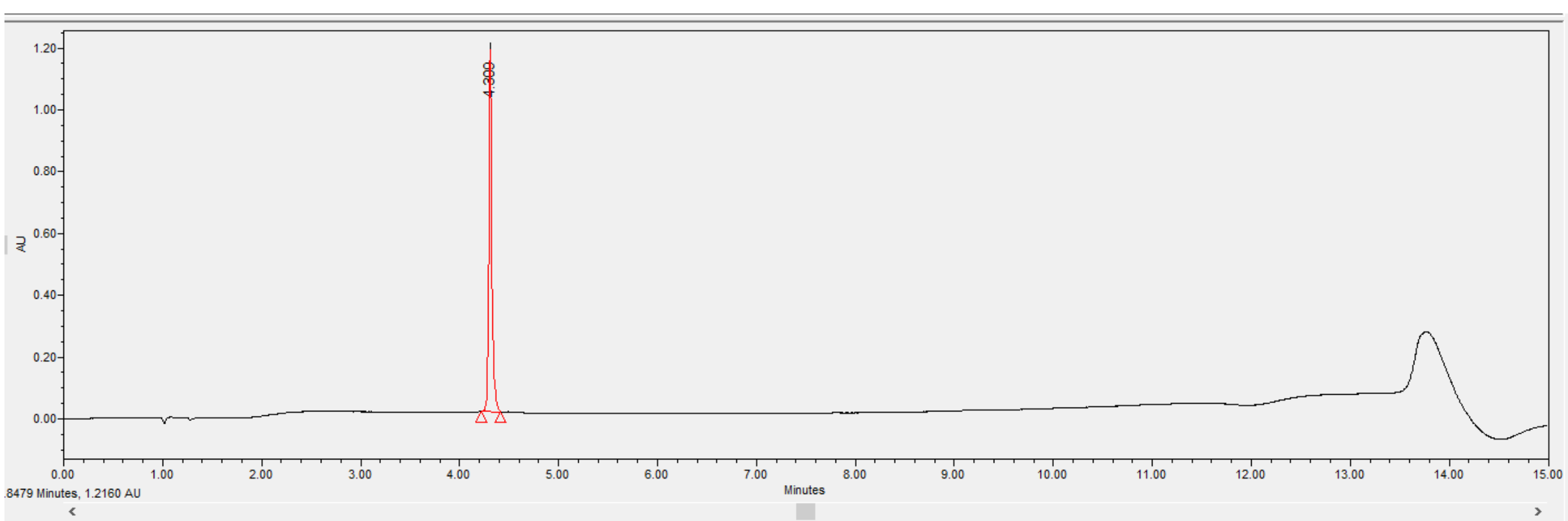

Name	Retention Time (min)	Area (µV*sec)	% Area	Height (µV)	Int Type	Amount	Units	Peak Type	Peak Codes
	4.309	2516819	100.00	1168710	bb			Unknown	

P2(1-35)-(Lys35-tetramethylrhodamine)-NH_2

MALDI-TOF-MS: Calculated monoisotopic $[M+H]^+$ =4669.40; Observed monoisotopic $M+H]^+$ =4669.49

UPLC (220 nm): H_2O/MeCN + 0.1% TFA, 10-60% MeCN 0-10 min, wash and column equilibration 10-15 min, 0.3 mL/min; CSH C18 (1.7 µm, 2.1 x 100 mm) column.

Purity: 99.10%

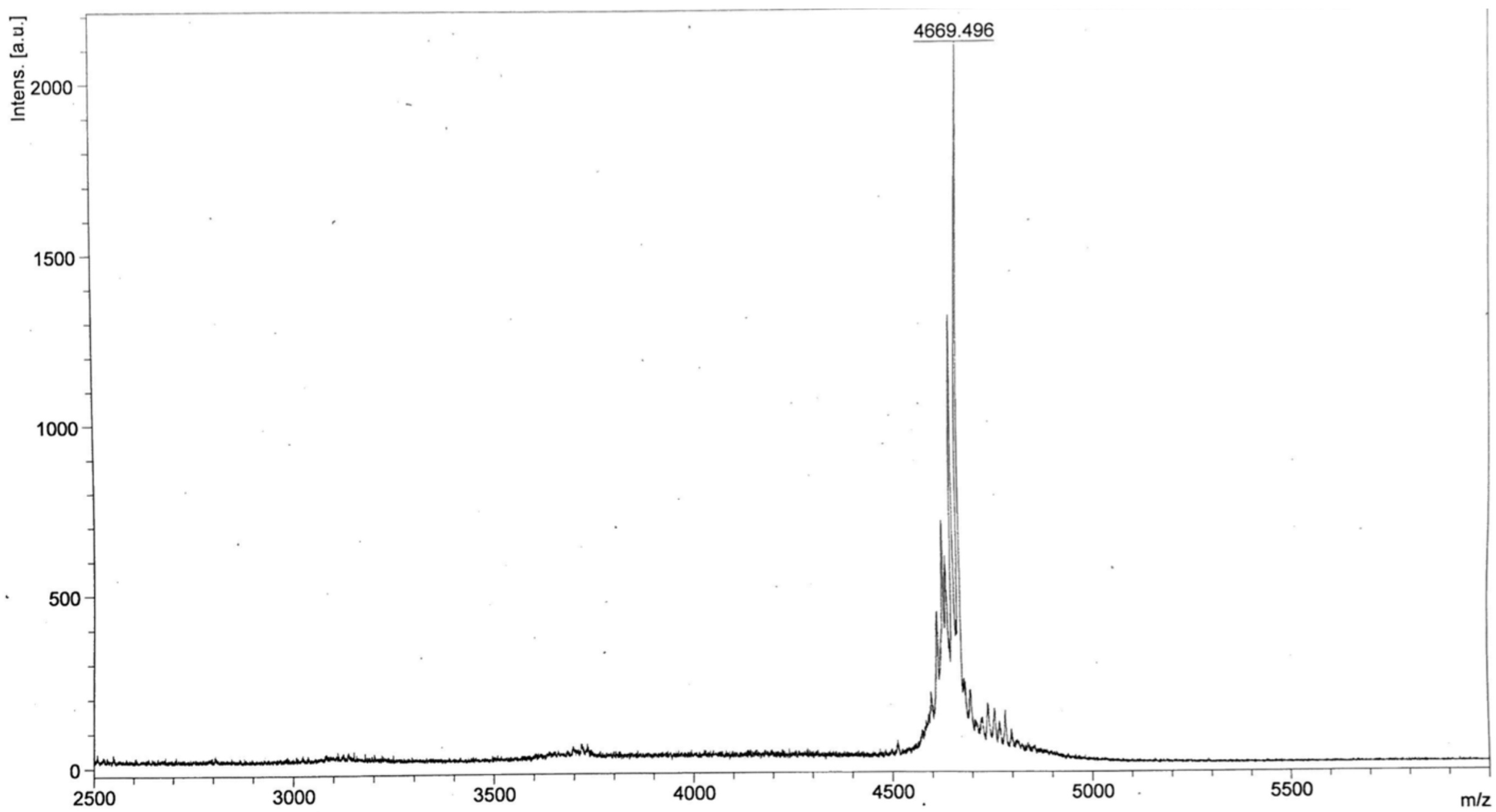

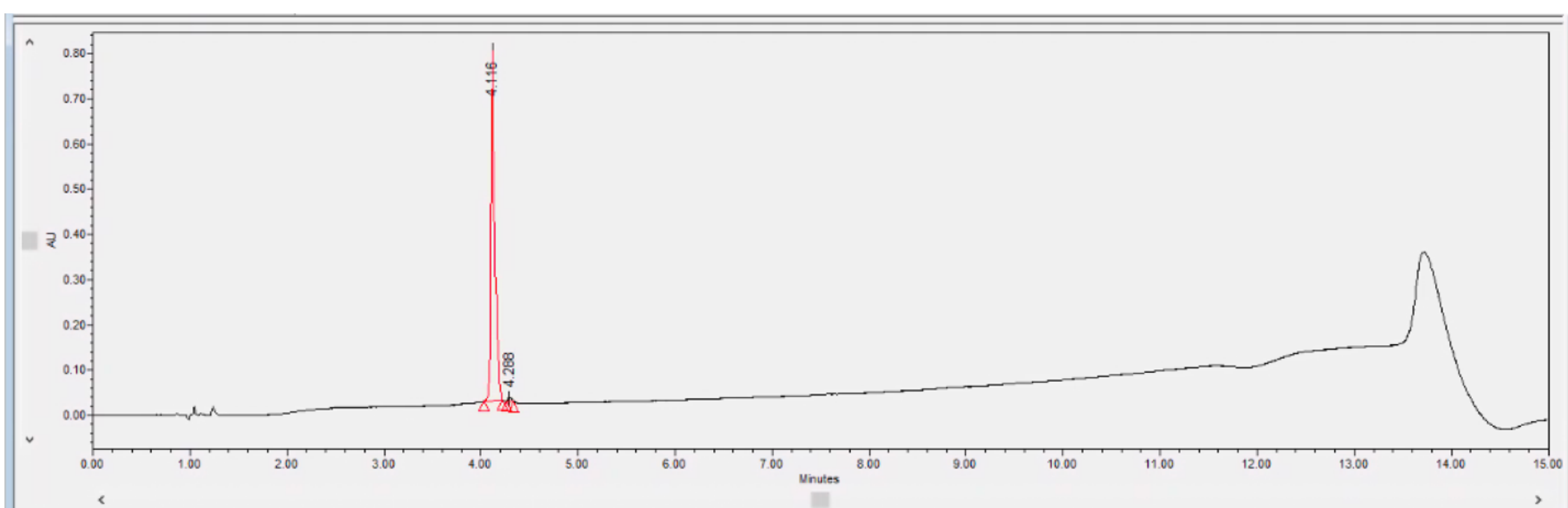

	Name	Retention Time (min)	Area (µV*sec)	% Area	Height (µV)	Int Type	Amount	Units	Peak Type	Peak Codes
1		4.116	2038196	99.10	770735	bb			Unknown	
2		4.288	18413	0.90	6751	bb			Unknown	

PTH(1-35)-(Lys35-tetramethylrhodamine)-NH_2

This peptide, used as a tracer for NLuc-PTHR1 binding experiments, was reported previously[3].

IV: Supplementary References

1. Yu, Z. *et al.* Kinetic and Thermodynamic Insights into Agonist Interactions with the Parathyroid Hormone Receptor-1 from a New NanoBRET Assay. *ACS Chem. Biol.* **17**, 3148–3158 (2022).

2. Liu, S. *et al.* Use of Backbone Modification to Enlarge the Spatiotemporal Diversity of Parathyroid Hormone Receptor-1 Signaling via Biased Agonism. *J. Am. Chem. Soc.* **141**, 14486–14490 (2019).

3. Gibadullin, R., Kim, T. W., Tran, L. M. L. & Gellman, S. H. Hormone Analogues with Unique Signaling Profiles from Replacement of α-Residue Triads with β/γ Diads. *J. Am. Chem. Soc.* **145**, 20539–20550 (2023).

4. Hager, M. V., Johnson, L. M., Wootten, D., Sexton, P. M. & Gellman, S. H. β-Arrestin-Biased Agonists of the GLP-1 Receptor from β-Amino Acid Residue Incorporation into GLP-1 Analogues. *J. Am. Chem. Soc.* **138**, 14970–14979 (2016).

5. Kenakin, T., Watson, C., Muniz-Medina, V., Christopoulos, A. & Novick, S. A simple method for quantifying functional selectivity and agonist bias. *ACS Chem. Neurosci.* **3**, 193–203 (2012).

6. Black, J. W. & Leff, P. Operational models of pharmacological agonism. *Proceedings of the Royal Society of B: Biological Sciences* **220**, 141–162 (1983).

7. Checco, J. W., Eddinger, G. A., Rettko, N. J., Chartier, A. R. & Gellman, S. H. Tumor Necrosis Factor-α Trimer Disassembly and Inactivation via Peptide-Small Molecule Synergy. *ACS Chem. Biol.* **15**, 2116–2124 (2020).

8. Yang, B. *et al.* Optimization of Truncated Glucagon Peptides to Achieve Selective, High Potency, Full Antagonists. *J. Med. Chem.* **64**, 4697–4708 (2021).
