# Supplementary material for: Potent and biased agonists of class B1 GPCRs from a heterochiral design strategy
Source: Nat Chem. 2026 Jun 16;18(9):1639–49. doi: 10.1038/s41557-026-02182-x (PMC13524830; doi:10.1038/s41557-026-02182-x)
Supplement: Supplementary file 2 [file 41557_2026_2182_MOESM2_ESM.pdf]

nature portfolio

Corresponding author(s): Samuel H. Gellman, Giuseppe Deganutti

Last updated by author(s): Apr 1, 2026

Reporting Summary

Nature Portfolio wishes to improve the reproducibility of the work that we publish. This form provides structure for consistency and transparency in reporting. For further information on Nature Portfolio policies, see our Editorial Policies and the Editorial Policy Checklist.

Statistics

For all statistical analyses, confirm that the following items are present in the figure legend, table legend, main text, or Methods section.

n/a	Confirmed	
☐	☒	The exact sample size (*n*) for each experimental group/condition, given as a discrete number and unit of measurement
☐	☒	A statement on whether measurements were taken from distinct samples or whether the same sample was measured repeatedly
☐	☒	The statistical test(s) used AND whether they are one- or two-sided<br>*Only common tests should be described solely by name; describe more complex techniques in the Methods section.*
☒	☐	A description of all covariates tested
☐	☒	A description of any assumptions or corrections, such as tests of normality and adjustment for multiple comparisons
☐	☒	A full description of the statistical parameters including central tendency (e.g. means) or other basic estimates (e.g. regression coefficient) AND variation (e.g. standard deviation) or associated estimates of uncertainty (e.g. confidence intervals)
☐	☒	For null hypothesis testing, the test statistic (e.g. *F*, *t*, *r*) with confidence intervals, effect sizes, degrees of freedom and *P* value noted<br>*Give P values as exact values whenever suitable.*
☒	☐	For Bayesian analysis, information on the choice of priors and Markov chain Monte Carlo settings
☒	☐	For hierarchical and complex designs, identification of the appropriate level for tests and full reporting of outcomes
☒	☐	Estimates of effect sizes (e.g. Cohen's *d*, Pearson's *r*), indicating how they were calculated

Our web collection on statistics for biologists contains articles on many of the points above.

Software and code

Policy information about availability of computer code

Data collection	ACEMD3.7, Plumed 2.7
Data analysis	GraphPad Prism 10, Microsoft PowerPoint, Microsoft Excel, ChemDraw Prime 16.0, HTMD2.3.2, VMD1.9.2, AmberTools20, UCSF Chimera1.14, GetContacts

For manuscripts utilizing custom algorithms or software that are central to the research but not yet described in published literature, software must be made available to editors and reviewers. We strongly encourage code deposition in a community repository (e.g. GitHub). See the Nature Portfolio guidelines for submitting code & software for further information.

Data

Policy information about availability of data

All manuscripts must include a data availability statement. This statement should provide the following information, where applicable:

- Accession codes, unique identifiers, or web links for publicly available datasets
- A description of any restrictions on data availability
- For clinical datasets or third party data, please ensure that the statement adheres to our policy

Data reported in this study are available in the article, Supplementary Information, and Source Data files. Previously published structural models used in Supplementary Fig. 2 are available from the Protein Data Bank under accession numbers 6LMK and 8FLQ. MD simulations can be downloaded from the public

repository Zenodo: https://zenodo.org/records/19096161106. Information and requests for data or materials/reagents may be directed to the corresponding authors, G.D. (ad5288@coventry.ac.uk) or S.H.G. (gellman@chem.wisc.edu).

Human research participants

Policy information about studies involving human research participants and Sex and Gender in Research.

Reporting on sex and gender	This information has not been collected
Population characteristics	Human research participants were not used
Recruitment	Recruitment was not performed
Ethics oversight	Ethics oversight was not pursued.

Note that full information on the approval of the study protocol must also be provided in the manuscript.

Field-specific reporting

Please select the one below that is the best fit for your research. If you are not sure, read the appropriate sections before making your selection.

☒ Life sciences ☐ Behavioural & social sciences ☐ Ecological, evolutionary & environmental sciences

For a reference copy of the document with all sections, see nature.com/documents/nr-reporting-summary-flat.pdf

Life sciences study design

All studies must disclose on these points even when the disclosure is negative.

Sample size	A minimum of 3 biological replicates for all in cellulo assays was determined at the outset, with additional replicates performed for those assays used for bias factor calculations, which propagate error. A minimum 3 biological replicates is the standard for in cellulo assays such as these and provide sufficient confidence for statistical analyses in the bias factor calculations.<br>The sample sizes of GCG-NH2 and PTH(1-34) are occasionally larger than others due to their use as a standard for normalization across experiments.
Data exclusions	No data were excluded.
Replication	All experiment results were replicable. Information on how often experiments were replicated or performed independently is provided in detail within figure legends.
Randomization	Randomization was not required.
Blinding	Blinding was not relevant to our studies given that all data collection and analysis involved objective measurements.

Reporting for specific materials, systems and methods

We require information from authors about some types of materials, experimental systems and methods used in many studies. Here, indicate whether each material, system or method listed is relevant to your study. If you are not sure if a list item applies to your research, read the appropriate section before selecting a response.

Materials & experimental systems

n/a	Involved in the study	
☒	☐	Antibodies
☐	☒	Eukaryotic cell lines
☒	☐	Palaeontology and archaeology
☒	☐	Animals and other organisms
☒	☐	Clinical data
☒	☐	Dual use research of concern

Methods

n/a	Involved in the study	
☒	☐	ChIP-seq
☒	☐	Flow cytometry
☒	☐	MRI-based neuroimaging

Eukaryotic cell lines

Policy information about cell lines and Sex and Gender in Research

Cell line source(s)	HEK293GS22 (GloSensor) Cells (Promega E1261; kind gift from Thomas Gadella), HEK293FT Cells (Thermo Fisher; R70007), HEK293 (PathHunter® eXpress GCGR Total GPCR Internalization Assay; Eurofins DiscoverX Products Company 98-0820E1CP0M), HEK293 GP2.3 (PTH1R + GloSensor) Cells (Promega E1261; kind gift from Thomas Gardella), HEK293H (+NLuc-PTH1R) Cells (in house previously), CHO-FlpIn (+PTH1R-RLuc8 and β-arrestin-1-Venus) Cells (kind gift from Denise Wooten and Patrick Sexton labs), CHO-FlpIn (+PTH1R-RLuc8 and β-arrestin-2-Venus) Cells (kind gift from Denise Wooten and Patrick Sexton labs)
Authentication	No authentication was required
Mycoplasma contamination	The cell lines were not tested for mycoplasma
Commonly misidentified lines (See ICLAC register)	The cell lines are not listed in the database
